# Unraveling the YAP1-TGFβ1 axis: a key driver of androgen receptor loss in prostate cancer-associated fibroblasts

**DOI:** 10.1186/s13046-025-03578-2

**Published:** 2025-12-01

**Authors:** Elena Brunner, Elisabeth Damisch, Melanie Emma Groninger, Francesco Baschieri, François Tyckaert, Lukas Nommensen, Lucy Neumann, Georgios Fotakis, Zlatko Trajanoski, Georg Schäfer, Martin Puhr, Isabel Heidegger, Michael J. Ausserlechner, Christian Ploner, Sofia Karkampouna, Francesco Bonollo, Marianna Kruithof-de Julio, Natalie Sampson

**Affiliations:** 1 Department of Urology, Medical University of Innsbruck, Innsbruck, 6020 Austria; 2 Department of Pediatrics I, 3D Bioprinting Core Facility, Medical University of Innsbruck, Innsbruck, 6020 Austria; 3 Institute of Pathophysiology, Biocenter, Medical University of Innsbruck, Innsbruck, 6020 Austria; 4 Department of Internal Medicine V, Medical University of Innsbruck, Innsbruck, 6020 Austria; 5 Institute of Bioinformatics, Medical University of Innsbruck, Innsbruck, 6020 Austria; 6 Institute of Pathology, Neuropathology and Molecular Pathology, Medical University of Innsbruck, Innsbruck, 6020 Austria; 7 Department of Plastic, Reconstructive and Aesthetic Surgery, Medical University of Innsbruck, Innsbruck, 6020 Austria; 8 Department for BioMedical Research, Urology Research Laboratory, University of Bern, Bern, Switzerland; 9 Department of Urology, Inselspital, Bern University Hospital, University of Bern, Bern, Switzerland

**Keywords:** CAF, Tumor microenvironment, Patient-derived xenograft, Stroma, Autophagy

## Abstract

Due to their pivotal roles in tumor progression and therapy resistance, cancer-associated fibroblasts (CAF) are considered key therapeutic targets with loss of stromal androgen receptor (AR) a poorly understood hallmark of aggressive prostate cancer (PCa). A paucity of pre-clinical models however has hampered functional studies of CAF heterogeneity. We demonstrate that our newly generated CAF biobank contains three FAP^+^-fibroblast subtypes, each with unique molecular and functional traits. Cultures with an early-activated phenotype expressed the highest levels of AR and exhibited AR-dependent growth, whereby AR inhibition suppressed their migration. Consistently, stromal cells expressing early-activation markers co-expressed nuclear AR in clinical specimens and were enriched in pre-neoplastic lesions/low-grade PCa. Conversely, myofibroblastic CAF (myCAF) expressed low AR levels in vitro and in vivo*,* were insensitive at the proliferative and migratory levels to AR signaling modulation and significantly promoted PCa cell invasion in 3D composite collagen networks. Accordingly, myCAF constituted the predominant CAF subpopulation in stromogenic high-grade PCa and were enriched in aggressive disease states in PCa single cell atlases and castration-resistant LACP9 patient-derived xenografts. Exacerbation of the myCAF state upon castration of LAPC9-bearing hosts underscored these findings. Mechanistically, AR loss in myCAF was driven by an NFκB-TGFβ1-YAP1 axis, whose combined pharmacological or genetic targeting synergistically repressed myofibroblastic hallmarks and impaired autophagic flux, effects that were potentiated by enzalutamide resulting in enhanced myCAF cell death. Collectively, data herein provide a mechanistic rationale for stromal AR loss in aggressive PCa and suggest that adjuvant targeting of the YAP1-TGFβ signaling axis may improve patient outcome.

**Supplementary Information:**

The online version contains supplementary material available at 10.1186/s13046-025-03578-2.

## Introduction

Prostate homeostasis is maintained via extensive stromal-epithelial crosstalk [1]. Perturbation via pathological processes, including prostatic intraepithelial neoplasia (PIN) and prostate cancer (PCa), lead to a stromogenic reaction characterized by deposition of a collagen-rich extracellular matrix (ECM), SMC dedifferentiation and the emergence of cancer-associated fibroblasts (CAF) [1]. CAF represent a central onco-modulatory component of the tumor microenvironment (TME) that influence PCa progression and therapy response via diverse mechanisms [2]. Since stromal activation occurs early during disease development and positively correlates with parameters of poor prognosis in PCa [3], there is considerable interest in developing stromal-targeted therapies.

Treatment options for advanced prostate cancer (PCa) are broadly categorized into radiotherapy, taxane-based chemotherapeutics and suppression of the androgen signaling axis via androgen deprivation therapy (ADT) and androgen receptor signaling inhibitors (ARSI) [4]. While chemotherapy and radiation primarily target highly proliferating cancer cells, ADT and ARSI aim to restrict PCa cells, whose proliferation is dependent on signaling by the androgen receptor (AR), an androgen-responsive transcription factor. Besides luminal epithelial cells, prostatic AR is also expressed by fibroblasts, activated endothelial cells and parenchymal smooth muscle cells (SMC) [5], the latter constituting the most abundant stromal cell type of the benign prostate. In PCa however, there is a well-characterized but poorly understood loss of stromal AR, which correlates with poor prognosis [5]. Together with the low stromal proliferative index, current systemic treatments therefore fail to specifically target the stromal component of disease. In stark contrast to the well-characterized pro-proliferative role of epithelial AR, stromal AR is less well understood and appears to exert tumor-suppressive functions [6–8]. The current lack of prostatic stromal cell models expressing endogenous AR however has limited our understanding of stromal AR function, an issue that must be overcome to clarify the clinical implications regarding potential inadvertent effects of ADT/ARSI on onco-suppressive stromal AR signaling.

CAF are highly heterogeneous and both tumor-suppressive and tumor-promoting subpopulations have been described [2]. The prevailing consensus is that fibroblasts reside on an activation continuum whereby tumor stage and spatial signaling cues play key roles in determining inflammatory (inflammatory CAF, iCAF) and matrix-producing contractile CAF (myofibroblastic CAF, myCAF) phenotypes [9, 10]. Antigen-presenting CAF (apCAF) constitute a third but lesser understood CAF subpopulation that may represent an intermediate iCAF substate [10]. Whilst myCAF populations are universally associated with aggressive disease, functional analyses of CAF subpopulations are largely lacking, primarily due to the lack of appropriate experimental models.

This study aimed to generate an experimentally versatile fibroblast biobank encompassing fibroblast subpopulations from clinical PCa tissues. The biobank generated contains three distinct primary human prostate FAP^+^ fibroblast subtypes displaying molecular parallels to early-activated fibroblasts (iCAF), those in an intermediate or transitioning phase, and late-activated myCAF substates. High AR expression and sensitivity of the early-activated clinically favorable fibroblastic state to AR signaling modulation contrasted with the low AR expression of late-activated onco-supportive myCAF, which were insensitive to AR signaling modulation and androgen deprivation in regard to proliferation and migration and significantly promoted PCa cell invasion in 3D collagen networks. These findings were supported by studies of clinical specimens and castration-resistant patient-derived xenograft (PDX) models and have potential implications for current ADT strategies. We identified TGFβ1-YAP1 signaling as a central driver of AR loss and acquisition of the late-activated myCAF state whereby cooperative signaling by NFkB further supports this process. Combined targeting of this signaling axis in myCAF cultures synergistically attenuated their myofibroblastic traits and was associated with impaired autophagic flux. These findings illustrate the translational value of the biobank and suggest that adjuvant targeting of the TGFβ1-YAP1 signaling axis may improve patient response.

## Results

### Generation of a biobank comprising distinct primary prostate fibroblast substates

To better functionally characterize fibroblast heterogeneity within the PCa microenvironment, we developed a biobank of primary fibroblast explant cultures isolated from PCa-enriched and benign-adjacent surgical resections (Supplemental Fig. S1). This biobank currently comprises 396 cultures from 177 biopsy cores of 95 different patients and includes patient-matched cultures from malignant and benign-adjacent regions from 64 different patients (Supplemental Table 1). All cultures tested uniformly expressed mesenchymal markers vimentin and THY1/CD90 but lacked the epithelial marker pan-cytokeratin (Supplemental Fig. S1−2).

**Fig. 1:**
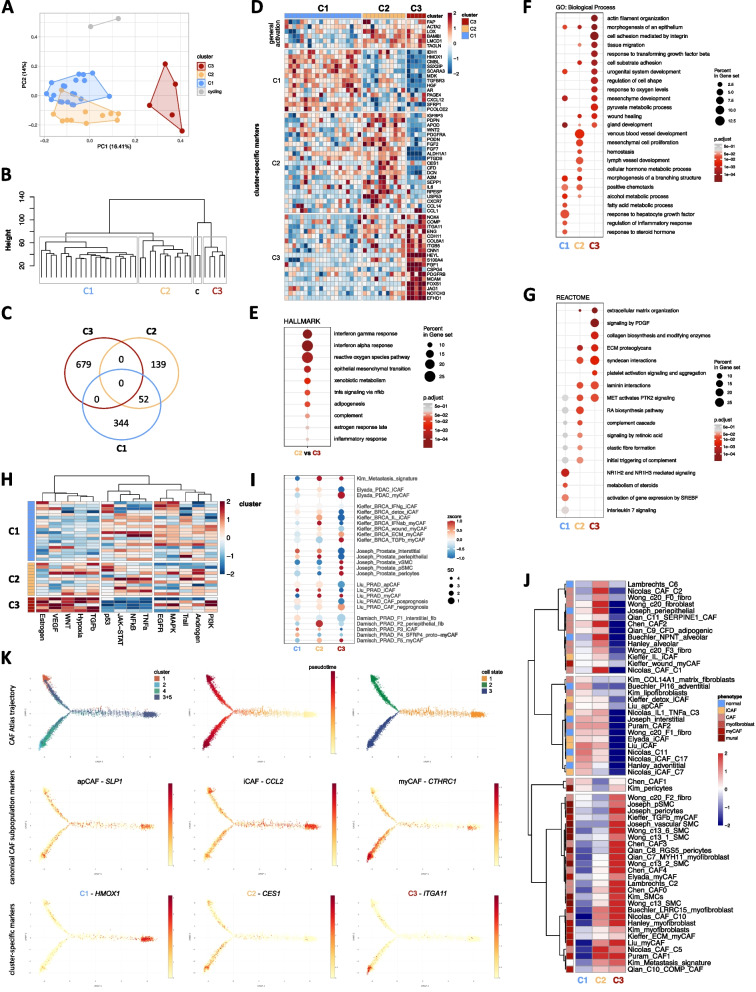
Expression profiling of primary prostate explant cultures identifies distinct fibroblast subpopulations. **A**-**H** Expression profiling of 38 patient-matched primary prostate explant cultures under basal culture conditions. **A** PCA analysis, **B** unsupervised hierarchical clustering (c, cycling CAF), **C** Venn diagram of differentially regulated genes and **D** sample level expression of selected genes significantly differentially regulated in each subcluster. **E** Hallmark pathways for genes significantly upregulated in C2 *vs.* C3 cultures. **F** GO Biological Process and **G** Reactome pathways for genes significantly differentially regulated in each subcluster. **H** PROGENy inferred signaling pathway activities of each subcluster based on significantly differentially expressed genes in each subcluster. **I** Dotplot comparison and **J** heatmap with rows clustered by correlation to published scRNA-seq datasets using combined z-scores and represented as mean per fibroblast cluster. **K** Inferred pseudotime trajectory of CAF activation for the indicated substate-delineating genes in cells annotated as CAF in the pan-cancer single cell CAF Atlas [12, 13]. For orientation, the distribution of the original fibroblast clusters, pseudotime and cell activation state (top panels) and expression of canonical CAF subtype markers (middle panels) are depicted. Source data for panels **C**, **E**-**J** are provided in the Source Data file

Morphological and phenotypic heterogeneity among these cultures prompted us to discern the transcriptomic profile of thirty-eight randomly selected patient-matched cultures (Fig. 1; Supplemental Fig. S3A-B). Bioinformatic analyses identified four distinct clusters (Fig. 1A-C). One cluster (“cycling”) comprising just two samples substantially deviated from the other clusters and was enriched for gene ontology (GO) terms related to cell division (Supplemental Fig. S3B) similar to previously reported “cycling CAF” [11]. These samples were not analyzed further. *VIM* was uniformly expressed across the remaining three clusters (C1-C3), however canonical CAF marker expression differed significantly (Fig. 1D, Supplemental Fig. S3C-D).

C1 displayed low expression levels of archetypal CAF markers, upregulation of HGF response and MAPK/EGFR signaling, and was enriched for gene sets related to development, morphogenesis and metabolic pathways, (Fig. 1D-H, Supplemental Fig. S3B) suggesting a homeostatic function typically associated with benign prostate fibroblasts. Supportively, C1 exhibited similarity to Buechler *PI16* steady-state “universal” fibroblasts [14] and Nicolas C11 benign fibroblasts [15] (Fig. 1I-J, Supplemental Fig. S3E). Similarity to some iCAF-associated signatures however suggested an early activation/pro-inflammatory phenotype that is frequently observed in tumor-adjacent benign tissue [16]. Indeed, projecting C1 markers on the inferred pseudotime trajectory of CAF activation states within the pan-cancer single cell CAF Atlas [12] revealed their enriched expression at early timepoints corresponding to cell activation state 1, which primarily comprised normal and tumor-adjacent fibroblasts and expressed canonical apCAF and iCAF markers (Fig. 1K, Supplemental Fig. S4). iCAF typically exhibit more tissue-specific differences than myCAF, which are highly similar across different tissues [16]. Indeed, C1 displayed several prostate-related characteristics, including *AR* expression and upregulation of steroid hormone metabolism and androgen response pathways (Fig. 1D, F-H, Supplemental Fig. S3B).

C2 and C3 both expressed high levels of CAF-associated activation markers (Fig. 1D, Supplemental Fig. S3C-D). C2 however exhibited abundant expression of iCAF-associated markers and was enriched for the iCAF-associated pathways complement activation, IFNγ/IFNα/TNFα pathways as well as signaling via their downstream mediators NFκB and JAK/STAT (Fig. 1D-H; Supplemental Fig. S3B). Indeed, C2 exhibited similarity to several iCAF subsets distinct to those of C1, including Kieffer IL iCAF [17], Nicolas cluster 3 IL1 TNFa iCAF [15], Chen early PCa CAF [18], and *PDPN*^+^*/SELENOP*^+^*/SMA*^*low*^ CAF-S5 [19, 20] (Fig. 1I-J, Supplemental Fig. S3E). C2 also displayed weak similarity to some early stage myCAF signatures, including Kieffer IFNαβ-myCAF but not late stage TGFβ- or ECM-myCAF [10, 17], suggesting initial transitioning on the myCAF trajectory (Fig. 1I-J, Supplemental Fig. S3E). Supportively and like several canonical iCAF markers, C2 genes were more homogeneously expressed along the CAF Atlas activation trajectory than C1 markers (Fig. 1K, Supplemental Fig. S4).

C3 on the other hand displayed significant upregulation of canonical myCAF genes *ITGA11, SPP1, COL8A1, FN1* and *THBS1* and was enriched for hallmark myCAF pathways, such as ECM organization, epithelial-mesenchymal transition (EMT), collagen biosynthesis, cell adhesion mediated by integrin, glycolysis, hypoxia, TGFβ and VEGF pathways (Fig. 1D-H; Supplemental Fig. S3B) in keeping with acquisition of contractile/myofibroblastic properties. Consistently, C3 demonstrated strong similarity to myCAF signatures from diverse cancer types, including breast, prostate, pancreas and lung cancers (Fig. 1I-J) and were enriched at later timepoints of the CAF Atlas trajectory similar to canonical myCAF markers, particularly at the terminal arm of cell activation state 2 (Fig. 1K, Supplemental Fig. S4). In summary, the fibroblast biobank encompasses distinct cultures that display extensive molecular parallels to early (C1), intermediate (C2) and late (C3) fibroblast activation states that have been identified in situ across multiple cancer types.

### Multimodal profiling validates the presence of multiple fibroblast substates within the biobank

**Fig. 2:**
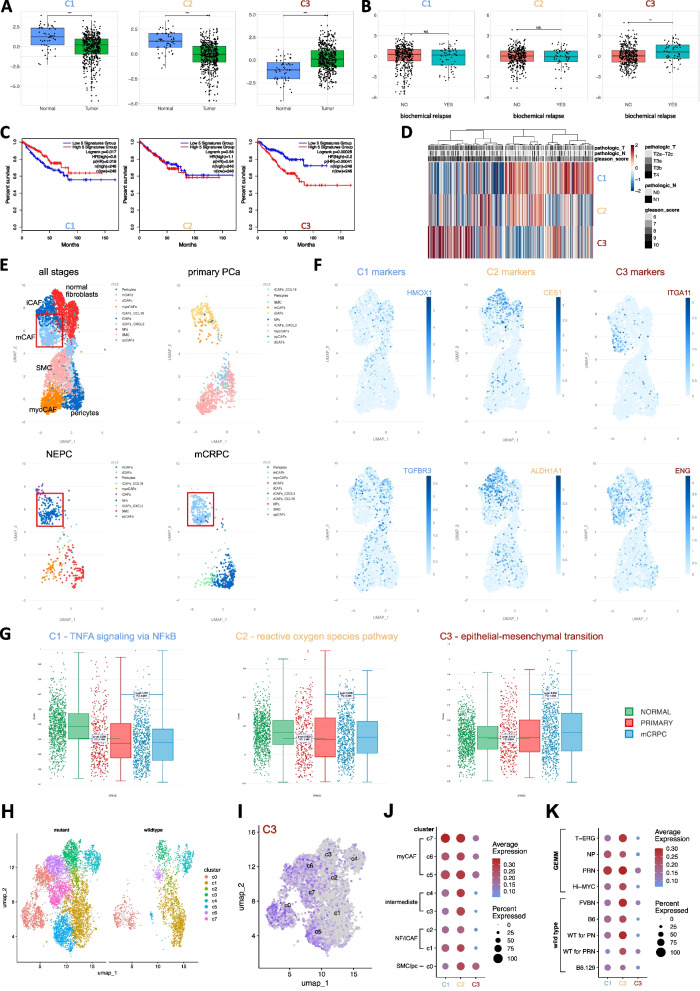
The C3/myCAF state is associated with aggressive PCa. **A**-**D** Expression of cluster-specific signature genes (combined z-score) for each primary fibroblast cluster in the TCGA-PRAD cohort stratified for **A** expression in normal vs. tumor samples or **B** biochemical relapse. **C** Kaplan–Meier plots using substate-delineating genes foremost annotated to the fibroblast lineage in a PCa scRNA-seq dataset [26] (Supplemental Fig. S3G) depicting DFS for each signature using median as group cut-off. **D** Heatmap showing combined z-scores of cluster-specific signature genes across all primary tumor samples in the TCGA-PRAD cohort. **E**–**F ***P*.adj.). **G** Bar plots depicting expression scores of the most highly (padj.) upregulated HALLMARK pathway for each biobank fibroblast subpopulation in cells annotated to the fibroblast lineage in the Prostate Cancer Atlas [28]. **H** UMAP visualization of mesenchymal cell clusters from the Pakula et al. study [29] of different PCa GEMM according to wildtype or mutant status. **I** UMAP projection and **J**-**K** dotplots of UCell scores for murine orthologs to the top five upregulated genes (according to *P.*adj value with log2FC > 0.5) in C1-C3 explant cultures on mesenchymal cells of the Pakula GEMM dataset and stratified per **J** Pakula cluster with phenotypic annotations per the original study [29] or **K** GEMM model. Source data for panels **A**-**D** are provided in the Source Data file

The presence of distinct fibroblast substates within our biobank was validated via quantitative real time PCR (qRT-PCR) of fibroblast activation and substate-delineating markers in an independent cohort of twenty-nine explant cultures as well as in three cultures from the original bulk transcriptomic dataset (qRT-PCR cohort, Supplemental Fig. S5A). Expression levels of C1-C3 markers displayed longitudinal stability within an individual culture (Supplemental Fig. S5B). Immunofluorescent staining for the C2 markers PDGFRA and PDPN and C3 marker ITGA11, readily distinguished cultures in line with their expression profile-based classification (Figs. 1A, 2A, C, Supplemental Fig. S5C). We also performed multiparametric flow cytometry (FC) using a panel of seven stromal markers for thirty-six cultures, including ten samples from the qRT-PCR cohort and four samples from the transcriptomic cohort (Supplemental Fig. S6). All samples were positive for FAP reminiscent of the heterogeneous FAP^+^ CAF-S1 superpopulation from breast cancer [17, 21]. Dimensional reduction and unsupervised clustering based on prominently expressed stromal markers initially identified thirteen clusters (Supplemental Fig. S6A-C). Gating for these clusters based on their corresponding cell surface expression levels revealed eight main clusters (Supplemental Fig. S6D-E). Notably, cultures annotated to a particular fibroblast subpopulation in the transcriptomic and qRT-PCR cohorts displayed high concordance with their grouping in the FC dataset with each fibroblast subpopulation demarcated by a distinct gated FC cluster (Supplemental Fig. S6F-H). Early activated fibroblasts (C1) and transitioning CAF (C2) could be differentiated from C3 myCAF via PDPN consistent with multiple studies demonstrating *PDPN* expression in iCAF [19, 20, 22–24]. In turn, PDPN^+^ C1 and C2 cultures were distinguishable via their differential cell surface expression of MCAM. The C3-enriched cluster 1 was demarcated by lower PDPN and PDGFRA expression but moderate expression of MCAM in accordance with previously reported myCAF [11, 25]. Fluorescence-activated cell sorting (FACS) of cultures for clusters 6, 10 or 1 and subsequent qRT-PCR confirmed high expression levels of C1, C2 or C3-delineating markers in cells sorted for FC gated clusters 6, 10 and 1, respectively (Supplemental Fig. S6I). In summary, this orthogonal approach confirmed the biobank comprises multiple distinct and phenotypically stable fibroblast cultures with molecular profiles closely resembling previously reported CAF subpopulations.

### Explant cultures display molecular parallels to distinct CAF substates during PCa progression

Late activation/myCAF phenotypes positively correlate with hallmarks of poor prognosis across multiple tumor types [30], whereas early activation/iCAF phenotypes are frequently associated with positive clinicopathological parameters [16]. Thus, we next investigated whether C1-C3 markers displayed similar associations. Concordant with their inferred trajectories, C1 and C2 marker genes were significantly lower in the TCGA-prostate adenocarcinoma (PRAD) cohort compared to benign controls whereas C3 marker genes were significantly elevated in primary PCa and positively correlated with poor prognostic indicators, including biochemical relapse (Fig. 2A-D; Supplemental Fig. S3F). Moreover, the C3 signature was associated with decreased disease-free survival (DFS; hazard ratio (HR) 2.2; *P* = 4.1 × 10^–4^), which was in stark contrast to the positive association of the C1 signature with DFS (HR 0.6; *P* = 0.018).

To refine these bulk RNA-seq analyses, we scrutinized expression levels of C1-C3 defining markers or pathways in stromal cells or fibroblasts of two independent human PCa single cell atlases encompassing diverse disease stages [27, 28] (Fig. 2E, Supplemental Fig. S7-8). Consistent with data thus far, C1 markers were primarily expressed by normal fibroblasts/iCAF in normal and benign-adjacent samples but were largely absent from tumor samples (Fig. 2E, Supplemental Fig. S7). Similarly, the highest-scoring C1 HALLMARK pathway “TNFA_signaling_via_NFKB” and C1 marker genes were abundant in normal prostate tissues but significantly decreased in primary PCa and metastatic castration-resistant PCa (mCRPC; Fig. 2G, Supplemental Fig. S8A). In contrast, C2 markers were expressed in iCAF, which were enriched in benign-adjacent and primary PCa tissues but largely absent from the neuroendocrine PCa (NEPC) and mCRPC samples (Fig. 2E, Supplemental Fig. S7-8). Consistent with their shared upregulation of HALLMARK pathways related to ECM and EMT, C3 markers were primarily expressed in “matrix-CAF” (mCAF), a cluster particularly enriched in NEPC and mCRPC samples. Likewise, the gene signature of the C3 HALLMARK pathway "epithelial_mesenchymal_transition" and C3 markers were significantly upregulated in primary PCa and CRPC samples (Fig. 2E, Supplemental Fig. S7-8).

Since the TME in genetically-engineered mouse models (GEMM) displays remarkable cross-species conservation to the TME of the corresponding human cancer type [22, 29], we also analyzed murine orthologs to C1-C3 markers in a single cell atlas of PCa GEMM with differing aggressiveness [29] (Fig. 2H-K, Supplemental Fig. S9). C1 orthologs were homogeneously distributed across wildtype and mutant fibroblast clusters from different GEMM, whereby the orthologous C2 signature was more abundantly expressed than C1 genes in clusters 3 and 4, which were prevalent in the less aggressive *T-ERG, Hi-MYC* and *NP* models and expressed Wnt signaling components consistent with an intermediate/proto-myCAF state [29, 31] (Fig. 2H-K, Supplemental Fig. S9A-F). Moreover, C1 and C2 markers were expressed at early and intermediate timepoints of the inferred pseudotime fibroblast activation trajectory, respectively (Supplemental Fig. S9I-J). Besides the SMC/pericyte cluster c0, expression of the orthologous C3 gene signature in the mouse PCa GEMM atlas was largely restricted to the ECM/collagen-rich CAF clusters c5-c7, which were highly enriched in the aggressive PRN model that develops NEPC [29] with C3 markers also expressed at markedly later timepoints of the myCAF activation trajectory (Fig. 2H-K, Supplemental Fig. S9I-J).

**Fig. 3:**
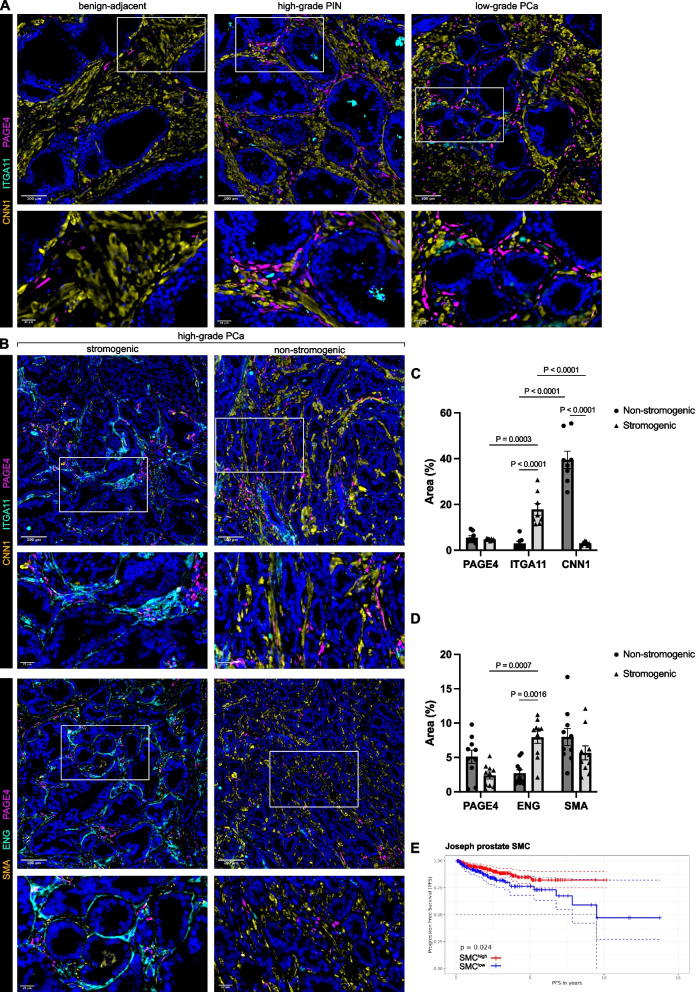
CAF subpopulations are dynamic during prostate cancer progression. **A**-**B** Human prostate tissue sections of indicated pathology were stained using the antibodies shown. Font color denotes pseudo-coloring in the displayed merged images. Nuclei were counterstained using Hoechst 33342. Boxed regions are shown enlarged beneath each parental image. Images are representative of 4 independent experiments using tissue sections from 8 different patients. **C**-**D** ImageJ quantification of tissues stained as in **B** whereby the area stained by the indicated antibody is expressed as a percentage of the total DAPI^+^ area using fields of view of constant size and with constant threshold settings. Data represent mean ± SEM of at least eight fields of view of constant size from three different high-grade tumors. **E** Kaplan–Meier plots depicting progression-free survival (PFS) of high-grade (≥ T stage T2c) samples of the TCGA-PRAD cohort stratified as described in Materials and Methods for high (SMC^high^) or low (SMC^low^) expression levels of the indicated SMC signatures. Source data for panel **E** is provided in the Source Data file

To further validate key C1-C3 delineating markers in clinical PCa at the protein level and provide spatial context, patient tissues were immunostained for the SMC markers CNN1, CCDC102B or SMA (encoded by *ACTA2*), the C1/C2 marker PAGE4, C2 marker CES1 and C3 markers ITGA11 or ENG (Fig. 3, Supplemental Fig. S10). ITGA11, CES1 and PAGE4 were sparsely expressed in benign-adjacent tissues, which was predominated by SMC (Fig. 3A, Supplemental Fig. S10A-C) with expression of the TGFβ co-receptor ENG restricted to the endothelium of some vessels as previously reported [32]. PAGE4 and CES1 however were markedly upregulated high-grade PIN (HGPIN) and low-grade PCa being localized to both the periglandular and interstitial stroma. In contrast, expression of both ENG and the ECM-binding cell surface adhesion receptor ITGA11 remained low in HGPIN but an increased frequency of cells expressing low/moderate levels of ITGA11 or ENG was observed in low-grade PCa. Across each of these stages, SMC remained the predominant parenchymal stromal cell type.

Two distinct types of high-grade tumors were identified: those with a considerable proportion of parenchymal SMC yet low numbers of ITGA11^+^/ENG^+^ CAF *vs.* tumors with a near complete loss of parenchymal immunoreactivity for the mature SMC differentiation marker CNN1 and lower levels of the general SMC marker SMA but a high abundance of ITGA11^+^/ENG^+^ cells that were predominantly restricted to the peri-tumoral space (Fig. 3B-D). These distinct stromal compositions are consistent with previously described “non-stromogenic” and “stromogenic” PCa, respectively [3]. Whilst the abundance of PAGE4^+^ or CES1^+^ cells did not significantly differ between these two tumor types, PAGE4^+^/CES1^+^ cells tended to accumulate in nests within intraglandular areas of stromogenic tumors. This pattern was not readily apparent in non-stromogenic high-grade tumors (Fig. 3B, Supplemental Fig. S10A) suggesting the presence of defined spatial CAF niches in stromogenic advanced PCa. In further support of the clinical relevance of ITGA11^+^/ENG^+^ stromogenic *vs.* non-stromogenic tumors, we observed that progression-free survival (PFS) of patients with pathological tumor stage ≥ T2c was significantly higher for those with tumors expressing high levels of SMC markers compared to those with lower expression of SMC markers (Fig. 3E, Supplemental Fig. S10H-I). In summary, PCa progression is associated with dynamic changes in CAF subpopulations whereby the C3 markers ENG and ITGA11 demarcate the predominant CAF substate in stromogenic high-grade tumors with potential implications for improved prognostic staging.

### CAF subpopulations display distinct phenotypic hallmarks

**Fig. 4:**
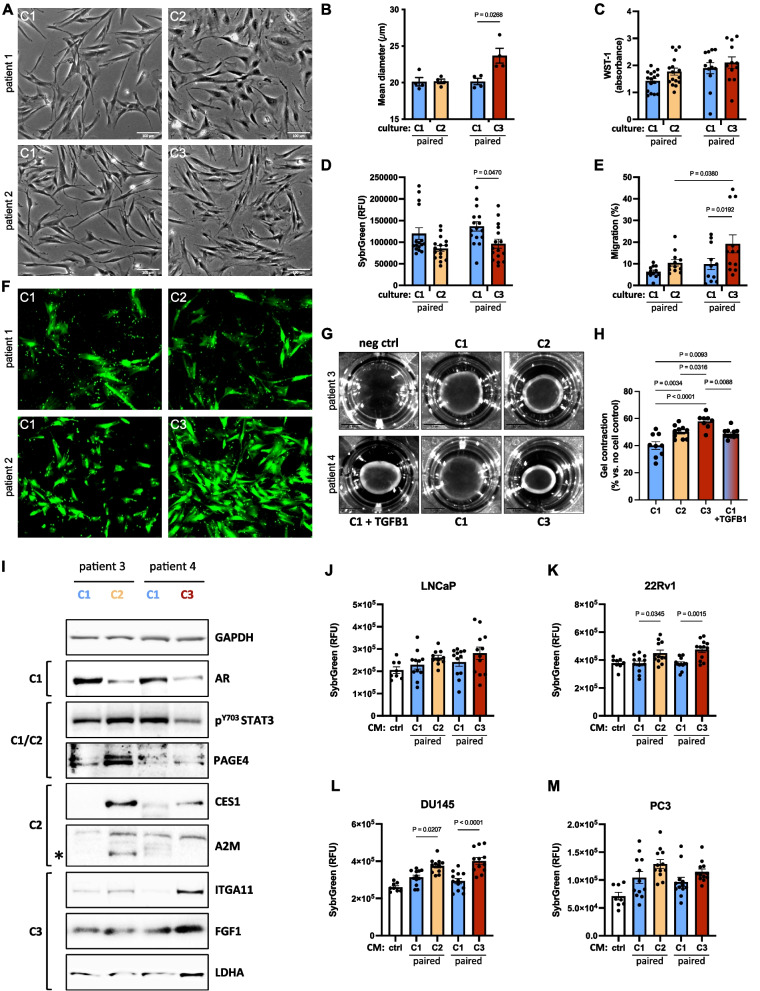
Primary CAF cultures exhibit distinct functional hallmarks. Explant cultures displaying a C2 or C3 molecular profile and patient-matched C1 cultures were characterized via **A** brightfield imaging, **B** cell size, **C** cell viability via WST-1 assay, **D **proliferation via SybrGreen staining, **E**–**F** transwell migration, **G**-**H** collagen I gel contractility and **I **Western blotting with the antibodies indicated (* denotes the A2M band). **J**-**M** Proliferation of **J**-**K **AR^+^ or **L**-**M **AR^−^ PCa cell lines incubated with CM from C2 or C3 cultures and patient-matched C1s. Non-conditioned media incubated for the same duration without primary fibroblasts served as control (ctrl). Data represent mean fluorescence of quadruplicate wells from independent experiments using fibroblasts derived from three different donors. Statistical significance was calculated: **B**-**E** two-way ANOVA with Holm-Šídák multiple comparison correction, **H**, **K **one-way ANOVA with Tukey multiple comparison correction. **A**, **F**, **G**, **I** Images are representative of at least three different experiments using primary cells isolated from different donors

A paucity of in vitro models has limited our understanding of CAF heterogeneity at the functional level. We therefore employed C1 and patient-matched C2 or C3 explant cultures representing early activated (C1), intermediate (C2) and late activation (C3) fibroblast states from approximately thirty patients for an initial functional characterization. C1 cultures displayed a typical spindle-like, light-refractive fibroblast morphology, whereas both C2 and C3 cultures exhibited greater 2-dimensional (2D) spreading with suspensions of C3 cells slightly but significantly larger than C2 and patient-matched C1 cells (Fig. 4A-B). No differences were observed in cell viability. However, C2 and C3 explant cultures proliferated slower than patient-matched C1 (Fig. 4C-D). Consistent with their myCAF annotation and expression of ITGA11, a TGFβ-responsive collagen type I-binding integrin that promotes CAF migration [33], C3 cultures displayed significantly higher migration and collagen-1 gel contraction than either C2 or patient-matched C1 (Fig. 4E-H).

We next sought to validate at the protein level key components of pathways enriched in each of the culture subpopulations (Fig. 4I). C2 displayed the strongest expression of carboxylesterase 1 (CES1), an NFκB-regulated alternatively spliced serine hydrolase involved in lipid/cholesterol metabolism [34], a finding consistent with their upregulation of adipogenesis (Fig. 1E, Supplemental Table 9). Likewise, C2 cultures expressed prostate-associated gene 4 (PAGE4), a highly intrinsically disordered stress-response protein implicated in attenuating PCa cell invasion, epithelial AR activity and canonical Wnt/β-catenin signaling [35–37], and alpha-2-macroglobulin (A2M), which acts as an intra- and extra-cellular chaperone of inflammatory cytokines, including IL1β, and activates the complement cascade [38], an iCAF hallmark pathway that was also upregulated in C2 cultures (Fig. 1G). Pro-inflammatory cytokines induce iCAF formation at least in part via JAK/STAT signaling [39]. In silico analyses implicated TNFα and interferons gamma and alpha and their downstream signaling mediator STAT3 in C2 cultures (Fig. 1E, H, Supplemental Table 9; Supplemental Fig. S3B). Accordingly, C1 and C2 cultures displayed abundant levels of STAT3 phosphorylated at tyrosine 705 (pSTAT3) compared to C3 cultures (Fig. 4I). Consistent with enrichment of steroid hormone metabolism/signaling (Fig. 1F-H), elevated AR expression in C1 cultures was also confirmed at the protein level. C3 cultures on the other hand exhibited low levels of AR but high levels of FGF1 and ITGA11 (Fig. 4I). The “reverse Warburg effect” is a key myCAF hallmark [40]. Accordingly, C3 cultures displayed the highest levels of LDHA, indicating a strong glycolytic activity. Supportively, the lactate transporter *MCT4* and *HIF1A*, which are involved in promoting glycolysis and myofibroblast differentiation [40], were significantly upregulated (Supplemental Table 3), suggesting C3 cultures may be highly active in supporting cancer cell metabolism and growth.

Pro-inflammatory and mitogenic growth factors secreted by CAF can either support or restrict cancer growth [1]. We therefore assessed proliferation of PCa cells following exposure to conditioned media (CM) from C1, C2 or C3 cultures or non-conditioned media as control. When exposed to CM from C1 cultures, PCa cells proliferated at similar rates to control media, regardless of their AR status, indicating that C1 cultures do not significantly influence cancer cell growth. Conversely, CM from C3 cultures, which are linked to poor clinical outcomes, significantly increased the proliferation of both AR-positive and AR-negative PCa cell lines (Fig. 4J) suggesting that C3 cultures promote cancer cell growth. Similar but less pronounced effects were observed for CM from C2 cultures.

**Fig. 5:**
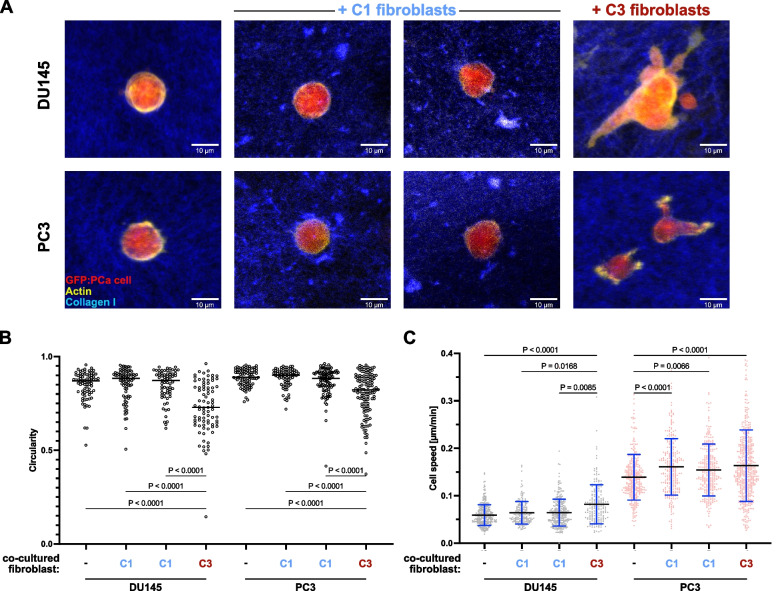
Myofibroblastic CAF promote prostate cancer cell invasion in 3D collagen matrices. **A**-**C** PCa cell lines stably overexpressing GFP were embedded with or without explant cultures of the indicated substate in a 3D composite network of fluorescent collagen fibers. **A** Visualization of maximum-intensity projections of embedded GFP^+^ PCa cells following actin immunostaining using phalloidin and confocal imaging. Representative pseudocolored overlays of GFP^+^ PCa cells (red), actin (yellow) and collagen I (blue) are shown. Scale bars represent 10 µm. **B** Morphology analysis of PCa cells within the 3D collagen network. GFP^+^ PCa cells were manually segmented and circularity quantified using FIJI. Data represent three independent biological replicates using primary fibroblasts isolated from different donors. **C** Migration of PCa cells embedded in a 3D composite network of fluorescent collagen fibers imaged for 8 h at a rate of one image every 10 min using a spinning disk microscope. Image stacks spanning 140 µm were acquired, and maximum-intensity projections generated. Fluorescent cells were quantified using the FIJI plugin TrackMate. Mean cell speed was calculated from tracks obtained in three biological replicates using primary fibroblasts isolated from different donors. **B**-**C** Statistical significance was calculated via one-way ANOVA with Tukey’s multiple comparison test

Together with their ECM remodeling capacity, CAF deposit tracks to which tumor cells adhere thereby promoting cancer cell invasion and migration [41, 42]. In view of the elevated motility of C3 cultures, we investigated whether C1 or C3 cultures differed in their ability to promote PCa cell migration in 3D composite networks of collagen (Fig. 5). In the absence of fibroblasts, DU145 and PC3 cells displayed high circularity in 3D collagen networks indicative of a low motile state, which remained unaltered upon direct co-culture with C1 fibroblasts (Fig. 5A-B). Upon co-culture with C3 fibroblasts however, PC3 and DU145 cells displayed significantly lower circularity with readily apparent protrusions suggestive of active migration (Fig. 5A-B). Indeed, real time imaging revealed that C3 fibroblasts significantly increased DU145 cell migration in 3D collagen networks, a finding in stark contrast to their stationary behavior when cultured alone or with C1 fibroblasts (Fig. 5C and Supplemental Movies 1 and 2). Similar but less pronounced effects were observed for PC3 as well as LNCaP and 22Rv1 cells (Fig. 5C and data not shown), whereby it may be noted that PC3 cells secrete active TGFβ1 and 2 and induce phenotypic switching of C1 cultures to a C3-/myCAF-like state in a manner dependent on fibroblast TGFβR activity [43].

Collectively, these studies demonstrate that the different fibroblast substates are not only molecularly divergent but also differentially influence cancer cell growth and invasion—some supporting these onco-supportive properties more than others, and further implicate the late/myCAF phenotype in promoting aggressive PCa.

### AR is downregulated in myofibroblastic CAF

**Fig. 6:**
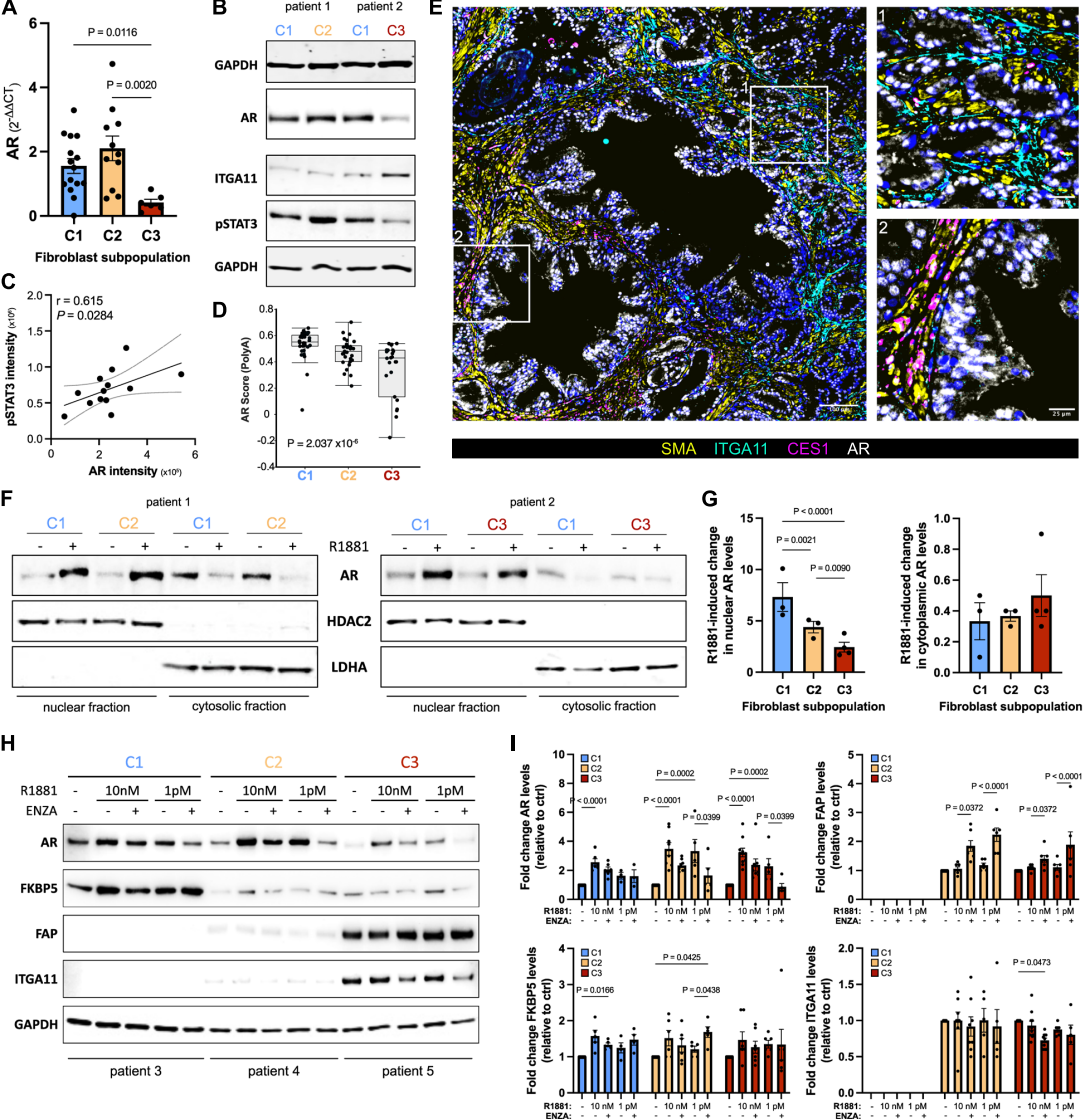
Myofibroblastic CAF display altered sensitivity to AR signaling modulation. AR expression levels via **A** qRT-PCR in the independent explant cohort and **B** Western blotting. **C** Densitometric-based correlation of AR and pSTAT3 protein levels in thirteen explant cultures. **D** AR score of samples from the SU2C metastatic PCa cohort grouped according to expression of fibroblast substate-delineating markers. **E** Immunofluorescent staining of a human prostate tissue section comprising prostate cancer (boxed region 1) and benign-adjacent tissue (boxed region 2) using the antibodies indicated. Font color denotes pseudo-coloring in the displayed merged images. Nuclei were counterstained using Hoechst 33342. Boxed regions are shown enlarged, right. **F** Western blotting and **G** densitometric quantification of AR in nuclear and cytosolic extracts incubated with or without 10 nM R1881 for 1 h. **H** Western blotting and **I **densitometric quantification of the antibodies indicated in fibroblast cultures pretreated in hormone-depleted medium with 10 µM enzalutamide (ENZA) or vehicle equivalent before addition of 10 nM or 1 pM R1881 for a further 72 h. Values denote normalized fold change in protein levels relative to the corresponding control. Statistical significance was calculated: **A**-**B**, **G** one-way ANOVA with two-stage step-up method of Benjamini, Krieger and Yekutieli multiple comparison correction; **C **Pearson correlation; **D **Kruskal–Wallis test; **I **two-way ANOVA with Tukey’s multiple comparison test. **B**, **E**–**F**, **H** Images are representative of at least three independent experiments using primary material from different donors

Loss of stromal AR in PCa is an established but poorly understood indicator of poor prognosis [44]. Since C1 cultures expressed higher levels of AR than C3 cultures and were enriched for GO terms/hallmark pathways related to androgen response and metabolism (Fig. 1D, F-H; Supplemental Fig. S3B), subsequent studies focused on exploiting our biobank to investigate endogenous AR function in primary prostate fibroblasts in the context of PCa. First, we confirmed elevated expression of *AR* in C1 and its downregulation in C3 cultures via qRT-PCR in samples remaining from the independent cohort as well as at the protein level in additional biobank cultures (Fig. 6A-B, Supplemental Fig. S11A). AR protein levels exhibited a non-significant trend towards inverse correlation with the C3 marker ITGA11 but significantly positively correlated with pSTAT3 levels (Fig. 6B-C, Supplemental Fig. S11A-B), a hallmark of C1/C2 cultures (Fig. 1H) and key pathway of the iCAF/early CAF phenotype [45].

As previously reported, stromal AR was primarily expressed in parenchymal SMC of the benign prostate but decreased in PCa (Supplemental Fig. S11C). We noted however nuclear AR expression in stromal cells negative for SMC markers that were interspersed between tumorigenic glands, strongly reminiscent of the spatial distribution of PAGE4^+^/CES1^+^ CAF (Fig. 3, Supplemental Fig. S11C). Indeed, multiplex staining revealed co-expression of nuclear AR with the early/intermediate C1-C2 markers CES1 and PAGE4 (Fig. 6E, Supplemental Fig. S11D-F). In contrast and consistent with the above in vitro findings, AR was expressed at significantly lower levels in ITGA11^+^/ENG^+^ myCAF in clinical PCa and was significantly decreased in samples of the SU2C metastatic cohort exhibiting a C3 profile (Fig. 6D-E, Supplemental Fig. S11D-F). These data raised the possibility that by virtue of their differential AR expression, distinct fibroblast substates may be differentially sensitive to androgens and ADT/ARSI, such as enzalutamide.

### AR downregulation in myCAF is associated with insensitivity to the anti-proliferative effects of enzalutamide

**Fig. 7:**
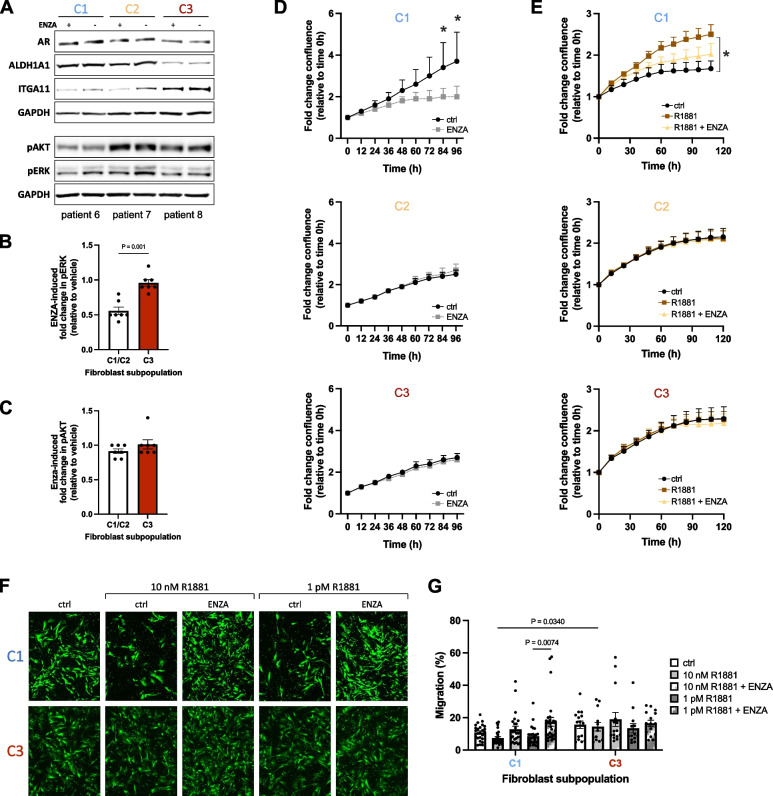
AR loss in myofibroblastic CAF is associated with proliferative- and migratory-insensitivity to enzalutamide. **A** Western blotting using the antibodies indicated and **B**-**C** densitometric quantification of fibroblast cultures incubated under hormone-replete conditions for 72 h with 10 µM enzalutamide relative to the corresponding vehicle treated culture. **D**-**E** Real time proliferation of the indicated fibroblast culture incubated **D** with 10 µM enzalutamide (ENZA) or **E** 1 pM R1881 with 10 µM enzalutamide or vehicle equivalent (ctrl, control). **F**-**G** Migration assay of the indicated fibroblast culture pretreated with 1 pM or 10 nM R1881 and 10 µM enzalutamide or vehicle equivalent (ctrl, control) for 96 h. **G** Values denote percent migration whereby cells seeded to wells without transwell inserts were set to 100%. Statistical significance was calculated: **B**-**C** Mann–Whitney test; **D**-**E**, **G** two-way ANOVA with Holm-Šídák multiple comparison correction. **A**, **F** Images are representative of at least three independent experiments using primary material from different donors

We thus next sought to assess AR functionality among the distinct fibroblast substates encompassed within our biobank (Figs. 6F-I and 7). Despite different total AR levels (Fig. 6A), the synthetic androgen R1881 rapidly induced AR nuclear translocation in cells pre-incubated under steroid hormone-depleted conditions, regardless of the fibroblast substate (Fig. 6F). In C3 cultures however, ligand-induced nuclear translocation was significantly lower than that induced in C1 or C2 cultures (Fig. 6G). Concordantly, the corresponding decrease in cytosolic AR levels in response to R1881 was markedly attenuated in C3 cultures compared to C1/C2 (Fig. 6G). Target genes modulated by endogenous stromal AR remain poorly defined. Across all cultures however, R1881 significantly increased AR levels concordant with ligand-mediated AR stabilization [46] in a manner attenuated by enzalutamide and modestly increased protein levels of the positively regulated AR target FKBP5. Notably and in line with previous reports, enzalutamide significantly increased protein levels of the CAF activation marker and negative AR target FAP in both C2 and C3 cultures [7] whereby the effect in C3 cultures was markedly lower compared to C2 cultures, potentially reflecting their lower AR abundance (Fig. 6H). Except for a modest but consistent decrease in ITGA11 protein levels in C3 cultures that was further exacerbated by enzalutamide, no discernible change was observed in the expression of fibroblast substate markers either at the mRNA or protein level in response to picomolar or nanomolar concentrations of exogenous androgen or upon enzalutamide-mediated AR inhibition (Figs. 6H-I, 7 A and data not shown).

AR also engages in crosstalk with intracellular signaling pathways. Indeed, whilst enzalutamide treatment under steroid hormone-replete conditions did not alter phospho-AKT levels, AR inhibition significantly decreased phospho-levels of ERK1/2 in C1 and C2 fibroblasts but not in C3 cultures (Fig. 7A-C). Furthermore, enzalutamide significantly attenuated the proliferation of C1 cultures, an effect that was not observed in C2 or C3 cultures potentially due to high basal pAKT activity (Fig. 7A, C-D, Supplemental Fig. S11G), a key regulator of CAF proliferation [47]. Similarly, under steroid hormone-depleted conditions exogenous androgen promoted the proliferation but suppressed the migration of C1 cultures in an AR-dependent manner, whereas this effect was not observed in either C2 or C3 cultures (Fig. 7E-G, Supplemental Fig. S11H-I and data not shown). Collectively, these data indicate that variability in AR levels among the fibroblast substates translates into functional differences with regards to their proliferative and migratory sensitivity to modulation of AR signaling.

### The myCAF phenotype is associated with castration-resistance in vivo 

**Fig. 8:**
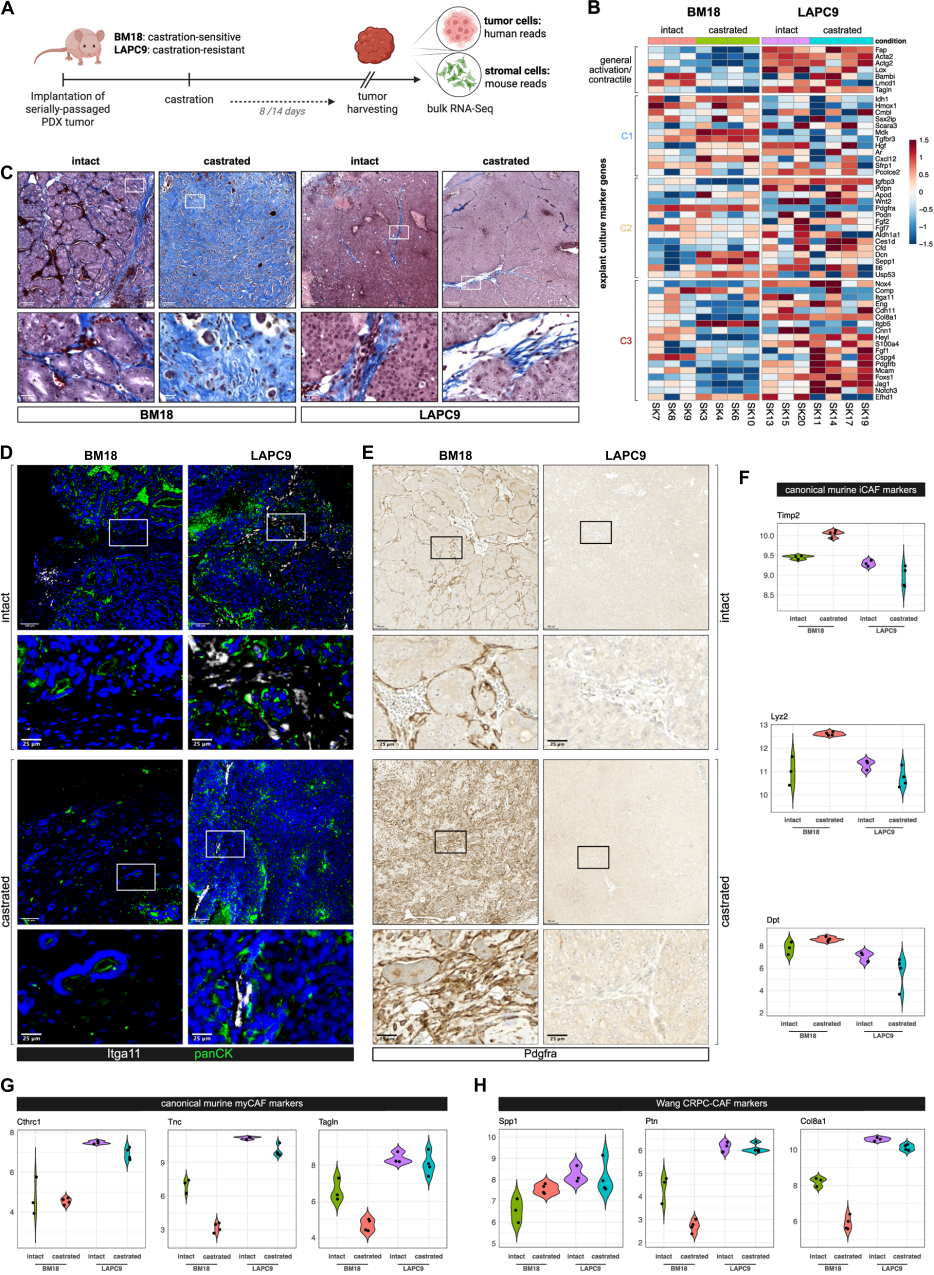
Myofibroblastic CAF are associated with castration-resistance in vivo.*.***A** Graphical overview of the PDX system whereby serially passaged tumors were subcutaneously implanted into nude mice. Once tumors established, hosts were left intact or underwent castration and tumors harvested 8 (LAPC9) or 14 (BM18) days later. Since host stromal cells infiltrate human PCa PDX tissue and eventually replace human stromal tissue after serial passaging [50, 51], the stromal and tumoral transcriptomes of harvested tumors could be distinguished in bulk RNA-sequencing data as mouse- or human-specific reads, respectively. **B** Sample level heatmap of murine orthologs of the indicated C1-C3 markers in intact or castrated mice harboring the indicated PDX tumor. **C** Masson’s trichrome, **D** immunofluorescent and **E** immunohistochemical staining of the indicated PDX tumors (*n* = 3 tumors stained in four independent experiments). Boxed regions are shown enlarged beneath each parental image*. ***D** Font color denotes pseudo-coloring in the displayed merged images. Nuclei were counterstained using Hoechst 33342. **F**–**H** Violin plots depicting expression of canonical murine PCa **F **iCAF, **G **myCAF markers [29] or **H **CRPC-CAF markers [52] in the indicated PDX model. **A** Adapted from [50]

In silico and functional data thus far implicated a potential association of AR^low^ myCAF with aggressive castration-resistant disease. We therefore investigated CAF heterogeneity in two well-established metastatic PCa (mPCa) PDX models whereby the BM18 model is androgen-sensitive and regresses upon host castration thereby mimicking hormone-sensitive PCa [48]. This contrasts with the castration-insensitive LAPC9 PCa PDX model [49], which exhibits continued exponential growth under castrate conditions and thereby mimics the androgen-independent state (Fig. 8A) [50]. Stromal expression levels of mouse orthologs to C1-C3 markers were discerned by exploiting reports that infiltrating host stromal cells replace human-derived stroma in serially passaged subcutaneously-maintained PDXs [51], as confirmed in our previous study of the BM18/LAPC9 models [50]. Whilst general activation and C3 markers were expressed in the intact BM18 model, their expression levels were markedly higher in intact LACP9 xenografts (Fig. 8B, Supplemental Fig. S12A), an observation consistent with the more aggressive phenotype of the LAPC9 model and enrichment of C3 markers in advanced PCa (Fig. 2, Supplemental Fig. S7-9). Rather, intact BM18 tumors expressed higher levels of C1 markers than intact LAPC9 tumors (Fig. 8B, Supplemental Fig. S12A). These findings were confirmed at the protein level for Pdgfra and Itga11 (Fig. 8D-E, Supplemental Fig. S12B-E). Accordingly, an abundance of Itga11^+^ cells was detected throughout the intra-tumoral stroma of intact LAPC9 xenografts. Conversely, Itga11 immunoreactivity was restricted to the periphery of intact BM18 tumors with no Itga11^+^ cells detectable in the intra-tumoral stroma (Fig. 8D, Supplemental Fig. S12D).

Notably, upon castration C3 markers were further upregulated in the LAPC9 stroma (Fig. 8B, Supplemental Fig. S12A). Due to the continued exponential tumor cell growth of this castration-resistant model, only a limited amount of stromal tissue remained in the tumors harvested at experimental endpoint. Nonetheless, intra-tumoral Itga11^+^ cells were readily detectable at both the tumor periphery and within the tumor core (Fig. 8D, Supplemental Fig. S12B-D), indicating their insensitivity to androgen deprivation. In contrast, castration-induced regression of BM18 tumors was accompanied by decreased expression of 14 out of 16 C3 markers and absence of Itga11 immunoreactivity but a concomitant increase in C1 and some C2 markers (Fig. 8B, Supplemental Fig. S12D-E), implying in vivo plasticity of these fibroblast subpopulations in response to castration, mimicking first-line ADT.

Consistent with higher expression of murine orthologs to C1 or C3 markers in the BM18 and LAPC9 models, respectively, the BM18 and LAPC9 stromal compartments also displayed differential upregulation of canonical murine iCAF and myCAF markers (Fig. 8F-G). Similarly, the Pakula intermediate cluster C4 that prevailed in wildtype B6 and Hi-MYC mice [29] was strongly represented in the BM18 stroma whereas the LAPC9 stroma highly expressed C5 markers, an ECM/collagen-rich cluster enriched in the aggressive PRN model (Supplemental Fig. S12F, S14I). Moreover, both the intact and castrated LAPC9 stroma exhibited significant upregulation of genes that demarcated the Spp1^+ ^myCAF/CRPC-CAF cluster 5 that was specifically enriched in the aggressive *Pten*^PC−/−^; *Trp53*^PC−/−^ PCa model following ADT (Fig. 8H, Supplemental Fig. S12G) and whose targeted deletion greatly extended lifespan of the double knockout mice [52]. Thus, the host response and stromal composition of the BM18 and LAPC9 tumors bear numerous molecular similarities to multiple PCa GEMM models.

In summary, the molecular profiles of the fibroblast substates within our biobank exhibit striking parallels to the stromal compartment in vivo*.* Potentiation of the C3-/late myCAF-like substate upon surgical (PDX) or chemical (*Pten*^PC−/−^; *Trp53*^PC−/−^ PCa model) castration in conjunction with the insensitivity of C3 cell proliferation/migration to AR modulation further implicate the C3/late myCAF substate in CRPC. Moreover, the data imply that androgen depletion may favor survival of the clinically unfavorable AR^low^ myCAF state. Exploring the signaling pathways that drive the C3 substate may thus provide actionable targets for therapy.

### TGFβ signaling promotes AR loss in prostate fibroblasts

**Fig. 9:**
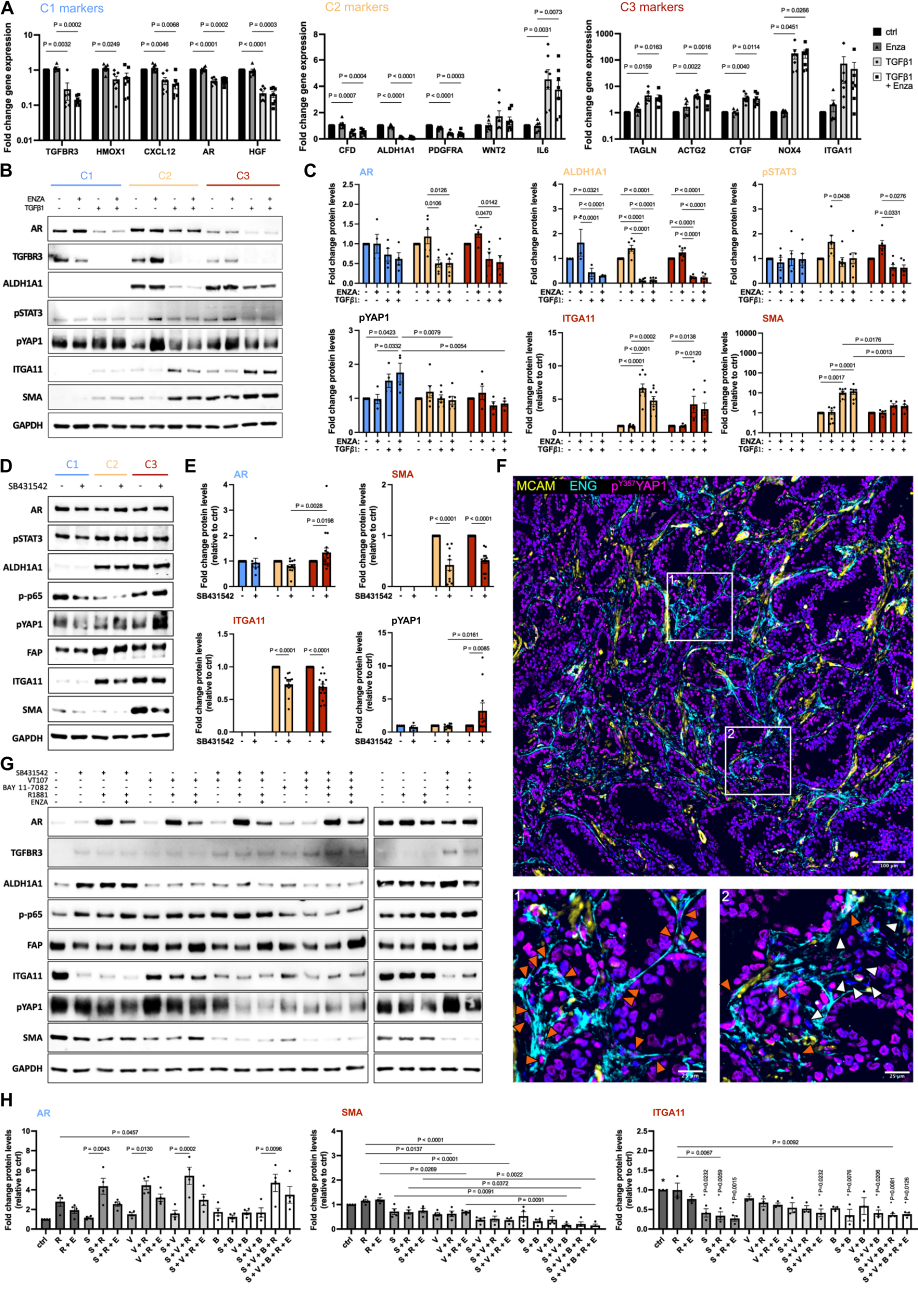
Signaling by an NFκB-YAP1-TGFβ axis underlies AR loss during fibroblast phenotypic switching. **A** qRT-PCR of C1 cultures treated with 10 µM enzalutamide (ENZA) or vehicle control in the presence or absence of 2 ng/ml TGFβ1 for 72 h. **B** Western blotting with **C** densitometric quantification of the indicated cultures treated as in **A**. **D**, **G** Western blotting with **E**, **H** densitometric quantification of fibroblast cultures treated as indicated with 1 µM SB431542, 10 nM R1881, 100 nM VT107, 2 µM BAY11-7082, 10 µM ENZA or vehicle equivalent for 96 h under steroid hormone **D**-**E** -replete or **G**-**H** -depleted conditions. **F** Immunofluorescent staining of human prostate cancer tissue using the antibodies indicated. Font color denotes pseudo-coloring in the displayed merged images. Nuclei were counterstained using Hoechst 33342. Boxed regions are shown enlarged below. Orange arrowheads (panels 1 and 2) highlight myCAF nuclei positive for p^Y357^YAP1, white arrowheads (panel 2) demarcate MCAM^−^ENG^−^ stromal cells expressing low/non-detectable levels of p.^Y357^YAP1. Statistical significance was calculated: **A**, **C**, **E** two-way ANOVA with **A** two-stage step-up method of Benjamini, Krieger and Yekutieli or **C**, **E** with Tukey’s multiple comparison test and **H** one-way ANOVA with Tukey’s multiple comparison test. **B**, **D**, **F**-**G** Images are representative of at least three independent experiments using primary material from different donors. **H** Abbreviations denote B, BAY 11–7082; E, enzalutamide; R, R1881; S, SB431542; V, VT107. * P values above bars denote statistically significant comparisons relative to control treated cells, demarcated *. **C**, **E** Protein levels of C3 markers ITGA11 and SMA could not be reliably quantified in C1 cultures due to their basal low expression. **C**, **E**, **H** For improved comprehension font color indicates the fibroblast substate primarily delineated by the respective marker (blue, C1; yellow, C2; red, C3; black, not substate-specific)

In view of the abovementioned findings, we next sought to delineate intracellular signaling pathways that determine CAF substate identity and drive AR loss. In line with their myCAF phenotype and expression of the TGFβ co-receptor *ENG* [53], PROGENy revealed enrichment of TGFβ signaling in C3 cultures (Fig. 1H). Likewise, the HALLMARK_TGFbeta_signaling pathway was strongly represented in C3 cultures and LAPC9 stroma with TGFβ target genes abundantly expressed in the NEPC and mCRPC-enriched myCAF clusters of the PCA, PCCAT and PCa GEMM single cell atlases (Supplemental Fig. S7-9, S13). Consistently, treatment of C1 cultures with recombinant TGFβ1 in steroid hormone-replete media significantly upregulated C3 markers at the mRNA and protein level, whereby the latter could not be reliably quantified due to their low/non-detectable levels under basal conditions consistent with the low activated profile of these cultures (Fig.9A-C). Such C3 marker upregulation is concordant with our previous observations of cancer cell-induced plasticity of C1 cultures, whereby C1 cultures treated with CM from TGFβ1-/2-secreting PC3 cells significantly upregulated C3/myofibroblast markers in a manner dependent on fibroblast TGFβR activity [43]. Notably, C1 and C2 markers, including AR, were concomitantly downregulated upon TGFβ1 treatment (Fig. 9A-C). Enzalutamide treatment, neither alone nor in combination with TGFβ1, significantly altered C1, C2 or C3 marker expression. Similar findings were observed for C2 and C3 cultures, although C3 cultures exhibited marked attenuated induction of the C3 markers ITGA11 and SMA most likely reflecting their high intrinsic activity of the TGFβ pathway (Fig. 9A-C).

To ascertain whether TGFβ signaling alone determines the fibroblast phenotypic substate, we treated C1-C3 cultures under steroid hormone-replete conditions with the ALK5 inhibitor SB431542. Consistent with their low activation profile, TGFβR inhibition had little effect at either the mRNA or protein level in C1 cultures (Fig. 9D, Supplemental Fig. S14A). While SB431542 markedly decreased SMA levels in C2 and C3 cultures, the effect on ITGA11 levels and other C3 markers at the mRNA level was modest, and residual SMA and ITGA11 levels in C3 cultures remained abundant suggesting that pathways in addition to TGFβ are required to maintain the C3 phenotype. Notably, AR protein levels were slightly but significantly increased in C3 cultures following TGFβR inhibition (Fig. 9E) further implicating this pathway in contributing to stromal AR loss.

Since stromal TGFβ and AR signaling axes engage in suppressive crosstalk [54], we examined whether R1881 synergizes with TGFβR inhibition to modulate the C3 phenotype. Indeed, in addition to the ligand-dependent AR protein stabilization (Fig. 6H), exogenous R1881 under otherwise steroid hormone-depleted conditions further decreased ITGA11 but not SMA protein levels compared to either agent alone in a manner intriguingly potentiated by enzalutamide (Fig. 6I, lanes 1–4 Fig. 9G and H).

### Combined targeting of YAP1-TGFβ-NFκB signaling axes enhances abrogation of the myCAF state

Besides TGFβ signaling, the mechanotransducer Yes-associated protein 1 (YAP1) is also implicated in driving myCAF conversion [55]. While all three fibroblast substates expressed comparable basal levels of YAP1 phosphorylated at the activating residue tyrosine 357 [56] (hereon pYAP1), only C3 cultures exhibited a significant increase in pYAP1 levels upon TGFβR inhibition (Fig. 9D-E). Similar levels of pYAP1 across C1-C3 cultures under basal steroid hormone-replete conditions seems contradictory in view of their significantly different CAF profiles. However, YAP1 phosphorylation at tyrosine 357 differentially redistributes YAP1 promoter occupancy in a context-dependent manner due to association of YAP1 (that is itself unable to bind DNA) with different transcription factors, such as TEAD1-4 [57–59]. Supportively, C1-C3 cultures displayed differential expression of canonical YAP-target genes (Supplemental Fig. S13C) with C3 cultures also expressing the highest levels of *TEAD3* and significantly higher levels of the YAP1-binding partner and transcription factor *VGLL4* compared to C1/C2 cultures (log2FC 0.7; *P*.adj. 0.02 in C3 vs. C2). Furthermore, ENG^+^ cells in stromogenic high-grade PCa displayed abundant nuclear pYAP1 whereas ENG^−^MCAM^−^ stromal cells in the intra-glandular tumoral space primarily associated with early/intermediate CAF (Fig. 3) were largely devoid of nuclear pYAP1 (Fig. 9F). In further support of the relevance of YAP1 signaling in the PCa TME in vivo, YAP1 target genes were abundantly expressed in the intact and castrated LAPC9 stroma and enriched in stromal cells/fibroblasts of aggressive PCa in the PCCAT, PCA and PCa GEMM single cell atlases (Supplemental Fig. S7-9, S13).

**Fig. 10:**
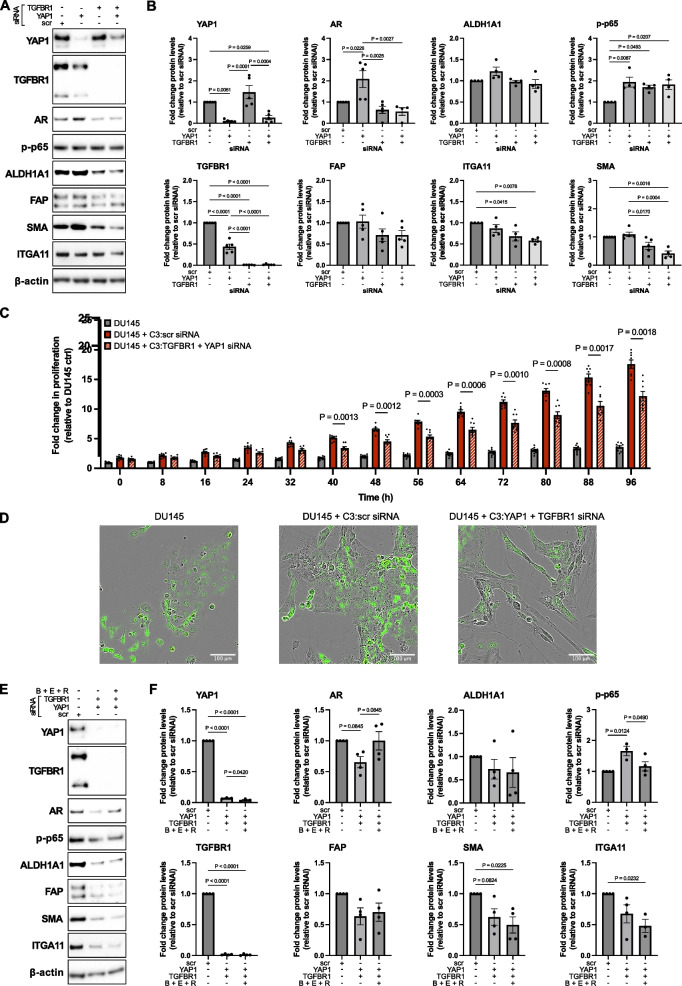
Combined knockdown of YAP1 and TGFβR1 perturbs molecular myCAF hallmarks with synergistic effects by NFκB targeting. **A** Western blotting with **B** densitometric quantification of the indicated antibodies in C3/myCAF cultures 96 h after transfection with siRNAs targeting YAP1, TGFBR1 or scrambled (scr) control. **C** Real time proliferation analysis of DU145 PCa cells stably overexpressing GFP cultured in the absence or presence of C3/myCAF pre-treated for 72 h with the indicated siRNAs. Values denote mean fold change in green fluorescent object per image from four images per well with 5 replicates per condition and two independent experiments using C3/myCAF from different patients. **D** Overlay of phase and green fluorescent images from representative wells of cells treated as in **C**. Scale bars represent 100 µm. **E** Western blotting with **F** densitometric quantification of C3/myCAF transfected for 24 h with siRNAs targeting YAP1 and TGFBR1 or scrambled (scr) control before the addition of 10 nM R1881 (R), 2 µM BAY11-7082 (B) and 10 µM ENZA (E) or vehicle equivalent for a further 72 h. **A**, **D**-**E** Images are representative of at least two independent experiments using primary cells isolated from independent donors. **B**, **C**, **F** Statistical significance was calculated via **B**, **F** one-way ANOVA with Tukey’s multiple comparison test; **C** mixed-effects model with Geisser-Greenhouse correction and Tukey’s multiple comparison test

Similar to SB431542 alone, treatment of C3 cultures with the pan-TEAD inhibitor VT107 modestly increased AR and decreased ITGA11 and SMA protein levels (lane 5 Fig. 9G, H). Mechanical changes in cell shape and substrate stiffness potently activate YAP1 [60]. Consistently, a similar downregulation of C3 markers and the YAP1 target genes *CYR61* and *CTGF* was observed when C3 but not C1/C2 cells were cultured as 3D spheroids or on soft (2 kPa) hydrogels as an alternative means to inhibit YAP1 signaling (Supplemental Fig. S14) further implicating its central role in maintaining the C3 phenotype. Since pYAP1 levels increased in C3 cultures upon TGFβR inhibition under hormone-replete conditions, we investigated whether co-targeting of YAP1-TGFβ may have synergistic effects. Indeed, C3 cultures co-treated with SB431542 and VT107 displayed strongly reduced SMA and modestly decreased FAP and ITGA11 levels, with co-treated cells also exhibiting modest increases in protein levels of AR and the C1 marker TGFBR3 (lane 8 Fig. 9G, H). The addition of R1881 alone and in combination with enzalutamide had further slight but non-significant additive effects on the protein levels of the markers examined. Like SB431542 and VT107 cotreatment, combined silencing of both YAP1 and TGFBR1 were required for robust downregulation of ITGA11 and SMA protein levels and sufficient to significantly attenuate CAF-induced proliferation of directly co-cultured DU145 and PC3 PCa cells (Fig. 10A-D and data not shown). Interestingly, YAP1 silencing alone significantly increased AR protein although it may be noted that partial downregulation of TGFBR1 was also observed upon YAP1 silencing underscoring the close inter-relationship of these signaling pathways in regulating CAF activation states.

Treatment of C3 cultures with SB431542 and/or VT107 as well as siRNA-mediated knockdown of YAP1 and/or TGFBR1 led to marked increases in serine 356 phosphorylation of the NFκB subunit p65 (lanes 2 and 5 Figs. 9G and 10A-B, hereon p-p65) with mRNA levels of the NFκB-regulated pro-inflammatory cytokines *IL6, IL8* and *IL1B* also significantly upregulated in culture on soft hydrogels or as 3D spheroids in a manner sensitive to the IKK inhibitor BAY11-7082 (Supplemental Fig. S14B-F, S16B and data not shown). In view of these observations and roles of NFκB in contributing to CAF activation [61] and suppressing *AR* transcription [62], we hypothesized that inhibition of pro-survival NFκB signaling may potentiate myCAF deactivation and promote cell death. Indeed, co-targeting YAP1, TGFβ and NFκB signaling pathways in C3 cultures via pharmacological targeting or combined YAP1/TGFBR1 knockdown in conjunction with BAY11-7082 decreased p-p65 levels, further increased TGFBR3 protein levels and potentiated ITGA11 and SMA downregulation and in the presence of enzalutamide increased cell death (lanes 12–14 Figs. 9G, H, 10E-F, Supplemental Fig. S15). The absence however of discernible upregulation of other C1 markers and failure of co-treated cells to exhibit an archetypal elongated and light refractive morphology characteristic of C1 fibroblasts (Supplemental Fig. S15 and data not shown) suggested that abrogation of key myCAF markers upon TGFβ1-YAP1-NFκB targeting does not revert C3 myCAF to a bona fide C1 state.

### Combined targeting of the YAP1-TGFβ-NFκB axis results in impaired autophagic flux and elevated sensitivity to enzalutamide-induced cell death

**Fig. 11:**
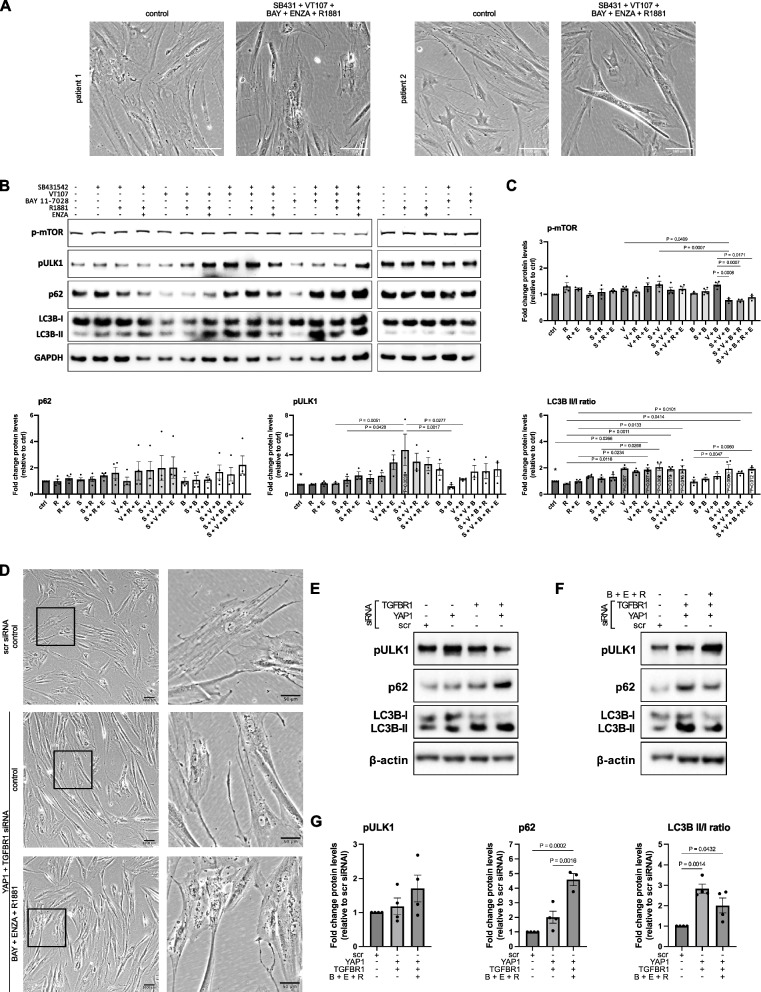
Perturbation of the YAP1-TGFβ axis results in impaired autophagic flux. **A** Representative brightfield images showing the morphology of C3/myCAF from two independent donors treated as indicated with 1 µM SB431542, 10 nM R1881, 100 nM VT107, 2 µM BAY11-7082, 10 µM ENZA or vehicle equivalent (control) for 96 h under steroid hormone-depleted conditions. **B** Western blotting with **C** densitometric quantification of the indicated antibodies in C3/myCAF cultures treated as in **A**. **D** Representative brightfield images showing the morphology of C3/myCAF 96 h after transfection with the indicated siRNA and treated for 72 h with 2 µM BAY11-7082, 10 µM ENZA and 10 nM R1881 or vehicle equivalent (control) under steroid hormone-depleted conditions. **E**–**F** Western blotting with **G** densitometric quantification of the indicated antibodies in C3/myCAF cultures treated as in **A**. **A**-**G** Data derive from at least four independent experiments using C3/myCAF isolated from different donors. **C**, **G** Values denote fold change in protein levels relative to **C** vehicle or **G** scrambled siRNA and vehicle treated control. Statistical significance was calculated via one-way ANOVA with Tukey’s multiple comparison test. For improved visualization, only selected statistically significant comparisons are shown. *P* values within bars denote statistical significance relative to control treated cells, demarcated *

C3 cultures treated with VT107 alone or with YAP1 siRNA exhibited vacuole formation, which increased upon dual inhibition of TGFβR and was further potentiated by the NFκB inhibitor BAY11-7028 suggestive of impaired autophagic flux. Enzalutamide in combination with R1881 and YAP1-TGFβ-NFκB targeting markedly further enhanced vacuole formation and was accompanied by visible cell death implying re-sensitization of cells to enzalutamide (Fig. 11A, D, Supplemental Fig. S15 and data not shown).

Consistent with its role in recycling proteins and organelles during cellular reprogramming, CAF activation is dependent on autophagy with YAP1 transcriptional activity required for the generation of autolysosomes from autophagosomes [63]. Defective autophagy leads to accumulation of the selective autophagy receptor p62, which also acts as a signaling hub to activate pro-survival NFκB signaling [64]. ULK1 phosphorylated at serine 757 (hereon pULK1) and lipidation of LC3B-I to produce LC3B-II can also increase when autophagic flux is impaired. Consistent with vacuole formation, p62 and pULK1 levels as well as the LC3B-II to LC3B-I ratio increased in cells treated with VT107 or YAP1 siRNA (Fig. 11B). These changes further increased upon combined TGFβ-YAP1 targeting indicative of impaired autophagic flux, whereby the persistent autophagic dysfunction may underlie the incomplete reprogramming to the C1/non-activated state. Notably, while p62, pULK1 and LC3B-II/I ratio remained sustained at elevated levels upon co-treatment with BAY11-7028, R1881 and enzalutamide, these cells also exhibited significant downregulation of mTOR phosphorylation at serine 2448 implying that the persistent autophagy blockage may result in nutrient stress that dampens mTORC1 activity (Fig. 11B). Collectively, these data suggest that NFκB signaling cooperates with the TGFβ1-YAP1 axis in maintaining the myCAF state, whose deactivation via TGFβ1-YAP1-NFκB targeting re-sensitizes them to enzalutamide, most likely via impaired autophagic flux.

## Discussion

Whilst widely considered important therapeutic targets, CAF subpopulations remain poorly characterized at the functional level, primarily due to the paucity of pre-clinical models. The fibroblast biobank generated here contains three distinct fibroblast subtypes: early activated iCAF associated with a favorable prognosis, an intermediate substate exhibiting molecular hallmarks of both late iCAF and early myCAF, and a subpopulation with a late-activated myCAF profile associated with poor prognostic indicators. Validation in clinical specimens, scRNA-seq datasets and PDX models underscored the physiological relevance of these distinct subpopulations across disease stages. The identification of NFκB-TGFβ1-YAP1 signaling as a key driver of stromal AR loss and the aggressive myCAF state suggest that adjuvant myCAF targeting at the level of this axis may improve castration-resistant PCa response.

The stromal response to tumor presence is not routinely evaluated in pathological tumor staging. We observed that high-grade PCa manifests as distinct stromal patterns positively (SMC-rich “non-stromogenic”) or negatively (myCAF-rich “stromogenic”) associated with patient survival. Mechanistically, intact layers of SMC are thought to restrain tumor invasion [65]. Whilst the molecular mechanisms underlying SMC loss in PCa require further investigation, the C3 marker/YAP1 target gene *CYR61 *is a secreted factor and key inducer of SMC dedifferentiation [66], implying that myCAF actively contribute to SMC loss. Distinct stromal patterns were also apparent in the two PDX models employed, whereby the myCAF profile of LAPC9 tumors contrasted with the higher expression of early-activation markers in the less aggressive BM18 model. Similarly, the myCAF profile was enriched in the aggressive PRN GEMM model that develops NEPC [29] compared to GEMM models with milder epithelial phenotypes (T-ERG, Hi-MYC, NP).

Therapy pressure, in addition to disease progression, is a central modulator of fibroblast activation dynamics [67]. Notably, human orthologs to markers defining SPP1^+ ^myCAF/CRPC-CAF (including *Spp1, Cald1, Tagln2, Tpm1, Col8a1*) that were selectively enriched following ADT treatment of the refractory prostate-specific *Pten*^*PC−/−*^*;Trp53*^*PC−/−*^ double knockout PCa model [52], were significantly upregulated in our C3 explant cultures. Moreover, lineage tracing confirmed that SPP1^+ ^myCAF arise from iCAF present in hormone-sensitive PCa [52], whereby the human orthologs of genes that delineated their iCAF (clusters 1 and 2; *Svep1, Col14a1, Pcolce2, Scara3* and *Pdgfra*) were significantly upregulated in our C1 explant cultures (Fig. 1D, Supplemental Table 3). Thus, key markers demarcating CAF subpopulations within our biobank are differentially expressed in clinical PCa and PCa GEMM by fibroblast/stromal cell subpopulations whose abundance varies during PCa progression. In particular, markers delineating C3 cultures are consistently linked to castration-resistant disease. While in vivo investigation of the potential plasticity of our explant cultures is warranted, these findings together with our observations following mechanical, pharmacological or genetic modulation of the YAP1-TGFβ1 axis as well as trajectory analyses and lineage tracing studies [10, 17, 52, 68] strongly infer in vivo fibroblast plasticity during PCa progression and therapeutic challenge. Moreover, our findings that the myCAF/C3 stromal pattern is 1) enriched in NEPC or mCRPC fibroblast clusters of human prostate single cell atlases, 2) upregulated in mouse PDX and GEMM models associated with aggressive disease and 3) associated with poor prognosis in clinical prostate cancer, further support the inclusion of stromal assessment in diagnostic staging to improve risk stratification [3].

Signaling pathways that delineated biobank culture phenotypes are consistent with the established roles of IL1-JAK-STAT, TNFα-NFκB and TGFβ1 in inducing iCAF and myCAF phenotypes [2, 69]. However, we additionally identify a critical role of YAP1 and NFκB in synergistically acting with TGFβ to maintain the myCAF state, an observation consistent with reports that unidentified stimuli are required in addition to TGFβ1 for complete activation to the myCAF state [23]. Indeed, while activation of TGFβ signaling in response to ADT was shown to be a key inducer of iCAF-to-myCAF conversion in *Pten*^PC−/−^;*Trp53*^PC−/−^mice, their comparable expression levels of TGFβ1 under sham and ADT conditions [52] indicate that also in this model additional pathway(s) underlie the specific enrichment of SPP1^+ ^myCAF/CRPC-CAF following ADT. Whilst the role of YAP1 in myofibroblast/myCAF conversion is well-established and whose silencing, similar to TGFβ2 knockdown, impaired cancer cell-induced conversion of Detox-iCAF to ECM-myCAF in vitro [10], this is to our knowledge the first time that interaction of these pathways has been demonstrated in the context of myCAF biology. The clinical relevance of our findings is underscored by the abundant expression of both YAP1 and TGFβ downstream target genes within the TME of the LAPC9 PDX model, the aggressive PRN prostate GEMM model and aggressive/resistant disease stages of clinical PCa.

Our observations that androgens promote the proliferation but suppress the migration of early activated fibroblasts imply that under hormone-naïve pre-malignant/low-grade tumor conditions, activated fibroblasts are proliferative but not highly motile. Thus, in the context of fibroblast-led cancer cell migration, such fibroblasts may restrict tumor cell dissemination, providing one potential explanation for the positive association of early activated fibroblasts with prognostic indicators of favorable outcome. Consistently, our results showed that PCa cells exhibited low motility when cocultured with C1 cultures in 3D collagen networks but migrated readily when cultured with C3/myCAF. Aggressive/hormone-insensitive PCa is frequently accompanied by stromal AR loss. We demonstrate herein that AR loss in CAF is associated with the gain of myofibroblastic hallmarks and insensitivity to AR modulation of proliferation and migration. AR expression was potently reduced by recombinant TGFβ1 and significantly upregulated in C3/myCAF upon pharmacological TGFβR inhibition or YAP1 siRNA-mediated knockdown with a similar trend observed upon pharmacological YAP1 targeting implicating the concerted suppressive action of TGFβ and YAP1 in contributing to stromal AR loss in PCa. Whilst the precise molecular mechanism remains to be determined, it is noteworthy that Smad3, downstream of TGFβR, binds to intron 3 of the *AR* gene modulating *AR* expression in PCa cells [70].

Our understanding of endogenous stromal AR function in PCa is limited and caution is erred in interpreting findings from ectopic expression models since low levels of endogenous AR in CAF are reportedly transcriptionally-incompetent and elicit an androgenic response profoundly different to the same CAF constitutively overexpressing AR [71]. Despite low AR levels and proliferative- as well as migratory-insensitivity to AR modulation, AR in C3/myCAF displays functionality as demonstrated by FAP upregulation upon enzalutamide treatment and synergistic downregulation of the myCAF marker ITGA11 in C3 cultures treated with R1881 in combination with SB431542 or VT107. Intriguingly, enzalutamide in combination with R1881 potentiated ITGA11 downregulation upon TGFβ and/or YAP1 inhibition. This apparent paradox may arise due the ability of AR to sequester and inhibit both the TGFβ signaling intermediate Smad3 and YAP1, the latter either directly or indirectly via competition for TEAD transcription factors [70, 72, 73]. We hypothesize that in the context of androgen-mediated AR stabilization, elevated AR levels engage and inhibit Smad3 thereby enhancing SB431542-mediated inhibition of TGFβ signaling. Similarly, ligand-stabilized AR may compete with YAP1 for transcription factors and thereby enhance VT107-mediated inhibition of YAP1 signaling. Notably, the AR-Smad3 interaction, which occurs via the AR ligand-binding domain, can proceed in the presence or absence of androgen [70]. Since enzalutamide inhibits ligand binding [74], it is conceivable that enzalutamide increases availability of the AR ligand-binding domain for Smad3 engagement, thereby potentiating suppressive AR-Smad3 crosstalk.

The aforementioned hypothesis alone however does not explain elevated cell death of SB431542/VT107-deactivated myCAF in the presence of R1881 and enzalutamide. This potentially clinically-relevant finding in terms of therapeutic strategies to ablate aggressive myCAF is consistent however with reports that enzalutamide activates AMPK promoting autophagy in PCa cells [75]. CAF activation is intimately linked with autophagy, a key process by which cellular components are degraded and recycled to maintain cellular homeostasis [76]. By sequestering substrates to autophagosomes, p62 acts as a selective autophagy receptor and accumulates when autophagy is defective. In this context, YAP1 transcriptional activity is required for the formation of autolyosomes from autophagosomes [63]. p62 also acts as a signaling hub and activates NFκB promoting the expression of proinflammatory cytokines such as IL6, which promotes TGFβ synthesis [77]. Collectively, findings herein lead us to hypothesize that deactivation of the myofibroblastic state upon TGFβ1-YAP1 inhibition increases demand for autophagic recycling (e.g., of cytoskeletal components) during cellular reprogramming, which however cannot fully ensue due to YAP1-impairment leading to the detrimental accumulation of autophagosomes and nutrient stress. The resulting increase in p62 activates pro-survival NFκB leading to the production of proinflammatory cytokines, including IL6, that could conceivably prime neighboring fibroblasts to undergo myCAF activation. The novel insights provided here into driver mechanisms of myCAF activation and deactivation suggest that therapeutic approaches aiming to reprogram the onco-supportive myCAF state will require combined targeting of the NFκB-TGFβ1-YAP1 axis for persistent myCAF deactivation. While further studies are required to discern the precise molecular mediators underlying enhanced cell death of deactivated myCAF in the presence of enzalutamide and persistence of the post-treatment state, our findings suggest that myCAF-targeting adjunct to ADT/ARSI may enhance therapeutic efficacy in stromogenic PCa.

In vivo preclinical studies are required to evaluate the therapeutic potential of combined targeting of the NFκB-TGFβ1-YAP1 signaling axis as an adjuvant strategy. Given the pleiotropic physiological functions of these pathways, comprehensive assessment of the specificity and toxicity of such combinatorial interventions is essential. Notably, several ongoing clinical trials targeting either TGFβ or YAP1 in various solid tumors have reported encouraging preliminary outcomes in terms of safety and therapeutic efficacy [78, 79], underscoring the translational potential of this approach.

ADT constitutes a cornerstone of current systemic treatments for advanced PCa. Ultimately however most patients relapse with lethal castration-resistant disease. We demonstrate that AR^low^/ITGA11^+^/ENG^+^ myCAF, which predominate stromogenic high-grade PCa, have a high propensity to migrate and promoted the proliferation of PCa cell migration in 3D collagen networks. This is consistent with reports that ITGA11 expression in CAF is required for CAF-induced tumor cell invasion [80] and that CAF deposit ECM tracks enriched with the YAP1 target gene and C3 marker ITGB5, which serve as guidance cues for cancer cell migration [41]. Together with the proliferative-insensitive nature of C3 to enzalutamide, these findings implicate C3/myCAF in supporting tumor cell dissemination and the development of castration-resistant PCa. Supportively, CAF expressing the C3 markers ENG or SPP1 promoted castration-resistance in PDX and PCa GEMM and expanded under castrate conditions [52, 81]. Indeed, we report that C3 myCAF are enriched in NEPC and mCRPC fibroblast clusters of human prostate single cell atlases, positively correlate with biochemical recurrence in clinical PCa, and were not only prevalent in an aggressive PCa PDX model but were resistant to castration whereupon their myCAF transcriptional profile was further enhanced.

In summary, the data presented here underscore the translational value of our fibroblast biobank for investigating functional aspects of fibroblast heterogeneity in PCa. Moreover, our findings raise important questions with regards to ADT/ARSI that may inadvertently favor survival of the aggressive myCAF substate and imply that adjuvant targeting of the myCAF state at the level of the NFκB-TGFβ-YAP1 axis may improve therapy outcome.

## Methods

### Reagents

Reagents were from Sigma-Aldrich (Vienna, Austria) unless otherwise specified. BAY 11–7082, VT107, SB431542 and enzalutamide were from MedChemExpress EU (Sollentuna, Sweden).

### Tissue processing

Four mm^3^ biopsy cores were taken by a uropathologist (G.S) from freshly excised surgical resections. A small tissue section was removed from the end of each biopsy core, formalin-fixed and paraffin embedded (together with tissue from the surrounding biopsy punch site) for subsequent histopathological evaluation via hematoxylin and eosin (HE) staining and p63/AMACR dual immunohistochemistry (Supplemental Fig. S1). The remaining biopsy core was transferred to cell culture facilities in transport solution (serum-free Dulbecco’s modified eagle media (DMEM, PAN-Biotech GmbH, Aidenbach, Germany) containing 1000 g/L glucose supplemented with 1% penicillin/streptomycin (PAN-Biotech).

### Cell culture

Prostate epithelial cell lines were obtained from American Type Culture Collection (ATCC; Rockville, MD), STR validated and maintained according to the distributor’s instructions. Mycoplasma-free human primary prostatic fibroblasts were established from prostate tissue wedges via outgrowth from ~ 1 mm^3^ tissue pieces in “collection medium” comprising DMEM containing 1 g/L glucose supplemented with 20% fetal bovine serum (FBS; PAN-Biotech), 2% Glutamax (PAN-Biotech), 1% penicillin/streptomycin and 1% ciprofloxacin. Following fibroblast outgrowth, cells were detached via trypsinization, transferred to fresh culture vessels (as passage 1) and maintained in DMEM containing 10% FBS and supplemented with 1 g/L glucose, 1% penicillin/streptomycin and 2% Glutamax (herein termed 10% DMEM). In experiments incorporating R1881, fibroblasts were pre-incubated overnight in DMEM supplemented with 1 g/L glucose, 1% penicillin/streptomycin, 2% Glutamax and 5% charcoal-treated and thus steroid hormone-depleted FBS (CTFBS) with 10 µM enzalutamide or vehicle equivalent. The following day further treatments were added as indicated in DMEM containing 2.5% CTFBS for the duration stated. Primary fibroblasts were used at passage 8 or lower. All experiments were performed at least three times with primary cells from different donors. In total, this study employed primary fibroblasts isolated from over fifty different donors. All cells were maintained in a humidified atmosphere at 37 °C with 5% CO_2_.

### 3D spheroid formation and matrix stiffness studies

For three-dimensional (3D) fibroblast spheroid generation, 20,000 cells were seeded per well of a Corning® Costar® 96-well round bottom ultra-low attachment plate (Szabo-Scandic HandelsgmbH, Vienna, Austria) in 100 µl fibroblast growth medium and centrifuged at 1200 rpm for 5 min. 18–24 replicates were prepared for each condition. For matrix stiffness studies, 6-well plates were employed containing Cytosoft® hydrogels (Advanced Biomatrix, Sigma-Aldrich) with an elastic modulus of 2 kPa, which is similar to the stiffness of healthy prostate [82], and collagen-coated prior to cell seeding.

### Conditioned media generation and application

Fibroblasts were accustomed to reduced serum conditions (2.5% FBS for 12 h) before rinsing in serum-free DMEM (GIBCO™, FisherScientific) and incubated for a further 48 h in serum-free DMEM supplemented with 0.1% BSA, 1% penicillin/streptomycin and 2% GlutaMAX™ (hereon termed SF-DMEM/0.1% BSA) in the presence of R1881, enzalutamide or vehicle equivalent as indicated. The supernatant was collected as conditioned media (CM). Non-CM control media were generated from the same mastermix of SF-DMEM/0.1% BSA but incubated for the same duration without cells. CM were centrifuged at 1000 rpm for 5 min and used immediately or frozen in aliquots for subsequent use. CM were normalized with fresh SF-DMEM/0.1% BSA according to the protein content of lysates from the corresponding cell monolayers as determined via BCA protein assay (Pierce™, Thermo Fisher Scientific, Vienna, Austria). Normalized CM were diluted 2:1 with fresh serum-free media supplemented accordingly for the corresponding tumor cell line and applied to the indicated cells for the duration stated.

### Flow cytometry and fluorescence-activated cell sorting (FACS)

Cells were detached from vessels using TrypLE (Invitrogen), stopped by dilution with PBS and after centrifugation, the pellet resuspended in 200 µl PBS containing 0.5% BSA and 2 mM EDTA (hereon flow cytometry (FC) buffer). 3 × 10^5^ cells were transferred to an Eppendorf tube and resuspended in FC buffer. 3 µl of Intratect 50 g/l infusion solution (Biotest AG, Dreieich, Germany) was added and samples incubate on ice for 5 min. Antibodies and BD Horizon™ Brilliant Stain Buffer Plus (BD Bioscience) were added as indicated (Supplemental Table 13) and samples incubated for 30 min at 4 °C protected from light. FC buffer (for FACS samples) or BD Pharm Lyse™ Lysing Buffer (for FC; BD Bioscience) was added to a final volume of 2 ml and cells pelleted by centrifugation after incubation for 10 min at RT. Cell suspensions were washed twice with 2 ml FC buffer and cells resuspended in 100 µl FC buffer and maintained at 4 °C until measurement. Approximately 5 min prior to analysis, 4 µl BD Pharmingen™ 7-AAD viability dye (BD Bioscience) was added to each sample. Flow cytometry was performed on a FACSymphony™ A5 cell analyzer (BD Biosciences) equipped with 355 nm, 407 nm, 561 nm, and 639 nm laser lines and running BD FACSDiva (v9.1) software. All antibodies were titrated in previous experiments to determine optimal staining concentration in 100 µl cell suspensions. Unstained and fluorescence-minus-one (FMO) samples were conducted for gate setting during data analysis.

For FACS, cultures were processed as above with reagent volumes adjusted for a final volume of 500 µl. After staining and washing, cells were resuspended in sorting buffer (HBSS containing 0.5% BSA, 2 mM EDTA and 25 mM HEPES). After the addition of 7AAD viability dye, samples were strained though a 100 µm mesh filter and viable cells sorted on a FACS Aria I (BD Biosciences) for cluster 1 and clusters 6 and 10 per the gating strategy outlined in Supplemental Fig. S5D whereby in one experiment (lower panel, Supplemental Fig. S5I) cells corresponding to clusters 6 and 10 were pooled into a single sample. Cells were sorted directly into Rlyse buffer for RNA isolation.

### Flow cytometry data analysis

FCS files were analyzed using FlowJo™ (BD Life Sciences, v10.10.0). Cells were gated based on size and viability stain (FSC-A/7-AAD) to exclude debris/dead cells and doublets removed (FSC-A/FSC-H) generating a single viable cell population for analysis. FC counts from all samples (n = 23) were concatenated into a single file, which retained the original sample labeling. Using the t-distributed stochastic neighbor embedding (tSNE) option in FlowJo™ dimensional reduction was performed based on seven stromal markers (MCAM, CD140a, CD140b, CD90, PDPN, FAP, CD105). The learning configuration was set to auto (opt-SNE), with 1000 iterations, a perplexity of 30, and a learning rate set to 1/12 of the count of the concatenated file. The Exact (vantage point tree) KNN algorithm and the FFT Interpolation (FIt-SNE) gradient algorithm were used. Putative subclusters were calculated with XShift based on the seven stromal markers, using 500 as the number of nearest neighbours (K), the Euclidean distance metric, a subsampling limit of 10^5, and an auto Run ID. Identified clusters were examined using ClusterExplorer and manual gating. Unstained and FMO samples were used to define gating thresholds for each stromal marker, forming the basis of the gating strategy. The developed gating strategy was then applied to additional samples (n = 13) to analyze gated clusters composition.

### RNA isolation, bulk transcriptomics, cDNA synthesis and quantitative real time PCR (qRT-PCR)

Total RNA isolation, cDNA synthesis and qRT-PCR using Taqman® gene expression assays were performed as described [43]. RNA isolation of FACS-sorted cells was performed using the RNeasy Micro Plus kit (Qiagen GmbH, Hamburg, Germany) according to the manufacturer’s instructions. TaqMan® gene expression assays (Thermo Fisher Scientific) are listed in Supplemental Table S11. One microgram total RNA was hybridized to whole genome BeadChip® Sentrix arrays (HumanHT-12v4) at the German Cancer Genome Research Centre, Heidelberg, Germany). Raw data were processed as described [83] and differential gene expression analyses performed on quantile normalized data using limma [84]. Genes displaying *P.*adj < 0.05 and log2FC > 0.3 were considered upregulated.

### Bioinformatics

Bioinformatic analysis was performed in R (v4.2.1) using both base R [85] and Bioconductor [86] packages. Fibroblast clusters were identified using principal component analysis (PCA) and hierarchical clustering using log_e_(1 + quantile normalized count) with the Euclidian distance used to calculate the distance matrix according to Ward’s linkage [87]. Comparison to publicly available fibroblast/CAF signatures was performed by calculating combined z-scores [88] using the tidy framework hacksig [89]. Gene expression or combined z-scores were visualized using EnhancedVolcano [90] and/or pheatmap [91]. Pathway analysis was performed using clusterProfiler [92], msigdbr [93] and Pathway RespOnsive GENes (PROGENy) for activity inference [94]. YAP1 target genes were defined as reported [95]. R clustering of FC data was performed in R (v4.2.1) using RStudio (v2022.7.1.554) incorporating base R functionalities [85] and additional packages. Data manipulation was carried out with the tidyverse package (v2.0.0) [96]. Heatmaps were generated using the pheatmap package (v1.0.12) [91], with color gradients applied via the RColorBrewer package (v1.1–3) [97]. The percentages of the gated clusters in each sample were visualized as a heatmap and scaled per sample to highlight the most prominent clusters in each sample, facilitating the grouping of samples into broader categories. The percentages of the original clusters positive for each of the seven stromal markers or the C1, C2 or C3 subpopulations were visualized as a heatmap without scaling. Single cell-based analyses of the pan-cancer CAF Atlas [12] (https://gist-fgl.github.io/sc-caf-atlas/), Prostate Cancer Cell Atlas [27] (https://pccat.net/) and Prostate Cancer Atlas [28] (https://www.prostatecanceratlas.org/) were conducted using the publicly-available web interfaces using the filter options “stromal cells” (PCCAT) or “microenvironment “ and “fibroblasts” (PCA). Analysis of prostate GEMM mesenchymal cells was performed using the previously published scRNA-seq data from Pakula et al. [29]. The normalized count matrix and cell annotations for the mesenchymal PCa dataset were downloaded from 10.5281/zenodo.7452769 and analyzed using Seurat [98]. UCell scores [99] were calculated to evaluate the expression of gene signatures associated with selected pathways or cell types. Lineage analysis and pseudotime inference was performed using Slingshot [100]. The analysis was performed on fibroblast clusters only (omitting the smooth muscle cell clusters) and cluster c1 was selected as the starting cluster.

### Analysis of TCGA and SU2C data

RNA sequencing and clinical data of TCGA prostate adenocarcinoma (PRAD) [101] samples were downloaded from UCSC Xena (https://xenabrowser.net/datapages/). Combined z-scores were calculated for bulk transcriptomic-derived gene signatures as described above and compared between clinical parameters. The R package ggsignif [102] was used to perform t-tests. For visualization as heatmaps, only samples with the sample type “primary tumor” were selected. Disease-free survival (DFS) analyses were performed using GEPIA2 [103] using the group cut-offs indicated in the corresponding figure legend. Bulk transcriptomic-derived signatures were generated from the top 5 marker genes for each fibroblast cluster ranked according to *P.*adj value with log2 fold change (log2FC) > 0.5. Gene signatures are provided in the Source Data file. The SU2C metastatic prostate adenocarcinoma dataset [104] was analyzed using cBioportal [105]. Samples were divided based on expression of the top 15 CAF-subtype specific markers using OQL. A sample was considered “high” for one CAF subtype if 3 or more CAF subtype-specific markers were > z-score of 1 (number of samples/patients: C1 = 93/93, C2 = 103/101, C3 = 93/91). Samples [92] that overlap in the selected groups were excluded from sample-level analysis which resulted in 34, 29 and 24 samples for C1, C2 and C3, respectively. Clinical attributes were compared between those groups. For analysis of SMC gene expression on survival in high-grade PCa, the TCGA-PRAD cohort (n = 500) was initially stratified into high- (≥ T2c) and low-grade (≤ T2b) tumors based on pathological T stage. The data were subsequently filtered according to the following criteria: 2 patients were excluded due to prior treatment, and 29 patients with low-grade tumors were excluded from the survival analysis. The top 20 upregulated genes from prostate SMC gene signatures were curated from relevant literature [18, 106] and are provided in the Source Data file. These gene lists were condensed into gene signature expression values using the GSVA R package (v1.50.5). The filtered TCGA PRAD cohort (n = 469) was then stratified into "high" and "low" SMC expression groups, with the threshold determined by the optimal cutpoint calculated using the surv_cutpoint() function from the survminer R package (v0.4.9). Survival analysis was conducted using the survival R package (v3.7–0), and Kaplan–Meier curves were generated with the ggsurvplot() function from survminer (v0.4.9).

### Cell size analysis

Subconfluent fibroblast cultures were trypsinized and cell size determined on a CASY cell counter and analyzer (Schärfe-System GmbH, Reutlingen, Germany) equipped with a 150 µm capillary using the following settings: evaluation cursors 11.18–50.00 µm, normalization cursors 6.88–50.00 µm, smoothing 0 and automatic aggregation correction.

### SDS-PAGE and western blotting

Preparation of cell lysates, normalization via BCA protein assay (Pierce, Thermo Scientific), SDS-PAGE and Western blotting were performed as described [43] using antibodies as indicated in Supplemental Table S12. Densitometric quantification of background-subtracted images relative to GAPDH or β-actin loading controls was performed using the analysis tools in the Image Lab 6.1 (Bio-Rad Laboratories) or Image Studio™ (LICORbio GmbH, Bad Homburg, Germany) software for chemiluminescent- or fluorescent-detected images, respectively.

### Proliferation assays

Cells seeded in quadruplicate in black 96-well plates in phenol red-free media were treated for 96 h as indicated. WST-1 assays (Sigma Aldrich) were performed according to the manufacturer’s instructions before absorbance measurement at 490 nm on a BioTek Cytation 5 plate reader (Agilent Technologies Österreich GmbH, Vienna, Austria) running BioTek Gen5 software (version 3.09, Agilent Technologies Österreich GmbH). Background intensity from cell-free wells was subtracted. Thereafter media were aspirated and cells lyzed by freezing at −80 °C. Upon thawing, 200 μl CyQuant lysis reagent (Invitrogen, Vienna, Austria) was added containing SYBR® Green I (diluted 1:1000). Following incubation for 30 min at 37 °C, fluorescence was measured on a BioTek Cytation 5 plate reader (Agilent Technologies Österreich GmbH) and background intensity from cell-free wells subtracted. A replica seeded plate harvested immediately upon cell attachment (< 4 h) was used to normalize potential differences in seeding densities between different fibroblast cultures. Real time fibroblast proliferation assays were conducted as above but analyzed on an Incucyte ® S3 Live-cell analysis instrument (Sartorius Lab Instruments GmbH and Co. KG, Gladbach, Germany). Images were acquired every 12 h and the phase object confluence determined. For real time co-culture proliferation assays, 10,000 fibroblasts were seeded in quadruplicate in DMEM containing 2.5% CTFBS supplemented with 1 pM R1881 with 10 µM enzalutamide or vehicle equivalent. After 48 h, 1500 DU145 or PC3 stably transfected to overexpress GFP were overlaid per well and real time proliferation (green fluorescence) measured every 6 h on a BioTek Cytation 5 plate reader (Agilent Technologies Österreich GmbH) at 37 °C with 5% CO_2_. Background intensity from cell-free wells was subtracted. Phase and GFP images were acquired on a JuLI Smart fluorescent Cell viewer (NanoEntek Europe, Martinsried, Germany).

### Migration assays

Migration assays using 24-well Fluoroblok transwell plates with an 8 μm pore size (BD Bioscience, Vienna, Austria) were performed as described [43] with the exception that fluorescence was measured on a BioTek Cytation 5 plate reader (Agilent Technologies Österreich GmbH) in bottom read mode. Phase and GFP images were acquired on a JuLI Smart fluorescent Cell viewer (NanoEntek Europe, Martinsried, Germany).

### 3D co-culture

For 3D cell co-cultures, glass-bottom dishes were precoated with 0.1% poly-L-lysine (PLL) for at least 30 min at 37 °C prior to collagen addition. A collagen mixture (type I collagen, BD Biosciences; 5:1 ratio of unlabeled to Alexa Fluor 555–labeled collagen) was prepared on ice at a final concentration of 2.2 mg/mL and combined with GFP^+^ cancer cells and primary fibroblasts at a 2:1 ratio. From this mixture, 30 µL droplets were pipetted onto the PLL-coated glass-bottom dishes, allowed to polymerize at room temperature for 30 min, and subsequently overlaid with pre-warmed DMEM supplemented with FCS. Samples were then maintained in a cell incubator. After 24 h, live imaging was performed using a spinning disk microscope equipped with a 10 × air objective (NA 0.30). Image stacks spanning 140 µm were acquired every 10 min for a total of 8 h. Maximum-intensity projections were generated and videos stabilized using the FIJI plugin Registration – Linear Stack Alignment. GFP^+^ cancer cells were tracked using the FIJI plugin TrackMate to calculate mean migration speed.

### Immunofluorescence of 3D co-cultures

Fluorescent collagen droplets containing co-cultures of GFP^+^ cancer cells and primary fibroblasts (2:1 ratio) were fixed with 4% paraformaldehyde for 10 min. Phalloidin–Alexa Fluor 647 (Invitrogen) was added for 45 min, followed by washing with PBS. Imaging was performed on a Leica SP8 confocal microscope equipped with HyD detectors and a white light laser, using a resonant scanner (8000 Hz) and a 20 × water-immersion objective (NA 0.75). Acquired image stacks were analyzed in FIJI. Cells were manually segmented and the circularity of GFP^+^ cancer cells quantified.

### siRNA-mediated knockdown

One hundred thousand fibroblasts were transfected with 20 nM of siRNA (Supplemental Table 13) using Lipofectamine RNAiMAX transfection reagent diluted in Opti-MEM (both Thermo Fisher Scientific) per the manufacturer’s instructions and seeded to one well of a 6-well plate. For co-culture proliferation assays, cells were trypsinized 72 h post transfection and seeded at a density of 10,000 fibroblasts per well of a black 96-well plate in 10% DMEM in quintuplicate. Following fibroblast attachment (∼8 h), 1500 DU145 or PC3 cells stably overexpressing GFP were overlaid per well and incubated in an Incucyte ® S3 Live-cell analysis instrument (Sartorius Lab Instruments GmbH and Co. KG, Gladbach, Germany). Phase and green fluorescent images were acquired every 12 h and PCa cell proliferation measured as the green object count per image.

### Patient-derived xenografts (PDX)

The subcutaneously maintained femoral bone metastatic PCa BM18 and LAPC9 PDX models have been previously described [48, 49]. Briefly, serially passaged tumors were subcutaneously implanted into 6-week-old CB17 SCID male mice. Once tumors established, hosts were left intact or underwent castration via bilateral orchiectomy and tumors harvested 8 (LAPC9) or 14 (BM18) days later. Since host stromal cells infiltrate human PCa PDX tissue and eventually replace human stromal tissue after serial passaging [50, 51], the stromal and tumoral transcriptomes of harvested tumors could be distinguished in bulk RNA-sequencing data as mouse- or human-specific reads, respectively. BM18 and LAPC9 PDX FFPE tissue blocks and bulk RNA-sequencing data (European Genome-Phenome Archive, Accession number EGAS00001004770) used herein derived from our previous study [50].

### Immunocytochemistry and immunohistochemistry

For immunohistochemistry, 2 µm FFPE tissue sections from consenting treatment-naive patients were stained on a Ventana Benchmark Ultra automated staining device (Roche Diagnostics GmbH, Vienna, Austria) with the antibodies denoted in Supplemental Table S13. For multiplex immunofluorescent staining, tissue sections were deparaffinized and rehydrated in a graded alcohol series. Antigen retrieval was performed via indirect boiling in Dako Target Retrieval Solution pH 9 (Agilent) for 10 min. After cooling, sections were blocked in 3% BSA in Tris-buffered saline (TBS) before sequential incubation for 1 h at RT or overnight at 4 °C with primary antibodies diluted in 0.5% BSA in TBS as indicated (Supplemental Table 13). Washed sections were incubated with fluorescently conjugated secondary antibodies (Supplemental Table 13) for 1 h at RT. The cycle was repeated until all antibodies had been stained. Nuclei were counterstained with 2.5 µg/ml Hoechst 33342 (Invitrogen, Thermofisher) before mounting in VECTASHIELD® mounting medium for fluorescence (Vector Laboratories, Inc., Burlington, CA). Images were acquired on a Zeiss Axio Imager Z2 microscope (Zeiss, Vienna, Austria) equipped with a Pixelink PL-B622-CU camera for brightfield imaging and a monochrome pco.edge 4.2LT camera for fluorescence imaging. TissueFAXS® software (version 7.137, TissueGnostics® GmbH, Vienna, Austria) was used to acquire images via a 20 × air objective. Image acquisition settings for all cell type markers remained constant. For visualization purposes, exposure time for the nuclear counterstain differed due to the non-uniform staining intensity of some tumor cells, intense staining of some PIN foci and lower nuclear content of euploidy cells. Single channel monochrome images were merged and pseudo-colored for visualization as indicated in the corresponding figure legend. Quantification of immunofluorescent images were performed in ImageJ 1.54j (Rasband, NIH, USA). Area quantification was performed on fields of view of constant size and with constant threshold settings using ImageJ. To account for different tissue morphology and areas without cells (e.g. in prostate glands), the quantified area was normalized to the total DAPI^+^ area and is displayed in percent. Masks were created to calculate the DAPI^+^ area (all nuclei), DAPI and AR area (AR^+^ nuclei) and applied to masks created in the stromal marker-specific channel to select for the DAPI^+^AR^+^ area for each stromal marker. Data are shown as a percentage of all AR^+^ nuclei positive for each stromal cell marker relative to the total area positive for DAPI and the corresponding stromal cell marker.

### Statistics and reproducibility

Unless otherwise specified cell line data are shown as mean ± SEM. *n* represents the number of biological replicates as stated in the corresponding figure legends. All experiments were repeated independently at least three times using primary fibroblasts isolated from different donors. Statistical analyses were performed using the R statistical environment (v4.2.1) [85] or GraphPad Prism (v9.5.0 GraphPad Software, LLC). Methods used for each statistical analysis are specified in the corresponding figure legend or relevant section of the Materials and Methods. Unless otherwise specified, statistical significance is denoted: NS, not significant; *, *P* < 0.05; **, *P* < 0.01; ***, *P* < 0.001.

## Supplementary Information

Supplementary Materia 1: Supplemental Figure 1. Isolation and characterization of ex vivo culture of primary prostate fibroblasts. A) radical prostatectomy tissue wedges (i) containing regions of suspected malignancy or benign-adjacent tissue were sampled (ii) using 4 mm^3^ biopsy punchers. The top part of each biopsy core was removed (iii) for FFPE processing (iv) and subsequent histopathological evaluation. Remaining tissue was quartered (v) and similarly processed. B) Histopathological validation of the top section of biopsy cores from (Aiii-iv) via HE staining and p63 (brown) and AMACR (red) dual-immunohistochemistry. C) The remaining biopsy core was cut into small pieces and transferred to culture medium that supports fibroblast but not endothelial cell growth. Outgrowing fibroblasts were selectively enriched via trypsinization from any epithelial cells, which require additional collagenase treatment for detachment. D) Representative images of three primary fibroblast explant cultures isolated from different patients stained for mesenchymal markers vimentin (green) and CD90 (white) and the epithelial marker pan-cytokeratin (red). Nuclei were counterstained using Hoechst (blue). 22Rv1 PCa cells served as positive control for pan-cytokeratin (far right, upper panel). Negative control of a parallel stained fibroblast culture incubated without primary antibodies (far right, lower panel). Supplemental Figure 2. Ex vivo culture of primary prostate fibroblasts – pertaining to Supplemental Fig. 1. Single channel monochromatic images (from Supplemental Fig. 1D) of immunofluorescent validation of three primary fibroblast explant cultures isolated from different patients stained for mesenchymal markers vimentin (green) and CD90 (white) and the epithelial marker pan-cytokeratin (pan CK, red). Nuclei were counterstained using Hoechst (blue). 22Rv1 PCa cells served as a positive control (pos. ctrl) for pan-cytokeratin. Negative control (neg. ctrl) of a parallel stained fibroblast culture incubated without primary antibodies (far right, lower panel). Supplemental Figure 3. Transcriptomic analysis of primary prostate fibroblast cultures identifies two distinct CAF populations - pertaining to Figure 1 and 2. A) Heatmap depicting sample level expression of significantly upregulated genes (*P*.adj<0.05, log2FC>0.3) for each fibroblast cluster in the transcriptomic dataset (related to Fig. 1 C). B) clusterProfiler analysis of left: GO Biological Process for significantly upregulated genes of each fibroblast cluster, including two cycling CAF explant cultures (cycling), centre and right: the top 20 scoring Hallmark and REACTOME pathways for significantly upregulated genes of each fibroblast cluster (related to Fig. 1E-G). C-D) Expression of the indicated genes across each fibroblast cluster. Values denote C) average log2 signal intensity from quartile normalized bulk transcriptomic data and D) mean 2^-ΔΔCt^ via qRT-PCR relative to the housekeeping gene TBP. Error bars denote S.E.M. Significance was calculated via a Kruskal-Wallis test with multi-comparison correction using the two-stage linear step-up method of Benjamini, Krieger and Yekutieli. E) Comparison of expression profiles for each fibroblast cluster with published scRNA-seq datasets using combined z-scores for each gene signature and represented as mean per fibroblast cluster (related to Fig. 1I-J). F) Expression of signature genes (combined z-score) for each primary fibroblast cluster in the TCGA prostate adenocarcinoma (PRAD) cohort (related to Fig. 2A-D). Boxplots were used to compare signature gene expression levels across Gleason scores, T and N stages and biochemical recurrence. The R package ggsignif was used to perform t-tests. G) Expression levels of substate-delineating markers used to perform survival analyses (Fig. 2 C) in different cell types of the Heidegger PCa scRNA-seq dataset. Source data for panels A-C, E-F are provided in the Source Data file. Supplemental Figure 4. Activation trajectory of fibroblast subpopulations- pertaining to Figure 1. A-C) Inferred pseudotime trajectory of CAF activation in cells annotated as CAF in the pan-cancer single cell CAF Atlas (1) available at https://gist-fgl.github.io/sc-caf-atlas/. For orientation, A) the distribution of the original fibroblast clusters, pseudotime and cell activation state and B) expression of canonical markers for distinct stromal cell types are depicted. C) Distribution of C1-C3 fibroblast subpopulation markers on the inferred CAF activation trajectory. Supplemental Figure 5. Primary explant cultures depict distinct stable homogeneous fibroblast phenotypes. A) Sample level heatmap (clustered according to ward.D) for qRT-PCR-derived 2^-ΔΔCT^ values of the indicated genes in an independent cohort of 29 primary human prostate explant cultures (qRT-PCR cohort) and fresh cultures from 3 donors used in the original bulk transcriptomic cohort. Sample numbers are indicated beneath the heatmap. Font color denotes the subpopulation annotated using the different methodologies (C1, blue; C2, yellow; C3, red; n.d., not determined). B) Expression level of the indicated genes in patient-matched C1 and C2 cultures and a non-matched C3 culture maintained longitudinally for the indicated number of passages. Data are representative of longitudinal cultures from a total of six different donors. C) Immunofluorescence using the antibodies stated against the indicated markers in three different primary human prostate explant cultures that clustered in the independent explant cohort (A) into C1/C2/C3 subpopulations. Font color denotes pseudo-coloring in the displayed merged images. Nuclei were counterstained using Hoechst 33342. Images are representative of three experiments using explant cultures from seven different patients. Supplemental Figure 6. Flow cytometry-based single cell analysis of primary human prostate fibroblast cultures. A-H) Flow cytometry analysis of thirty-six biobank cultures selected randomly or on the basis of their substate annotation per the original transcriptomic dataset (Supplemental Table S2) and/or qRT-PCR of substate-delineating markers (Supplemental Fig. S4 A) and stained for the cell surface stromal markers FAP, PDPN, CD90/THY1, CD140b/PDGFRB, CD140a/PDGFRA, MCAM and CD105/ENG. A-B) tSNE dimensional reduction analysis of flow cytometry (FC) data from twenty-three primary prostate fibroblast cultures (training set) based on their expression of the aforementioned stromal makers. Sample counts are stratified to visualize A) the original 13 clusters identified via XShift or B) sample number. C) Heatmap of flow cytometry data displaying percentage of counts in the positive marker gate and percentage of counts in samples annotated as C1, C2 or C3 subpopulations as defined and marked * in panel H). D) Gating strategy developed using the most distinguishable stromal surface markers identified from C), which yielded eight gated clusters from the original 13 clusters. E) tSNE plot overlaid with the 8 gated clusters identified by applying the gating strategy in D). F) Heatmap clustered based on the percentage of gated clusters for 36 fibroblast cultures (extended set), including the 23 explant culture samples from the training set. FC sample numbering of the extended set corresponds to that of the training set in the tSNE plot in A). Annotation of donor-related cultures that were also used in the qRT-PCR cohort (Supplemental Fig. S4A) and original transcriptomic cohort (Fig. 1, Supplemental Table 2) are depicted underneath the corresponding FC sample number. Data are scaled by column to highlight the most prominent cluster in each sample. G) tSNE plot overlaid with sample counts from cultures annotated as C1, C2 or C3 subpopulations as defined and marked * in panel H). Grey shading denotes ungated cells of samples that did not display a single enriched cluster or were not determined (n.d.). H) Stacked bar plot displaying the percentages of gated clusters within each primary prostate fibroblast culture sample (n = 36), grouped according to the predominant gated cluster composition of each sample. Sample numbering beneath each bar corresponds to the sample numbering in F). Cultures enriched for a single cluster (≥55%) were annotated as C1, C2 or C3 (marked *). Cultures enriched for two of either clusters 6 and 10 or clusters 10 and 1 were annotated as C1/C2 or C2/C3, respectively. All other cultures were designated n.d. (not determined). I) qRT-PCR of the indicated gene in primary prostate fibroblast cultures sorted via FACS for cluster 1, 6+10 combined (*lower panel*) or cluster 6 or 10 separately (*upper panel*). Genes associated with general fibroblast activation/contractile markers are indicated in black, while C1, C2, and C3 subpopulation-delineating markers (from Fig. 1) are shown in blue, yellow, and red, respectively. Values represent mean 2^-ΔΔCt^ via qRT-PCR, normalized to the housekeeping gene TBP. Data are representative of two independent sorting experiments using five fibroblast cultures isolated from different donors. F-H) Font color denotes the subpopulation annotated using the different complementary methodologies (C1, blue; C2, yellow; C3, red; black font, not determined; black font with yellow-red box denotes cultures considered to comprise a mix of C2/C3 substates). Supplemental Figure 7. Expression of fibroblast subpopulation-delineating markers across PCa disease stages - pertaining to Figure 2E-G. UMAP visualization of stromal cell A) cluster distribution and B) feature plots displaying expression of the indicated genes across different disease stages in the single cell Prostate Cancer Cell Atlas [27] available at https://pccat.net. For clarity, only fibroblast clusters are displayed. Supplemental Figure 8. Expression of fibroblast substate-delineating marker genes during PCa progression - pertaining to Figure 2G. Bar plots depicting expression scores of A) the indicated fibroblast substate markers and B) genes associated with signaling by TGFβ, YAP or AR in cells annotated to the fibroblast lineage in the single cell Prostate Cancer Atlas [28] across different disease stages (mCRPC, metastatic castration-resistant PCa) available at https://www.prostatecanceratlas.org/home. Supplemental Figure 9. Expression of murine orthologs to fibroblast substate markers across PCa GEMM models of differing aggressiveness – pertaining to Figure 2H. A) Mutational profile and phenotypes of the Pakula et al. scRNA-seq study of prostate GEMM models [29]. B- J) Analysis of murine orthologs to fibroblast substate markers across the mesenchymal cell clusters of the Pakula et al. study. B-C) Visualization of mesenchymal annotated cells in the scRNA-Seq analysis and distribution across B) GEMM model or C) mensenchymal cell clusters as determined by Pakula et al. D) Feature plots of Ucell scores for the top five upregulated C1, C2 or C3 orthologous marker genes (*P.*adj<0.05, log2FC>0.3) on the Pakula mesenchymal cell clusters. E-F) Unscaled dotplots of UCell expression scores for the top five upregulated C1-C3 orthologous marker genes per E) mesenchymal cell cluster or F) PCa GEMM model. G-H) Dotplots of Ucell expression scores for murine orthologs to the HALLMARK TGFbeta signaling gene set or Cordenonsi_YAP_Conserved_Signature msigdb gene set (M2871) per G) mesenchymal cell cluster by Pakula et al. or H) PCa GEMM model. Expression scores are scaled by column. I-J) Slingshot pseudotime trajectory of I) Pakula mesenchymal cell clusters or J) expression levels of murine orthologs to C1 (*Hmox1*), C2 (*Aldh1a1, Apod, A2m*) and C3 (*Col8a1*) fibroblast substate markers across pseudotime_5 spanning cluster c1-c5-c7. I) Cluster c1 was defined as start and the SMC/pericyte cluster (pc) c0 was omitted. Supplemental Figure 10. Multiplex immunofluorescent and immunohistochemical staining of human PCa reveals distinct CAF subpopulations in vivo – pertaining to Figure 3. A-C) Human prostate tissue sections of indicated pathology were stained using the antibodies shown. Original magnification 200x. Font color denotes pseudo-coloring in the displayed images. A-B) Nuclei were counterstained using Hoechst 33342 (blue). Boxed regions are shown enlarged beneath each parental image. B) Four-channel merged image of a negative control incubated without primary antibodies. C) Immunohistochemistry of consecutively stained sections of indicated pathology using the antibodies indicated. D) Immunohistochemistry of CES1 in low-grade PCa. Boxed region is enlarged right. A-C) Images are representative of 4 independent experiments using tissue sections derived from 8 different patients. E-G) Expression of SMC signature genes (combined z-score) in the TCGA-PRAD cohort using the indicated signatures. H-I) Kaplan-Meier plots depicting progression-free survival (PFS) of high-grade (^≥^T stage T2c%) samples of the TCGA-PRAD cohort stratified as described in Materials and Methods for high (SMC^high^) or low (SMC^low^) expression levels of the indicated SMC signatures. Source data for panels C-I are provided in the Source Data file (Supplemental Table 6). Supplemental Figure 11. AR loss in myofibroblastic CAF renders them insensitive to the proliferative effects of enzalutamide - pertaining to Figure 6. A) Western blotting of thirteen partially patient-matched explant cultures using the antibodies indicated (original western blot sourcing the densitometric data shown in Fig. 6D). B) Densitometric-based correlation of AR and pSTAT3 or ITGA11 protein levels in thirteen explant cultures as depicted in A). C-D) Immunofluorescent staining of human prostate cancer tissue sections using the antibodies indicated. Font color denotes pseudo-coloring in the displayed merged images. Nuclei were counterstained using Hoechst 33342. Boxed regions are shown enlarged, right. Arrowheads in panel 2 indicate non-SMC (CCDC102B^-^) AR^+^ stromal cells. E-F) ImageJ quantification of tissues stained as in D) whereby the area stained by the indicated antibody is expressed as a percentage of the total DAPI^+^ area, panel E). For AR colocalization in panel F) the percentage area is shown for AR^+^ nuclei (DAPI^+^ and AR^+^) also positive for each stromal cell type marker (e.g. DAPI^+^ and AR^+^ and ENG^+^). The quantification does not distinguish between ENG^+^ myCAF and ENG^+^ endothelial cells, the latter comprising a key source of ENG^+^AR^+^ cells in high grade stromogenic tumors. Data represent mean ± SEM of ten fields of view from three different high-grade tumors stratified according to their stromogenic or non-stromogenic content defined as myCAF-rich (ENG^+^/ITGA11^+^) or SMC-rich (SMA^+^/CNN1^+^). G-I) Real time proliferation of the indicated fibroblast cultures incubated G) in 10% steroid hormone-replete DMEM with 10 µM enzalutamide or vehicle equivalent (ctrl) or H-I) in 2.5% steroid hormone-deplete DMEM with the indicated concentration of R1881 and 10 µM enzalutamide or vehicle equivalent (related to Fig. 7D-E). Statistical significance was calculated: B) Pearson correlation, E-F) two-way ANOVA with Tukey’s multiple comparison correction, G-I) two-way ANOVA with Holm-Šídák multiple comparison correction. A, C, D) images are representative of at least three independent experiments using primary material from different donors. Supplemental Figure 12. Dynamic remodeling of CAF activation states in vivo - pertaining to Figure 8. A) Sample level heatmaps depicting expression level of the corresponding murine orthologs for the indicated explant culture signature genes (related to Fig. 8B) in the mouse transcriptome of castrated and intact samples from the indicated PDX model. B-C) Single channel monochromatic images of images displayed in Fig. 8D and Supplemental Fig. 12D of the indicated PDX tumor using the antibody indicated. D) Additional fields of view of the indicated PDX tumor stained using the antibodies indicated. E) Immunohistochemistry of mouse Itga11 or IgG control in LAPC9 intact tumors. Enlarged images of boxed regions are shown beneath each parental image. B-E) Images are representative of at least two independent experiments using tissue sections from at least two different tumors per condition. F-G) Expression of marker genes from F) the Pakula mesenchymal cell clusters of different PCa GEMM models [29] or G) the Wang fibroblast clusters from *Pten*^*PC−/−*^ and *Pten*^*PC−/−*^; *Trp53*^*PC−/−*^ double knockout mice with and without ADT [52] across castration-sensitive BM18 and castration-resistant LAPC9 prostate PDX models. Supplemental Figure 13. TGFβ and YAP signaling defines C3/myCAF fibroblast subtypes across different models in vitro and in vivo. A-K) Gene expression across the different models and fibroblast/mesenchymal cell clusters of single cell PCa datasets for the pathways HALLMARK_TGFbeta_signaling, MSigDB gene set Cordenonsi_YAP_conserved_signature (M2871) [93], murine canonical pathway CP: Wikipathway TGF-beta receptor signaling pathway and TGFβ regulated genes in CAF as determined by Yeung et al. [107]. Gene expression of respective pathways in A-C) fibroblast explant cultures or D-F) castration-sensitive BM18 and castration-resistant LAPC9 PDX models. G) Expression of pathways in fibroblasts across disease stages in the single cell Prostate Cancer Atlas [28] (related to Supplemental Fig. S8). H) Expression of YAP target gene *PDLIM2* in mesenchymal cell clusters of the Prostate Cancer Cell Atlas (right panel) whereby the red box demarcates fibroblasts enriched in mCRPC and NEPC (left panel, related to Supplemental Fig. S7). (I-K) Expression of pathways in the mesenchymal cell clusters of prostate GEMM models (J) per cluster or K) per model as determined by Pakula et al. [29] (related to Supplemental Fig. S9). For clarification, I) shows cluster distribution per GEMM model as determined by the UCell score of the top 20 marker genes from each cluster. Supplemental Figure 14. Signaling by an NFκB-YAP-TGFβ axis underlies AR loss during fibroblast phenotypic switching - pertaining to Figure 9. A) qRT-PCR of C1 and C3 cultures treated with 1 µM SB431542 or vehicle control for 72h. Values represent mean fold change ± SEM in expression relative to vehicle control treated cells. B) Morphology and C) mean fold change gene expression (qRT-PCR) of the indicated fibroblast cultures grown as 3D spheroids relative to standard 2D conditions for 4 days. D) Morphology and E) mean fold change gene expression (qRT-PCR) of two C3 cultures on 2 kPa soft hydrogels relative to standard tissue culture plasticware (TCP) for 4 days. F) qRT-PCR of *IL6 *and *IL8 *in C3 cultures grown as 3D spheroids in the presence of 2 µM BAY11-7028 (BAY) or vehicle equivalent (ctrl, control) relative to standard 2D conditions in the presence of vehicle for 4 days. Data derive from eight independent experiments using primary cells from different donors. Statistical significance was calculated: A, D) two-way ANOVA with Šídák multiple comparison correction, F) Kruskal-Wallis test with two-stage linear step-up of Benjamini, Krieger and Yekutieli multiple comparison test. B, D) Images are representative of at least three independent experiments using primary material from different donors. Supplemental Figure 15. Signaling by an NFκB-YAP-TGFβ axis underlies AR loss during fibroblast phenotypic switching - pertaining to Figure 9G and 11A. A-C) Brightfield imaging of three C3 cultures isolated from independent patients and treated with A, C) the indicated compound for 96 h (10 nM R1881, 1 µM SB431542, 10 µM enzalutamide (ENZA), 100 nM VT107, 2 µM BAY11-7082 or vehicle equivalents) or B) the indicated siRNA for 96h. C) For visualization, inhibitors are abbreviated B, BAY11-7082; E, enzalutamide; R, R1881; S, SB431542; V, VT107. Scale bars denote A-B) 100 µm or C) 500 µm. Supplemental Figure 16. Combined knockdown of YAP and TGFBR1 perturbs molecular myCAF hallmarks with synergistic effects by NFκB targeting - pertaining to Figure 10. A-C) qRT-PCR of the indicated genes in C3/myCAF explant cultures 96 h after co-transfection with YAP1 and TGFBR1 siRNAs. Values represent mean fold change in expression relative to scrambled siRNA control from four independent experiments using primary cells from different donors. Statistical significance was calculated via one sample t test.

Supplementary Material 2. PCa cell invasion in 3D collagen matrices upon co-culture with C1 primary fibroblasts – related to Fig. 5. DU145 PCa cells stably overexpressing GFP (cyan) were embedded with C1 fibroblasts in a composite 3D network of fluorescent collagen fibers (red) and imaged for 8 hours at a rate of one image every 10 minutes using a spinning disk microscope. Time is indicated in minutes. Scale bar represents 50 µm

Supplementary Material 3. PCa cell invasion in 3D collagen matrices upon co-culture with C3 primary myCAF – related to Fig. 5. DU145 PCa cells stably overexpressing GFP (cyan) were embedded with (right panel) or without (left panel) C3 fibroblasts in a composite 3D network of fluorescent collagen fibers (red) and imaged for 8 hours at a rate of one image every 10 minutes using a spinning disk microscope. Time is indicated in minutes. Scale bar represents 50 µm

Supplementary Material 4.

## Data Availability

Human bulk transcriptomic explant culture dataset is available in ArrayExpress under accession number E-MTAB-13167. Mouse RNA-sequencing reads for the PDX models are available in the European Genome–Phenome Archive database under accession number EGAS00001004770. Source data are provided in the supplementary file. No new code was developed for this manuscript.

## Abbreviations

2D: Two-dimensional
3D: Three-dimensional
ADT: Androgen deprivation therapy
apCAF: Antigen-presenting cancer-associated fibroblast
AR: Androgen receptor
ARSI: Androgen receptor signaling inhibitor
BSA: Bovine serum albumin
CAF: Cancer-associated fibroblasts
CES1: Carboxylesterase I
CM: Conditioned media
CRPC: Castration-resistant prostate cancer
CTFBS: Charcoal treated fetal bovine serum
DFS: Disease-free survival
DMEM: Dulbecco’s modified eagle media
ECM: Extracellular matrix
EMT: Epithelial-to-mesenchymal transition
ENG: Endoglin
FAP: Fibroblast activation protein
FBS: Fetal bovine serum
FFPE: Formalin-fixed paraffin-embedded
GO: Gene ontology
h: Hours
HBSS: Hank’s balanced salt solution
HE: Hematoxylin and eosin
HGPIN: High-grade prostatic intraepithelial neoplasia
iCAF: Inflammatory cancer-associated fibroblast
mCRPC: Metastatic castration-resistant prostate cancer
min: Minutes
myCAF: Myofibroblastic cancer-associated fibroblast
NEPC: Neuroendocrine prostate cancer
nonCM: Non-conditioned media
PAAD: Pancreatic ductal adenocarcinoma
PAGE4: Prostate-associated gene 4
PCA: Principal component analysis
PCa: Prostate cancer
PCCAT: Prostate cancer cell atlas
PDX: Patient-derived xenograft
PFS: Progression-free survival
PIN: Prostatic intraepithelial neoplasia
PLL: Poly L-lysine
PRAD: Prostate adenocarcinoma
PSA: Prostate-specific antigen
qRT-PCR: Quantitative real time PCR
rpm: Revolutions per minute
RT: Room temperature
scRNA-seq: Single cell RNA sequencing
SEM: Standard error of the mean
SF-DMEM: Serum-free Dulbecco’s modified eagle medium
SMA: Alpha smooth muscle actin
SMC: Smooth muscle cells
STR: Short tandem repeat
TCP: Standard tissue culture plasticware
TGFβ: Transforming growth factor beta
TME: Tumor microenvironment
YAP1: Yes-associated protein 1
