# Supplementary material for: Unraveling the YAP1-TGFβ1 axis: a key driver of androgen receptor loss in prostate cancer-associated fibroblasts
Source: J Exp Clin Cancer Res. 2025 Dec 1;45:11. doi: 10.1186/s13046-025-03578-2 (PMC12781470; doi:10.1186/s13046-025-03578-2)
Supplement: Supplementary file 1 — Supplementary Materia 1: Supplemental Figure 1. Isolation and characterization of ex vivo culture of primary prostate fibroblasts. A) radical prostatectomy tissue wedges (i) containing regions of suspected malignancy or benign-adjacent tissue were sampled (ii) using 4 mm3 biopsy punchers. The top part of each biopsy core was removed (iii) for FFPE processing (iv) and subsequent histopathological evaluation. Remaining tissue was quartered (v) and similarly processed. B) Histopathological validation of the top section of biopsy cores from (Aiii-iv) via HE staining and p63 (brown) and AMACR (red) dual-immunohistochemistry. C) The remaining biopsy core was cut into small pieces and transferred to culture medium that supports fibroblast but not endothelial cell growth. Outgrowing fibroblasts were selectively enriched via trypsinization from any epithelial cells, which require additional collagenase treatment for detachment. D) Representative images of three primary fibroblast explant cultures isolated from different patients stained for mesenchymal markers vimentin (green) and CD90 (white) and the epithelial marker pan-cytokeratin (red). Nuclei were counterstained using Hoechst (blue). 22Rv1 PCa cells served as positive control for pan-cytokeratin (far right, upper panel). Negative control of a parallel stained fibroblast culture incubated without primary antibodies (far right, lower panel). Supplemental Figure 2. Ex vivo culture of primary prostate fibroblasts – pertaining to Supplemental Fig. 1. Single channel monochromatic images (from Supplemental Fig. 1D) of immunofluorescent validation of three primary fibroblast explant cultures isolated from different patients stained for mesenchymal markers vimentin (green) and CD90 (white) and the epithelial marker pan-cytokeratin (pan CK, red). Nuclei were counterstained using Hoechst (blue). 22Rv1 PCa cells served as a positive control (pos. ctrl) for pan-cytokeratin. Negative control (neg. ctrl) of a parallel stained fibrobl [file 13046_2025_3578_MOESM1_ESM.pdf]

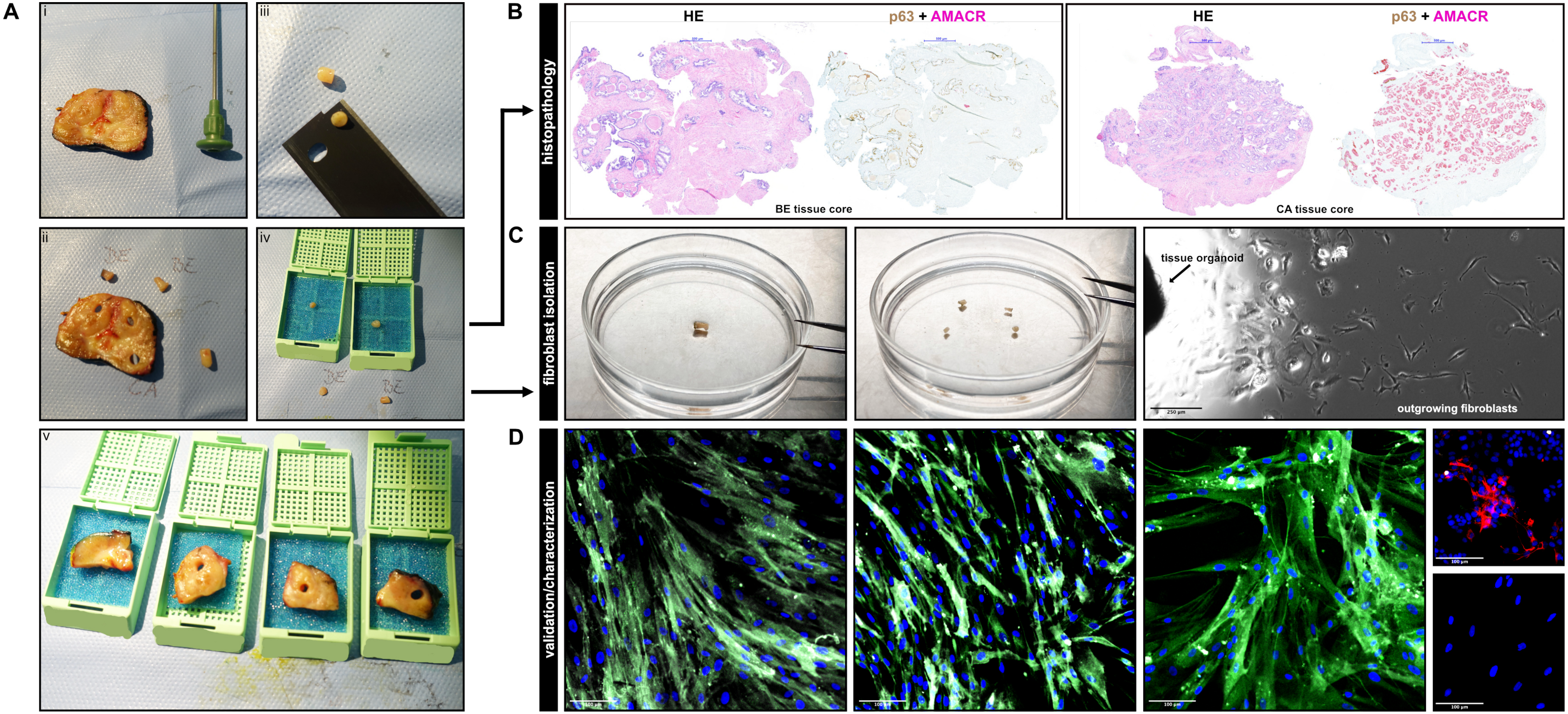

A
i
ii
iii
iv
v
B
histopathology
HE
p63 + AMACR
BE tissue core
HE
p63 + AMACR
CA tissue core
C
fibroblast isolation
tissue organoid
250 µm
outgrowing fibroblasts
D
validation/characterization
100 µm
100 µm
100 µm
100 µm
100 µm

Supp. Fig. S1

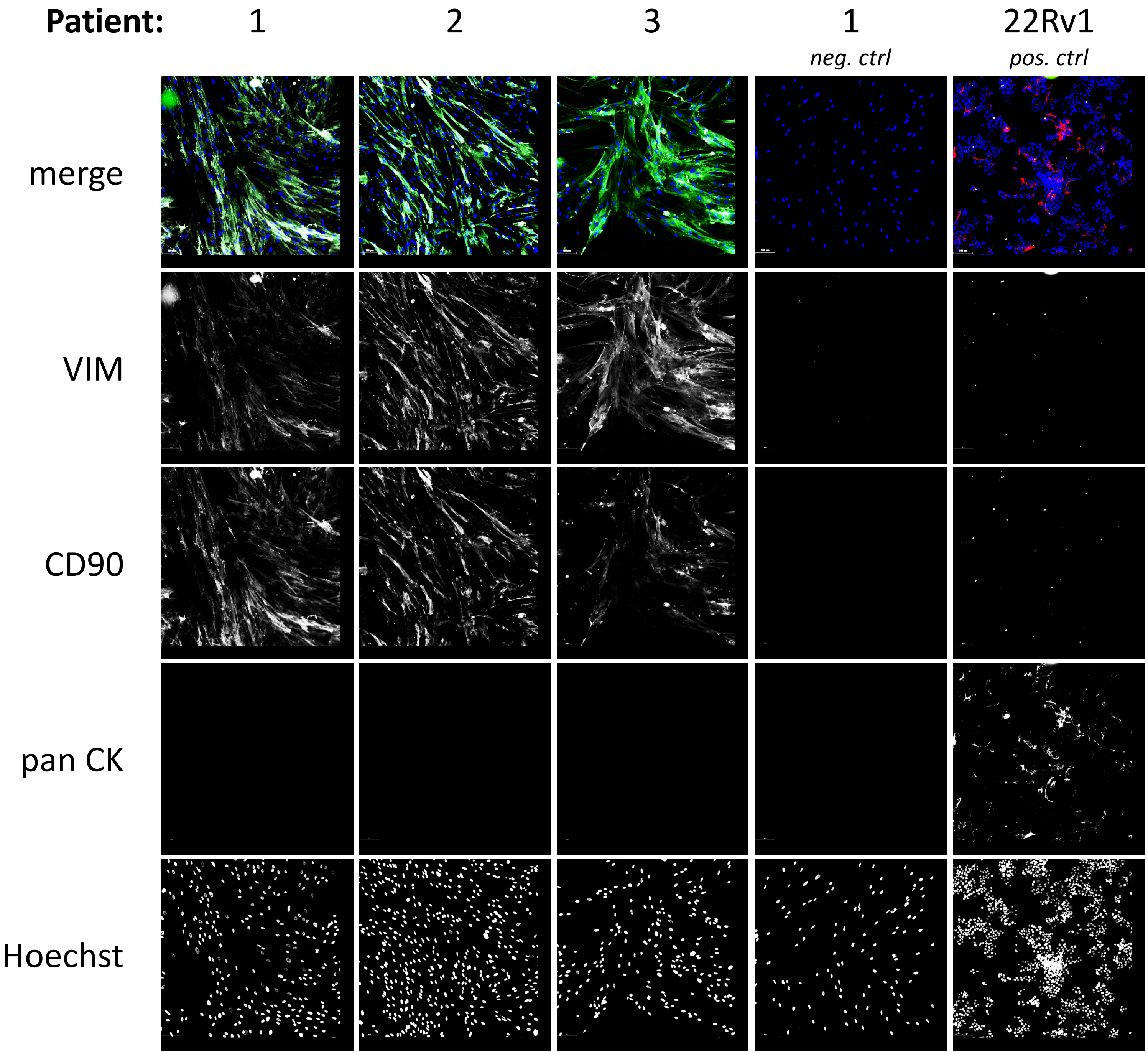

Patient:
1
2
3
1
neg. ctrl
22Rv1
pos. ctrl
merge
VIM
CD90
pan CK
Hoechst

Supp. Fig. S2

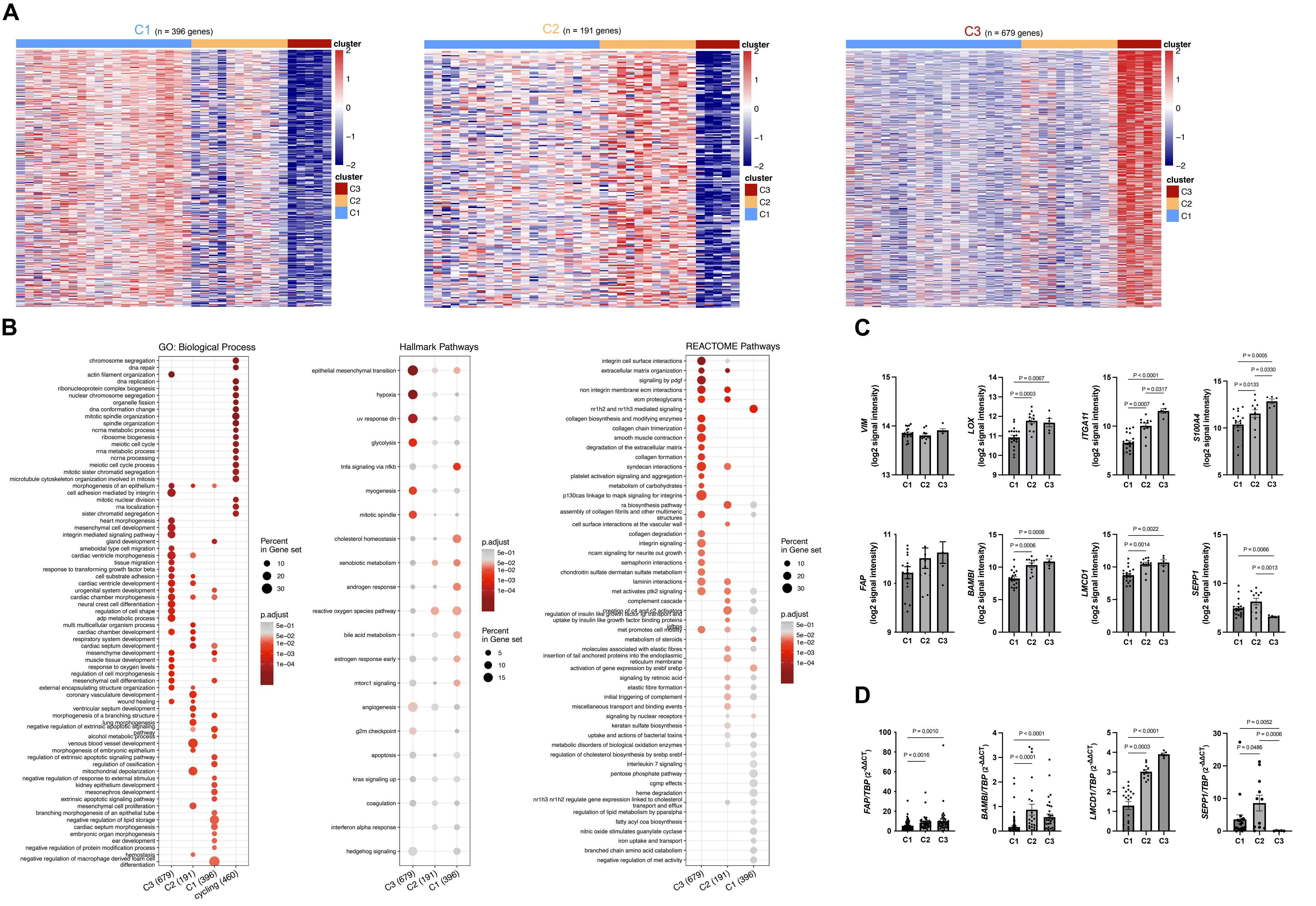

A
C1 (n = 396 genes)
C2 (n = 191 genes)
C3 (n = 679 genes)
cluster
C3
C2
C1
B
GO: Biological Process
Hallmark Pathways
REACTOME Pathways
Percent in Gene set
p.adjust
C
D
VIM (log2 signal intensity)
LOX (log2 signal intensity)
ITGA11 (log2 signal intensity)
S100A4 (log2 signal intensity)
FAP (log2 signal intensity)
BAMBI (log2 signal intensity)
LMCD1 (log2 signal intensity)
SEPP1 (log2 signal intensity)
FAP/TBP ($2^{-\Delta\Delta CT}$)
BAMBI/TBP ($2^{-\Delta\Delta CT}$)
LMCD1/TBP ($2^{-\Delta\Delta CT}$)
SEPP1/TBP ($2^{-\Delta\Delta CT}$)

Supp. Fig. S3

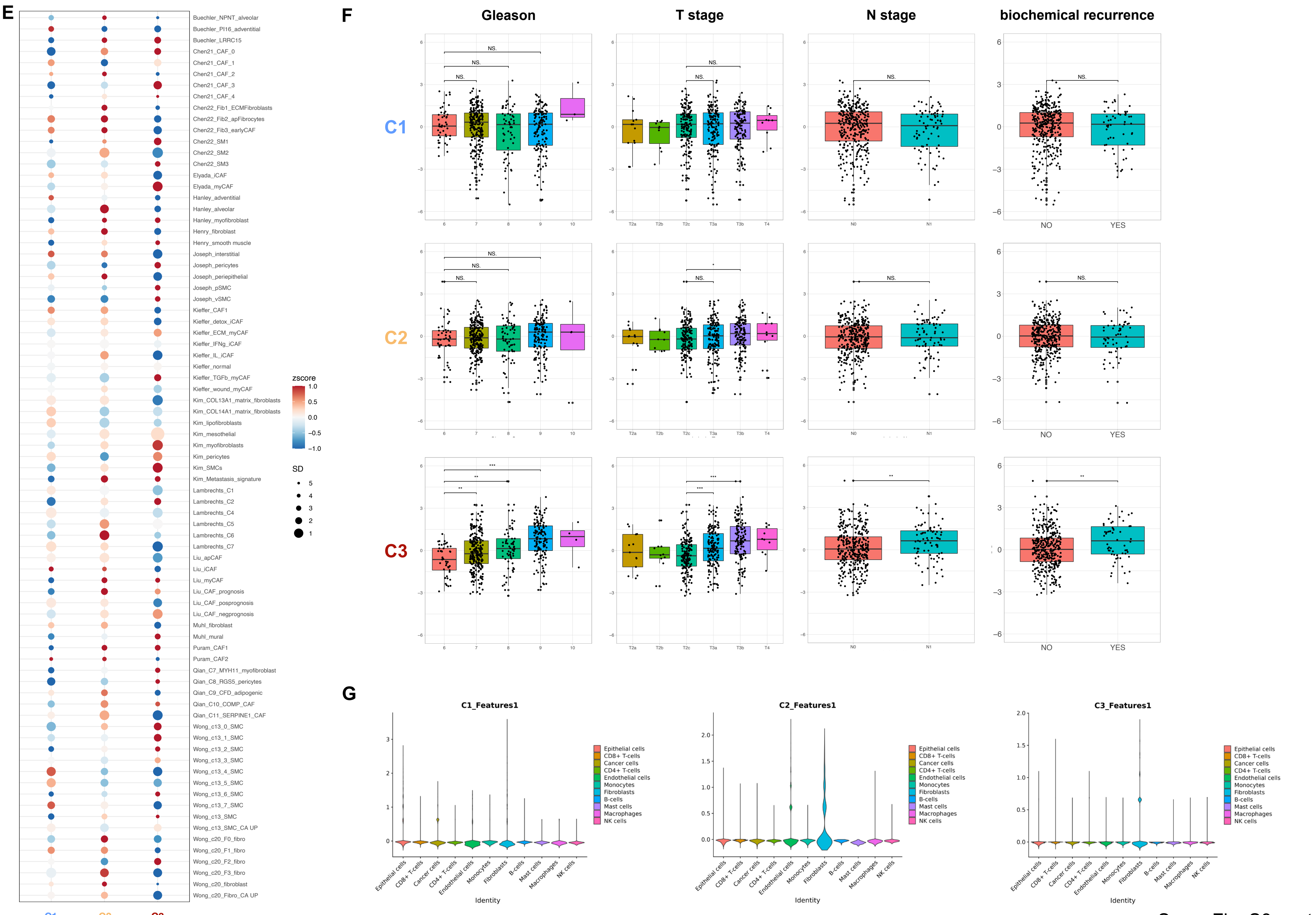

Supp. Fig. S3 cont

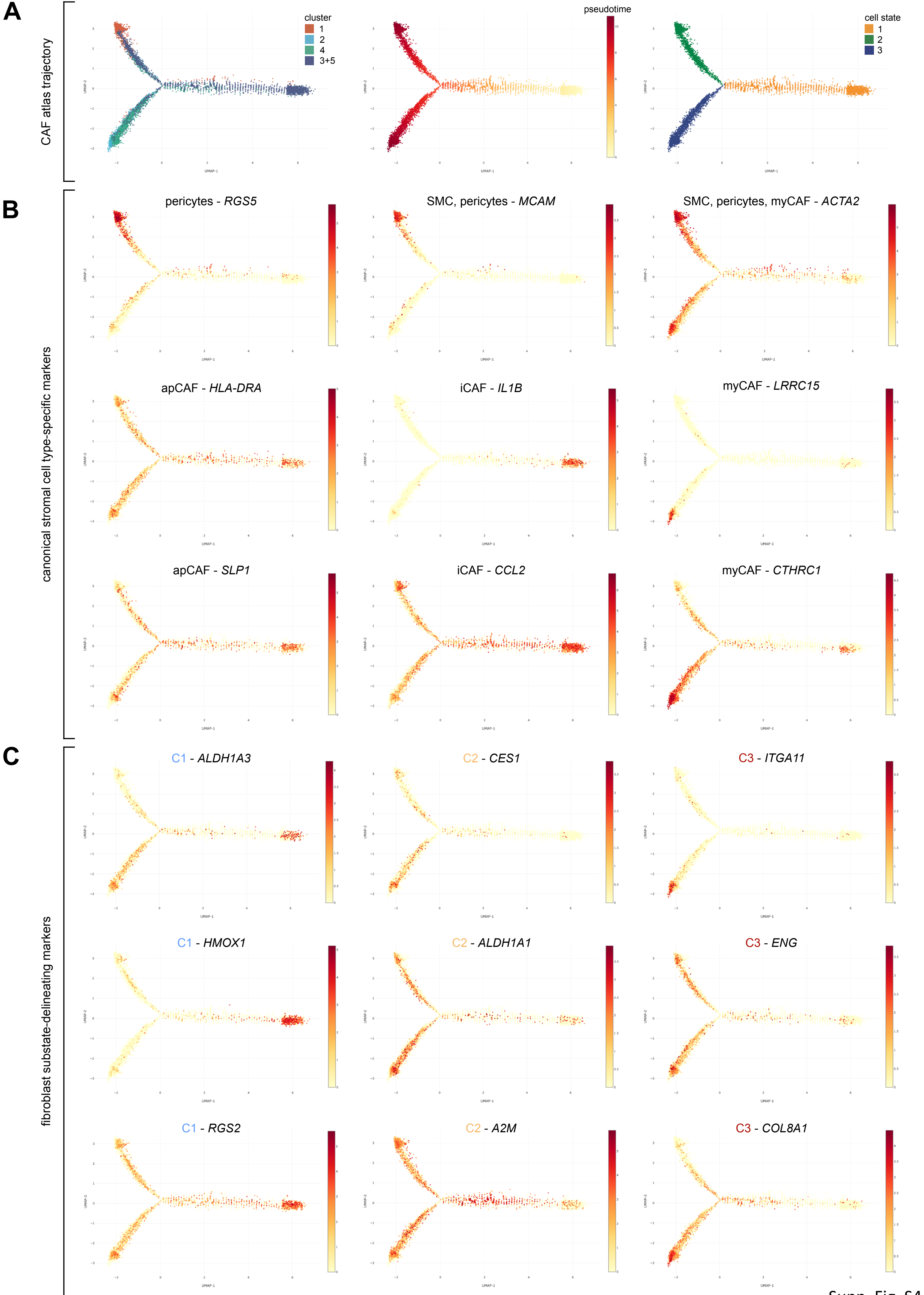
A
CAF atlas trajectory
cluster
1
2
4
3+5
pseudotime
cell state
1
2
3
UMAP-1
UMAP-2
B
canonical stromal cell type-specific markers
pericytes - RGS5
SMC, pericytes - MCAM
SMC, pericytes, myCAF - ACTA2
apCAF - HLA-DRA
iCAF - IL1B
myCAF - LRRC15
apCAF - SLP1
iCAF - CCL2
myCAF - CTHRC1
C
fibroblast substate-delineating markers
C1 - ALDH1A3
C2 - CES1
C3 - ITGA11
C1 - HMOX1
C2 - ALDH1A1
C3 - ENG
C1 - RGS2
C2 - A2M
C3 - COL8A1

Supp. Fig. S4

Supp. Fig. S5

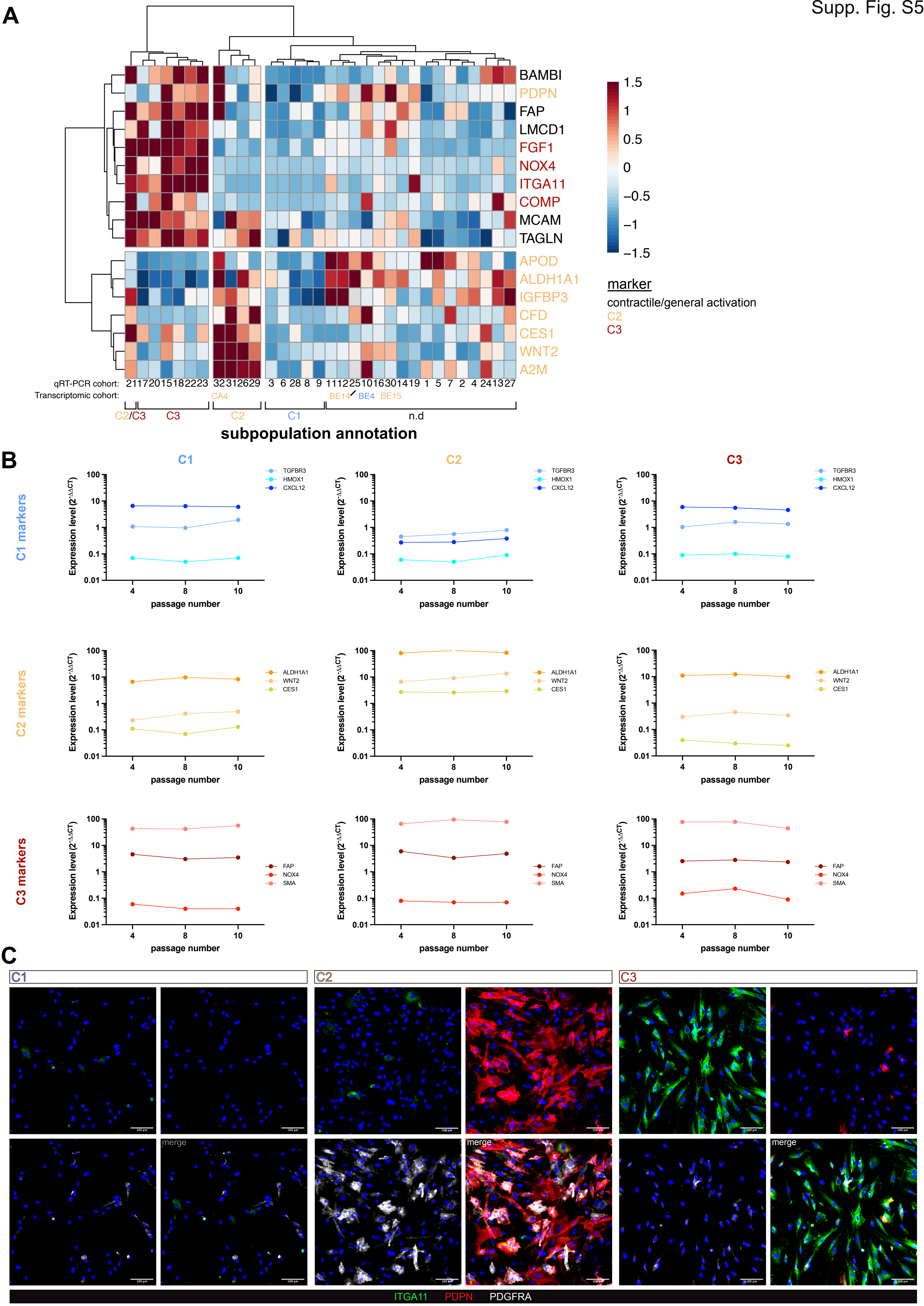
A
BAMBI
PDPN
FAP
LMCD1
FGF1
NOX4
ITGA11
COMP
MCAM
TAGLN
APOD
ALDH1A1
IGFBP3
CFD
CES1
WNT2
A2M
1.5
1
0.5
0
−0.5
−1
−1.5
marker
contractile/general activation
C2
C3
qRT-PCR cohort: 21 17 20 15 18 22 23 32 31 26 29 3 6 28 8 9 11 12 25 10 16 30 14 19 1 5 7 2 4 24 13 27
Transcriptomic cohort:
CA4
BE14
BE4
BE15
C2/C3
C3
C2
C1
n.d
subpopulation annotation
B
C1
C2
C3
C1 markers
C2 markers
C3 markers
Expression level (2^-ΔΔCT)
passage number
TGFBR3
HMOX1
CXCL12
ALDH1A1
WNT2
CES1
FAP
NOX4
SMA
C
C1
C2
C3
merge
ITGA11
PDPN
PDGFRA

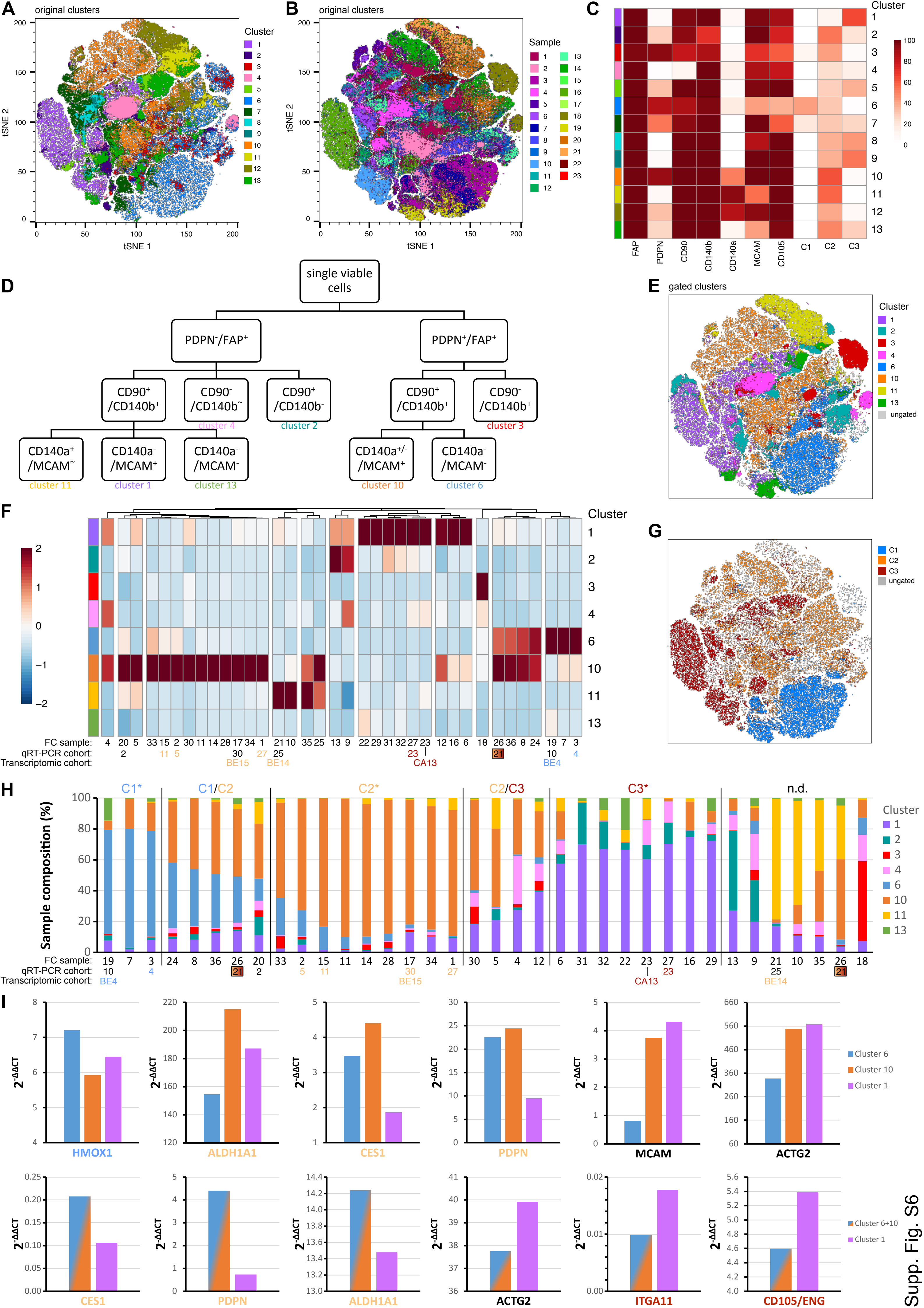
A original clusters
tSNE 1
tSNE 2
Cluster
B original clusters
Sample
C Cluster
FAP
PDPN
CD90
CD140b
CD140a
MCAM
CD105
C1
C2
C3
D single viable cells
PDPN-/FAP+
PDPN+/FAP+
CD90+ /CD140b+
CD90- /CD140b~
cluster 4
CD90+ /CD140b-
cluster 2
CD90+ /CD140b+
CD90- /CD140b+
cluster 3
CD140a+ /MCAM~
cluster 11
CD140a- /MCAM+
cluster 1
CD140a- /MCAM-
cluster 13
CD140a+/- /MCAM+
cluster 10
CD140a- /MCAM-
cluster 6
E gated clusters
Cluster
ungated
F Cluster
FC sample:
qRT-PCR cohort:
Transcriptomic cohort:
BE15
BE14
CA13
BE4
G C1
C2
C3
ungated
H C1*
C1/C2
C2*
C2/C3
C3*
n.d.
Sample composition (%)
Cluster
FC sample:
qRT-PCR cohort:
Transcriptomic cohort:
BE4
BE15
CA13
BE14
I 2-ΔΔCT
HMOX1
ALDH1A1
CES1
PDPN
MCAM
ACTG2
Cluster 6
Cluster 10
Cluster 1
CES1
PDPN
ALDH1A1
ACTG2
ITGA11
CD105/ENG
Cluster 6+10
Cluster 1

Supp. Fig. S6

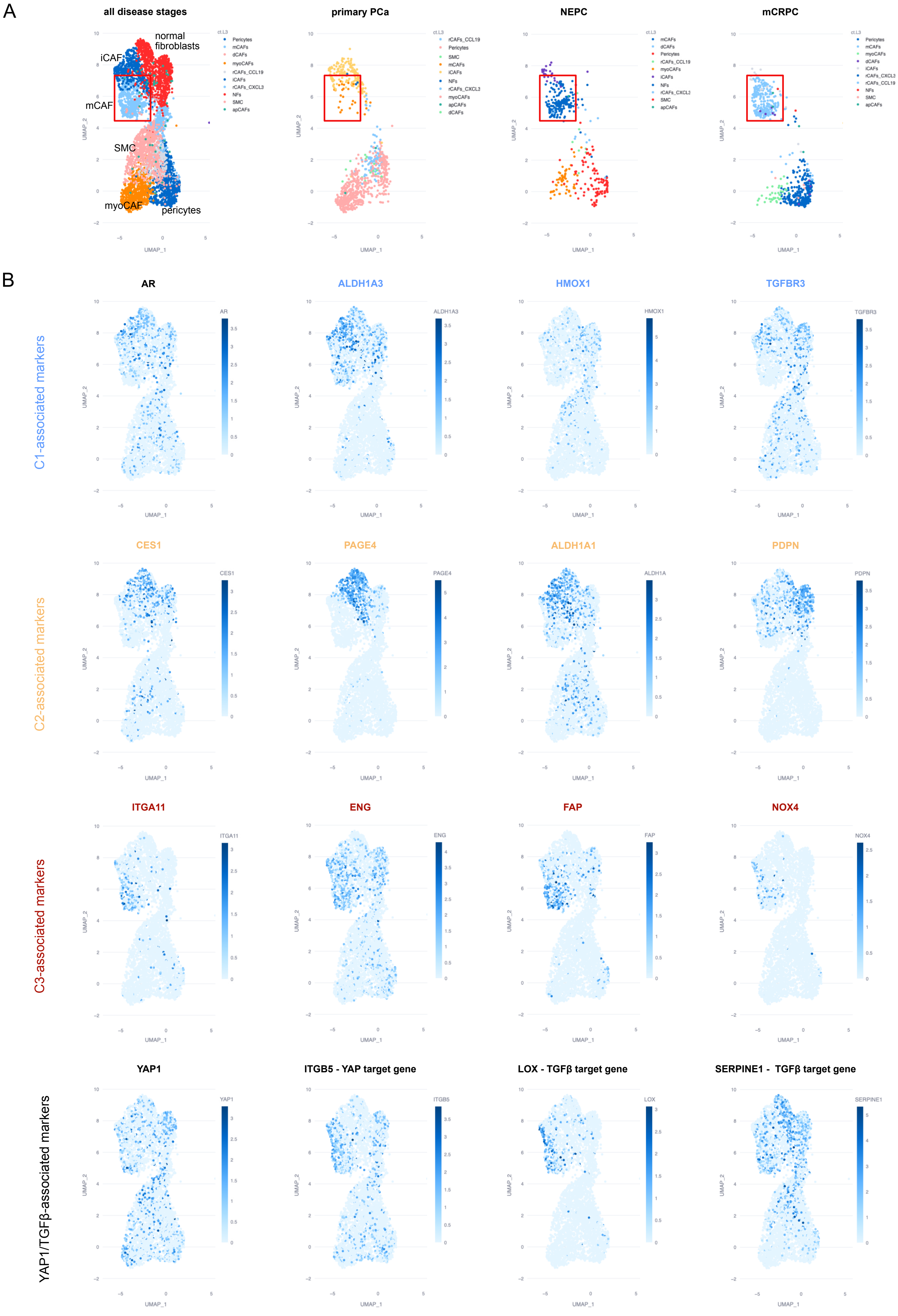
A
all disease stages
primary PCa
NEPC
mCRPC
normal fibroblasts
iCAF
mCAF
SMC
myoCAF
pericytes
ct.L3
Pericytes
mCAFs
dCAFs
myoCAFs
rCAFs_CCL19
iCAFs
rCAFs_CXCL3
NFs
SMC
apCAFs
UMAP_1
UMAP_2
B
C1-associated markers
AR
ALDH1A3
HMOX1
TGFBR3
C2-associated markers
CES1
PAGE4
ALDH1A1
PDPN
C3-associated markers
ITGA11
ENG
FAP
NOX4
YAP1/TGFβ-associated markers
YAP1
ITGB5 - YAP target gene
LOX - TGFβ target gene
SERPINE1 - TGFβ target gene

Supp. Fig. 7

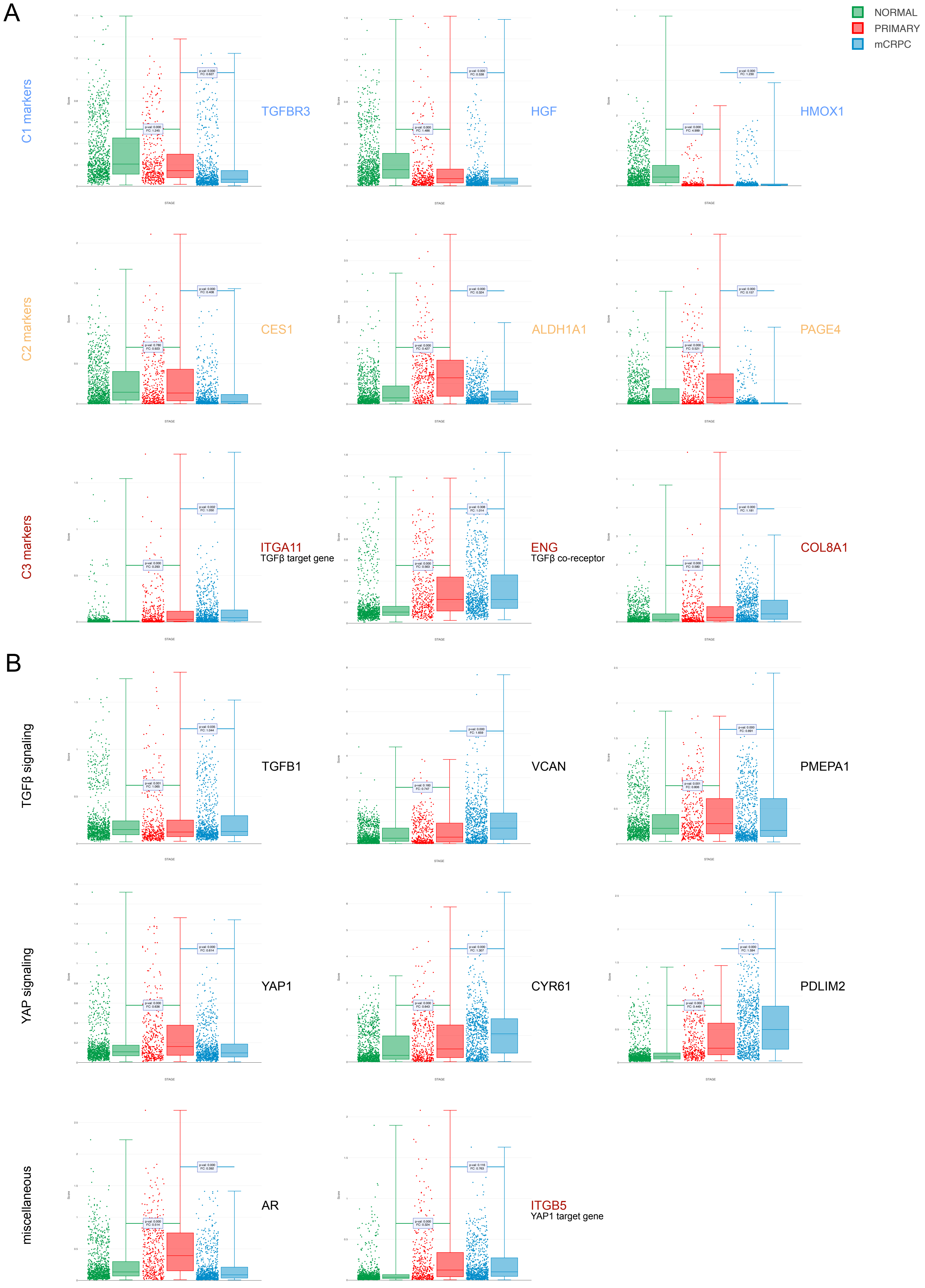

A
NORMAL
PRIMARY
mCRPC
C1 markers
TGFBR3
HGF
HMOX1
C2 markers
CES1
ALDH1A1
PAGE4
C3 markers
ITGA11
TGFβ target gene
ENG
TGFβ co-receptor
COL8A1
B
TGFβ signaling
TGFB1
VCAN
PMEPA1
YAP signaling
YAP1
CYR61
PDLIM2
miscellaneous
AR
ITGB5
YAP1 target gene
STAGE
Score

Supp. Fig. S8

A

model	mutation	phenotype
T-ERG	*Tmprss2-ERG* knock-in	mild epithelial phenotype, model of PCa initiation
Hi-MYC	*Tg(ARR2/Pbsn-MYC)7Key*	PIN with subsequent invasion
NP	*Nkx3.1creERT2;Pten$^{f/f}$*	
PRN	*Pb-Cre4$^{+/-}$;Pten$^{f/f}$;LSL-MYCN$^{+/+}$*	invasive adenocarcinoma and neuroendocrine PCa

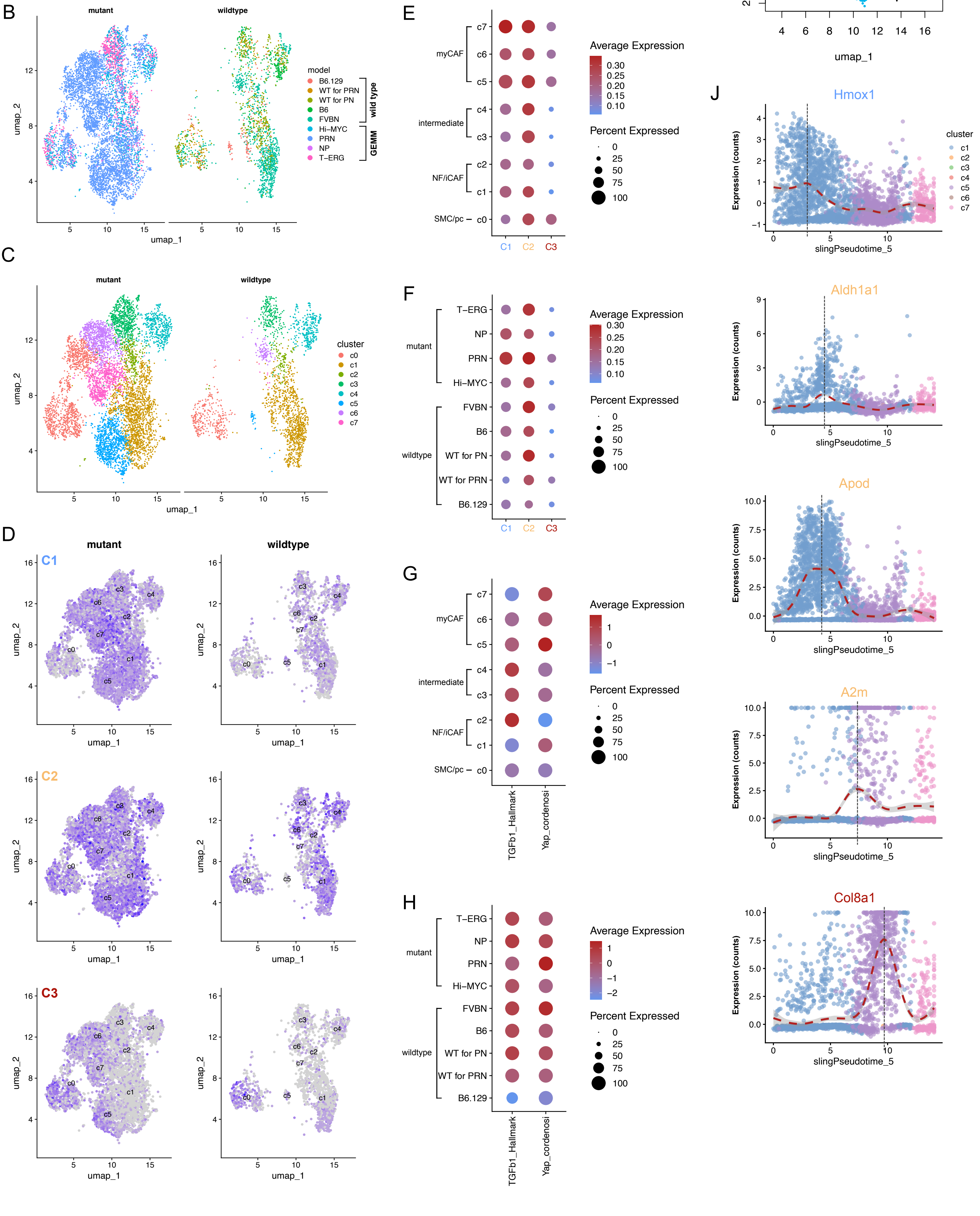

Supp. Fig. S9

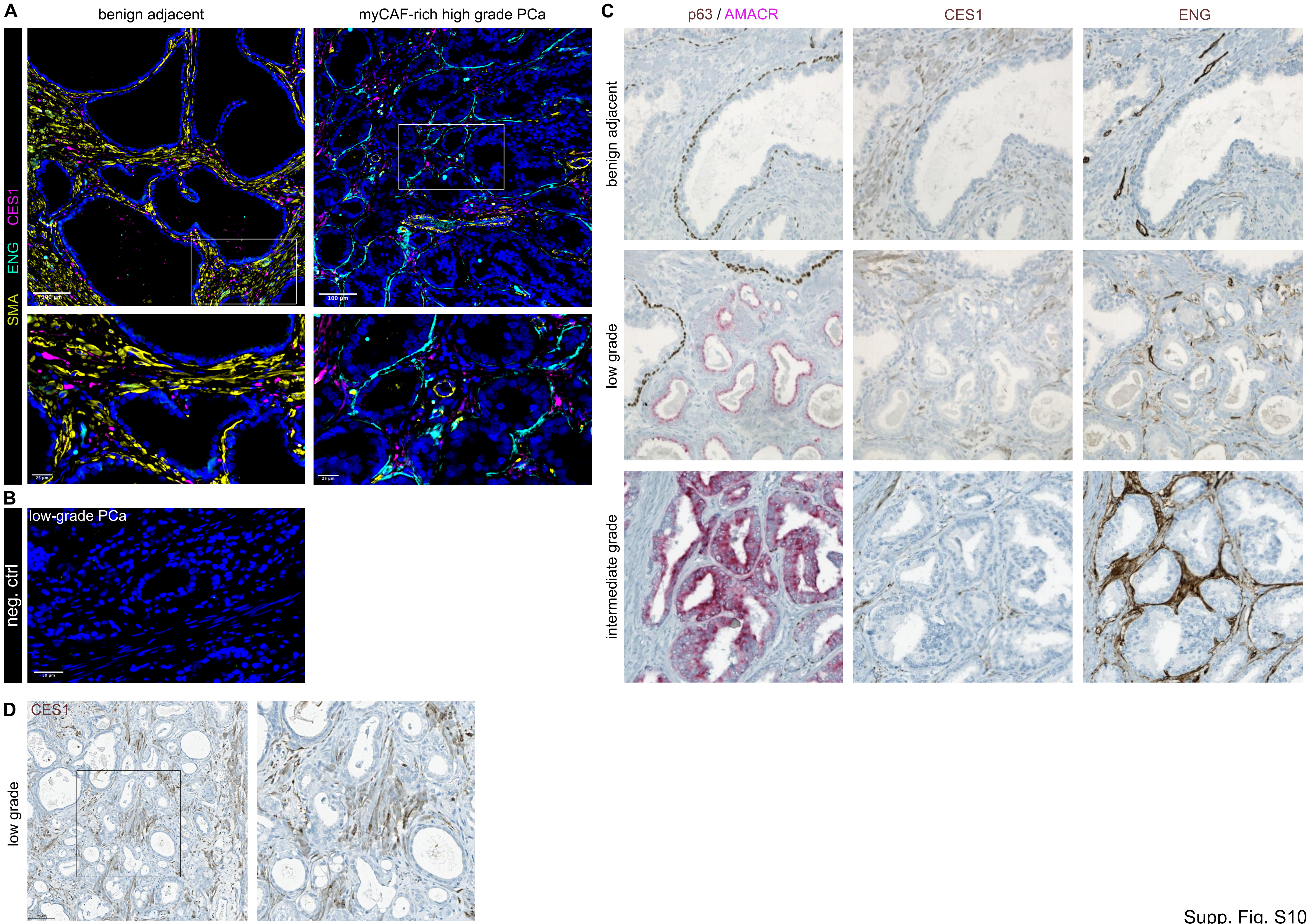
A
benign adjacent
myCAF-rich high grade PCa
SMA ENG CES1
100 µm
25 µm
B
low-grade PCa
neg. ctrl
50 µm
C
p63 / AMACR
CES1
ENG
benign adjacent
low grade
intermediate grade
D
CES1
low grade

Supp. Fig. S10

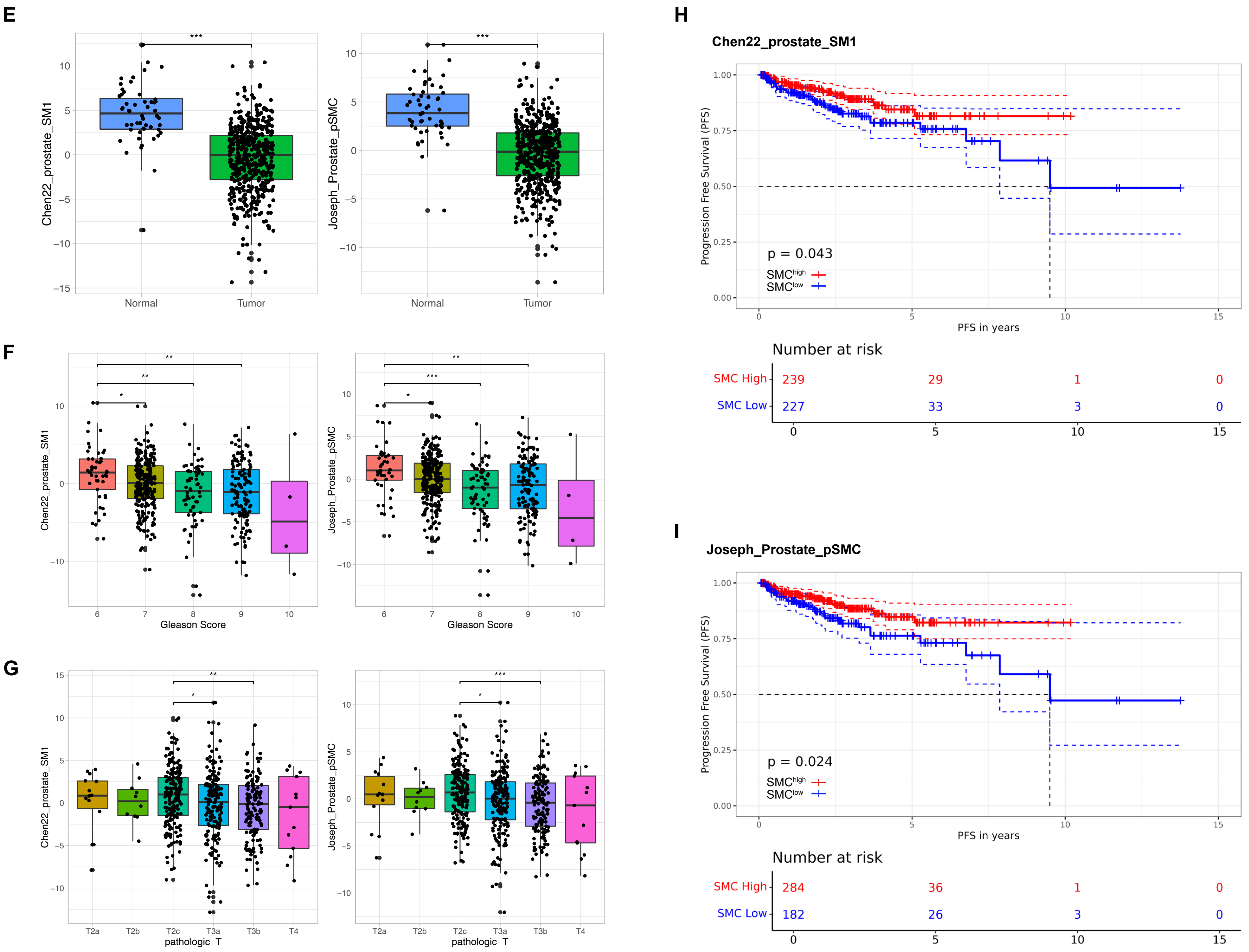

Supp. Fig. S10 cont.

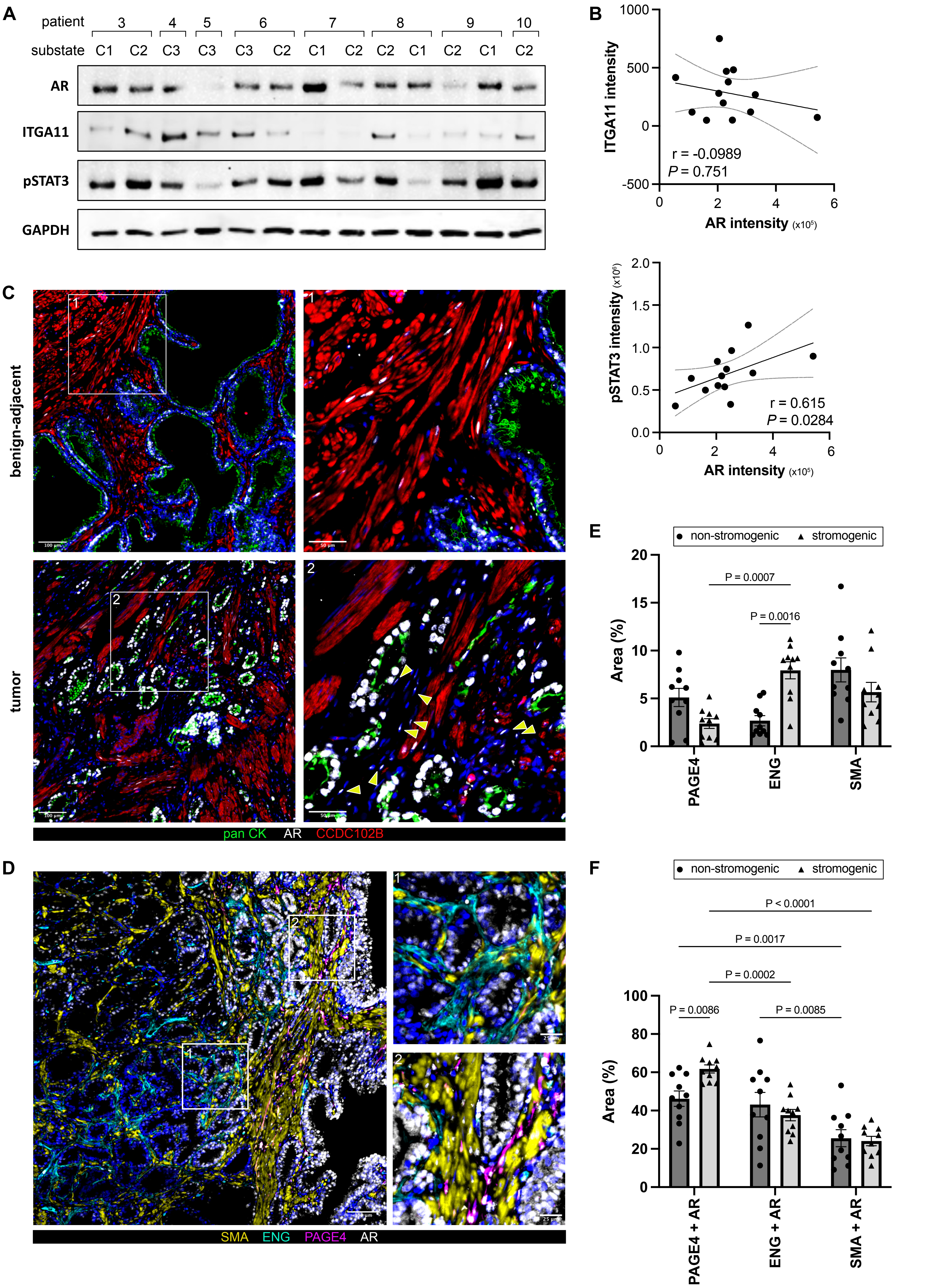

A
patient
3
4
5
6
7
8
9
10
substate
C1 C2 C3 C3 C3 C2 C1 C2 C2 C1 C2 C1 C2
AR
ITGA11
pSTAT3
GAPDH
B
ITGA11 intensity
r = -0.0989
P = 0.751
AR intensity (x10⁵)
pSTAT3 intensity (x10⁶)
r = 0.615
P = 0.0284
AR intensity (x10⁵)
C
benign-adjacent
tumor
pan CK AR CCDC102B
D
SMA ENG PAGE4 AR
E
non-stromogenic stromogenic
P = 0.0007
P = 0.0016
Area (%)
PAGE4
ENG
SMA
F
non-stromogenic stromogenic
P < 0.0001
P = 0.0017
P = 0.0002
P = 0.0086
P = 0.0085
Area (%)
PAGE4 + AR
ENG + AR
SMA + AR

Supp. Fig. S11

G

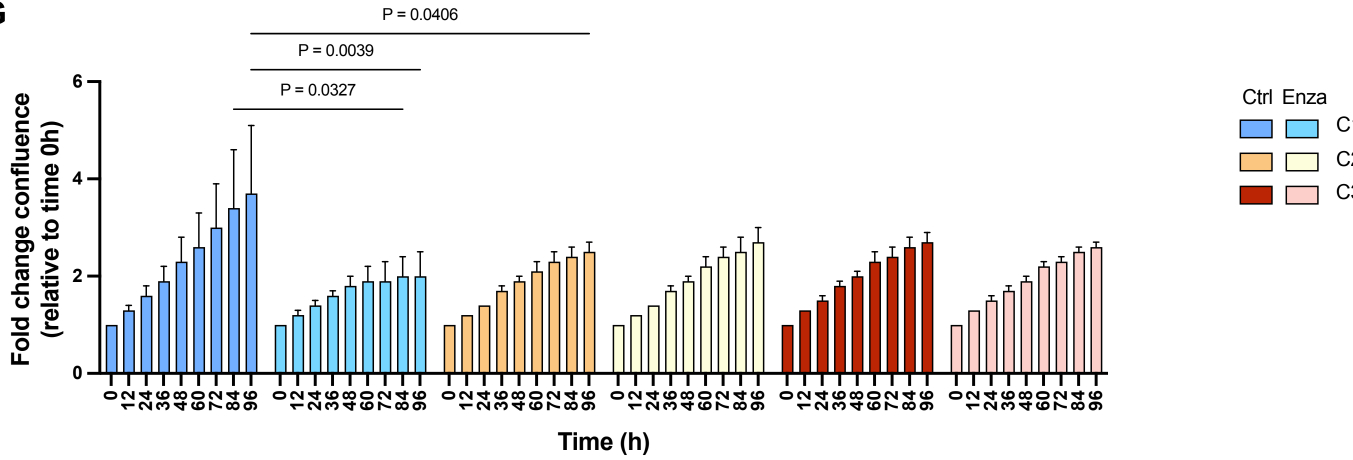
P = 0.0406
P = 0.0039
P = 0.0327
Fold change confluence (relative to time 0h)
Time (h)
Ctrl Enza
C1
C2
C3

H

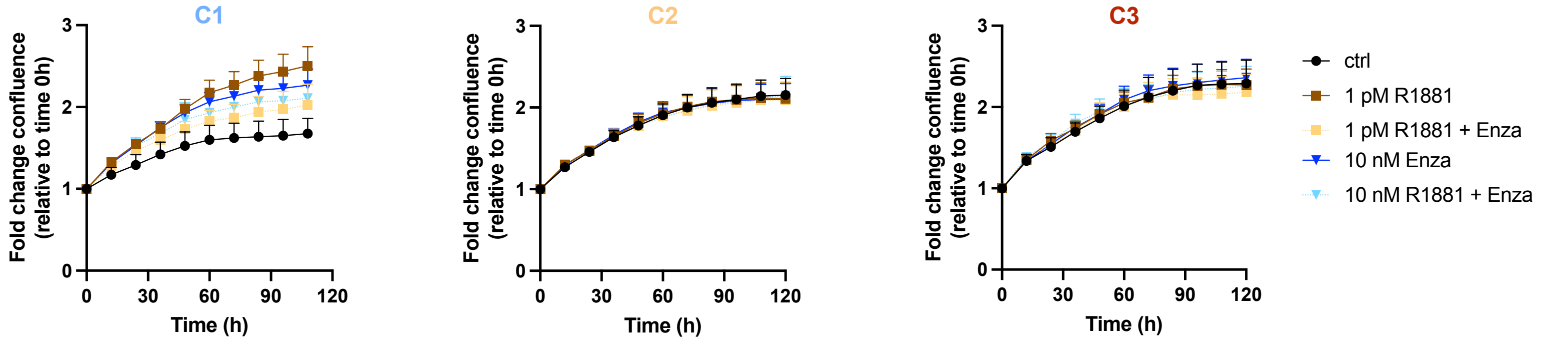
C1
C2
C3
Fold change confluence (relative to time 0h)
Time (h)
ctrl
1 pM R1881
1 pM R1881 + Enza
10 nM Enza
10 nM R1881 + Enza

I

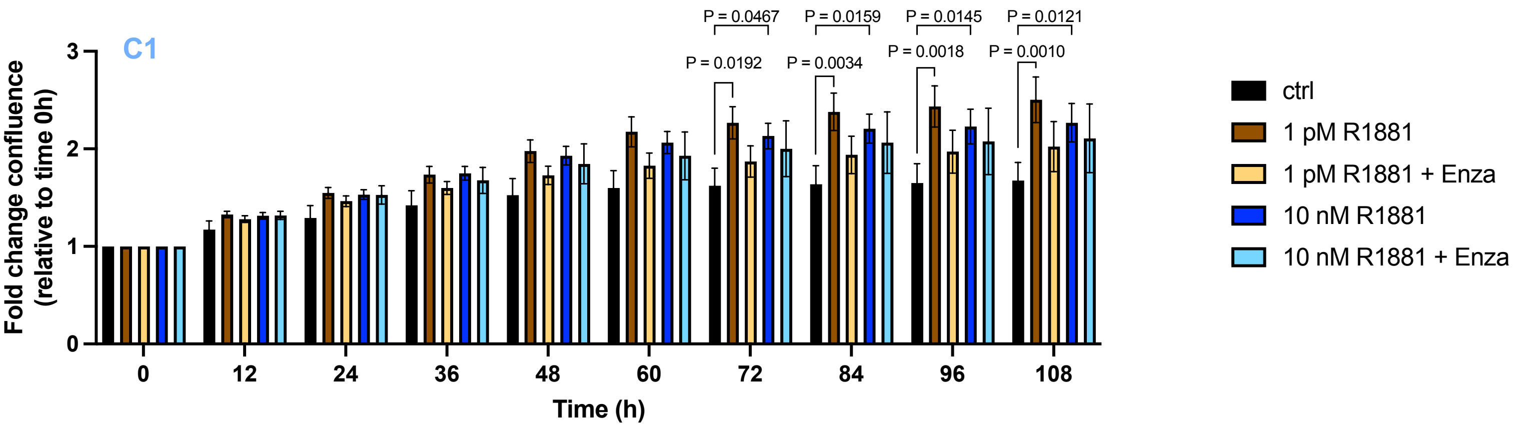
C1
P = 0.0467
P = 0.0159
P = 0.0145
P = 0.0121
P = 0.0192
P = 0.0034
P = 0.0018
P = 0.0010
Fold change confluence (relative to time 0h)
Time (h)
ctrl
1 pM R1881
1 pM R1881 + Enza
10 nM R1881
10 nM R1881 + Enza

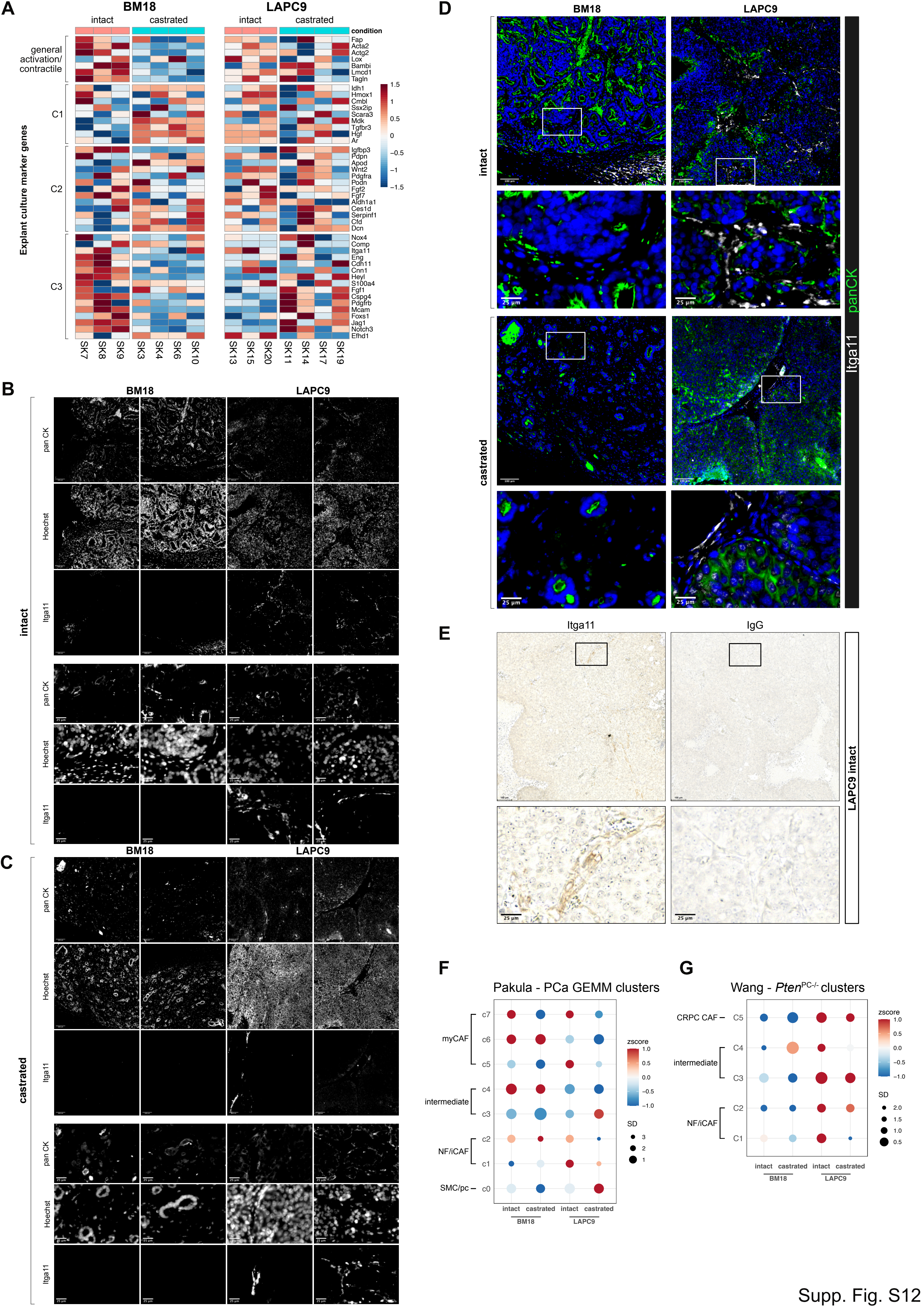
A
BM18
LAPC9
intact
castrated
condition
general activation/ contractile
C1
C2
C3
Explant culture marker genes
Fap
Acta2
Actg2
Lox
Bambi
Lmcd1
Tagln
Idh1
Hmox1
Cmbl
Ssx2ip
Scara3
Mdk
Tgfbr3
Hgf
Ar
Igfbp3
Pdpn
Apod
Wnt2
Pdgfra
Podn
Fgf2
Fgf7
Aldh1a1
Ces1d
Serpinf1
Cfd
Dcn
Nox4
Comp
Itga11
Eng
Cdh11
Cnn1
Heyl
S100a4
Fgf1
Cspg4
Pdgfrb
Mcam
Foxs1
Jag1
Notch3
Efhd1
1.5
1
0.5
0
−0.5
−1
−1.5
SK7
SK8
SK9
SK3
SK4
SK6
SK10
SK13
SK15
SK20
SK11
SK14
SK17
SK19
B
C
pan CK
Hoechst
Itga11
25 µm
D
panCK
Itga11
100 µm
E
Itga11
IgG
LAPC9 intact
F
Pakula - PCa GEMM clusters
myCAF
intermediate
NF/iCAF
SMC/pc
c7
c6
c5
c4
c3
c2
c1
c0
zscore
1.0
0.5
0.0
−0.5
−1.0
SD
3
2
1
G
Wang - Pten PC-/- clusters
CRPC CAF
C5
C4
C3
C2
C1
2.0
1.5
0.5

Supp. Fig. S12

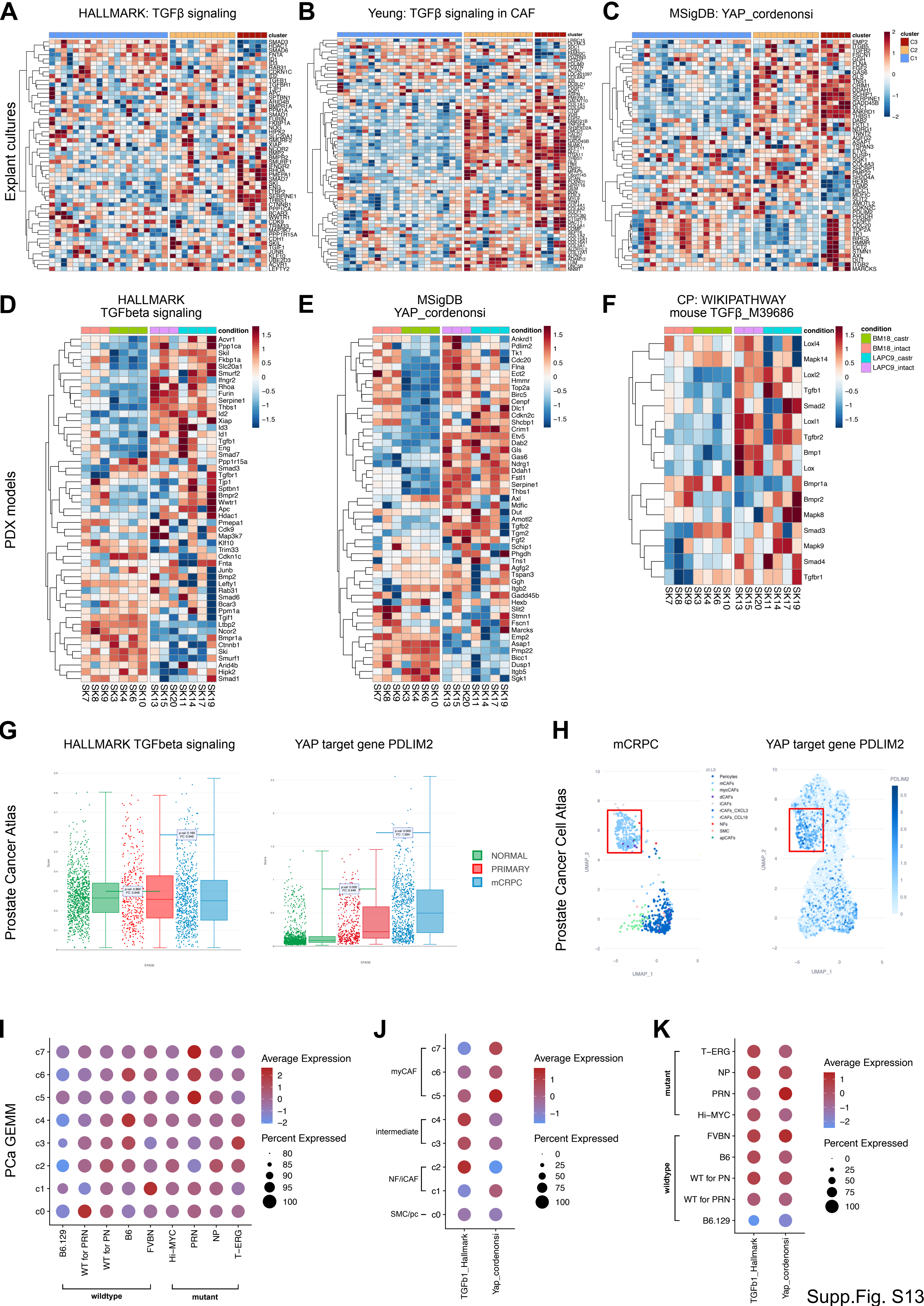
A
HALLMARK: TGFβ signaling
B
Yeung: TGFβ signaling in CAF
C
MSigDB: YAP_cordenonsi
cluster
C3
C2
C1
Explant cultures
D
HALLMARK
TGFbeta signaling
E
MSigDB
YAP_cordenonsi
F
CP: WIKIPATHWAY
mouse TGFβ_M39686
condition
BM18_castr
BM18_intact
LAPC9_castr
LAPC9_intact
PDX models
Acvr1
Ppp1ca
Skil
Fkbp1a
Slc20a1
Smurf2
Ifngr2
Rhoa
Furin
Serpine1
Thbs1
Id2
Xiap
Id3
Id1
Tgfb1
Eng
Smad7
Ppp1r15a
Smad3
Tgfbr1
Tjp1
Sptbn1
Bmpr2
Wwtr1
Apc
Hdac1
Pmepa1
Cdk9
Map3k7
Klf10
Trim33
Cdkn1c
Fnta
Junb
Bmp2
Lefty1
Rab31
Smad6
Bcar3
Ppm1a
Tgif1
Ltbp2
Ncor2
Bmpr1a
Ctnnb1
Ski
Smurf1
Arid4b
Hipk2
Smad1
Ankrd1
Pdlim2
Tk1
Cdc20
Flna
Hmmr
Top2a
Birc5
Cenpf
Dlc1
Cdkn2c
Shcbp1
Crim1
Etv5
Dab2
Gls
Gas6
Ndrg1
Ddah1
Fstl1
Serpine1
Thbs1
Axl
Mdfic
Dut
Amotl2
Tgfb2
Tgm2
Fgf2
Schip1
Phgdh
Tns1
Agfg2
Tspan3
Ggh
Itgb2
Gadd45b
Hexb
Slit2
Stmn1
Fscn1
Marcks
Emp2
Asap1
Pmp22
Bicc1
Dusp1
Itgb5
Sgk1
Loxl4
Mapk14
Loxl2
Tgfb1
Smad2
Loxl1
Tgfbr2
Bmp1
Lox
Bmpr1a
Bmpr2
Mapk8
Smad3
Mapk9
Smad4
Tgfbr1
SK7 SK8 SK9 SK3 SK4 SK6 SK10 SK13 SK15 SK20 SK11 SK14 SK17 SK19
G
HALLMARK TGFbeta signaling
YAP target gene PDLIM2
NORMAL
PRIMARY
mCRPC
Prostate Cancer Cell Atlas
H
mCRPC
YAP target gene PDLIM2
Pericytes
mCAFs
myoCAFs
dCAFs
iCAFs
rCAFs_CXCL3
rCAFs_CCL19
NFs
SMC
apCAFs
PDLIM2
UMAP_1
UMAP_2
I
PCa GEMM
c7
c6
c5
c4
c3
c2
c1
c0
B6.129
WT for PRN
WT for PN
B6
FVBN
Hi−MYC
PRN
NP
T−ERG
wildtype
mutant
Average Expression
Percent Expressed
J
myCAF
intermediate
NF/iCAF
SMC/pc
TGFb1_Hallmark
Yap_cordenonsi
K
T−ERG
NP
PRN
Hi−MYC
FVBN
B6
WT for PN
WT for PRN
B6.129

Supp.Fig. S13

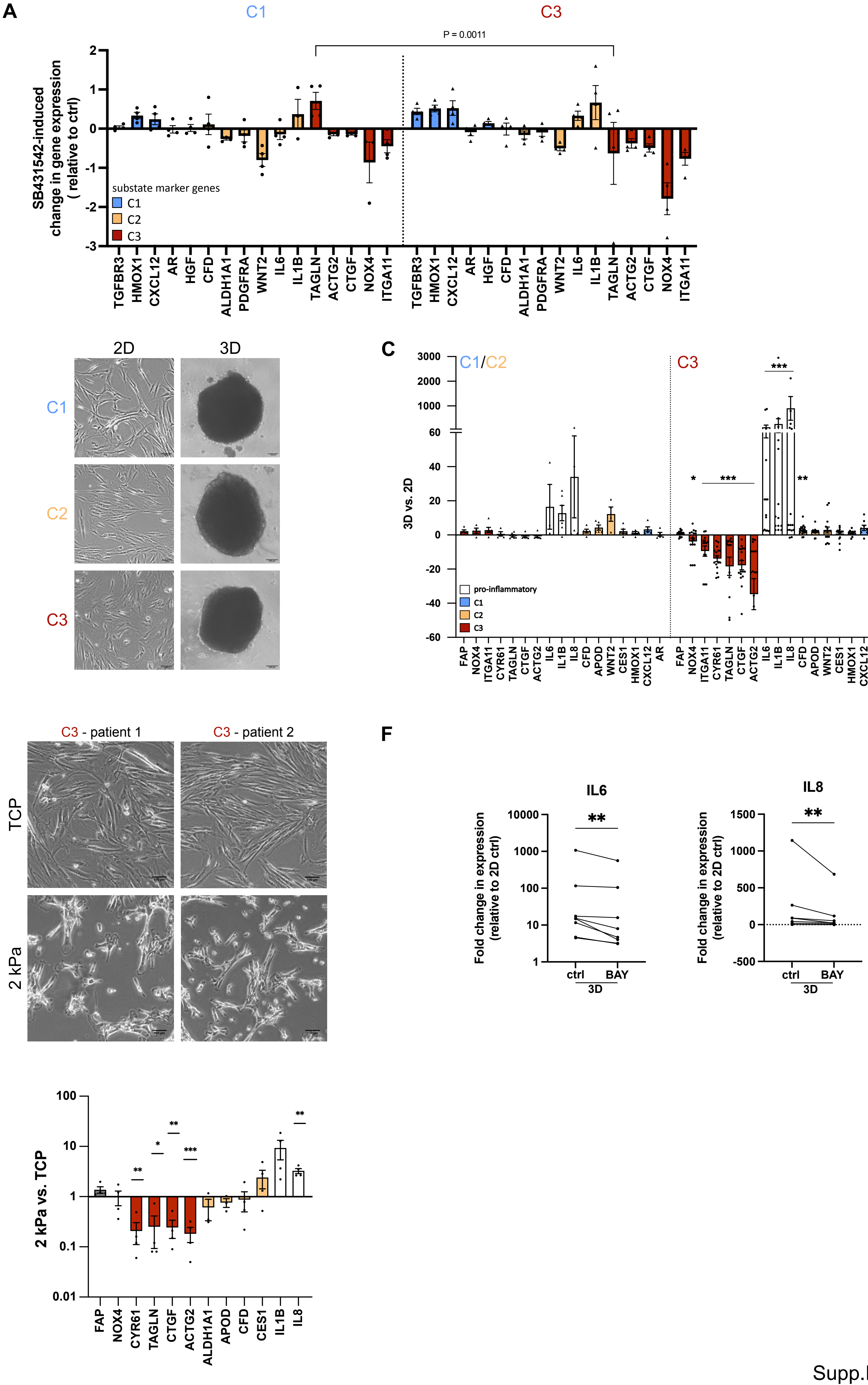

A
C1
C3
P = 0.0011
SB431542-induced change in gene expression (relative to ctrl)
substate marker genes
C1
C2
C3
TGFBR3 HMOX1 CXCL12 AR HGF CFD ALDH1A1 PDGFRA WNT2 IL6 IL1B TAGLN ACTG2 CTGF NOX4 ITGA11
B
2D
3D
C1
C2
C3
C
C1/C2
C3
3D vs. 2D
pro-inflammatory
C1
C2
C3
FAP NOX4 ITGA11 CYR61 TAGLN CTGF ACTG2 IL6 IL1B IL8 CFD APOD WNT2 CES1 HMOX1 CXCL12 AR
D
C3 - patient 1
C3 - patient 2
TCP
2 kPa
E
2 kPa vs. TCP
FAP NOX4 CYR61 TAGLN CTGF ACTG2 ALDH1A1 APOD CFD CES1 IL1B IL8
F
IL6
IL8
Fold change in expression (relative to 2D ctrl)
ctrl
BAY
3D

Supp.Fig. S14

A

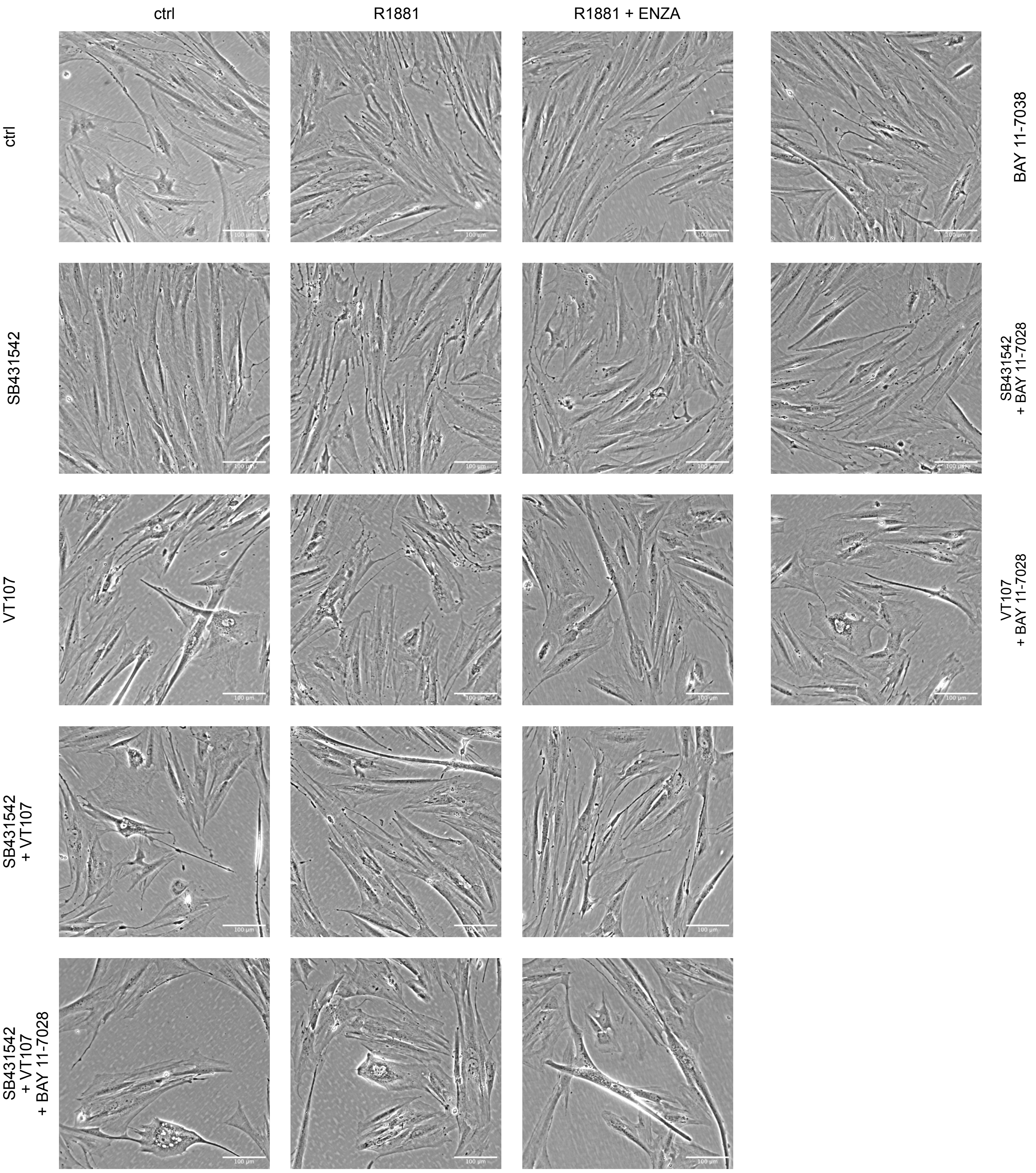
ctrl
R1881
R1881 + ENZA
ctrl
BAY 11-7038
SB431542
SB431542
+ BAY 11-7028
VT107
VT107
+ BAY 11-7028
SB431542
+ VT107
SB431542
+ VT107
+ BAY 11-7028
100 µm

B

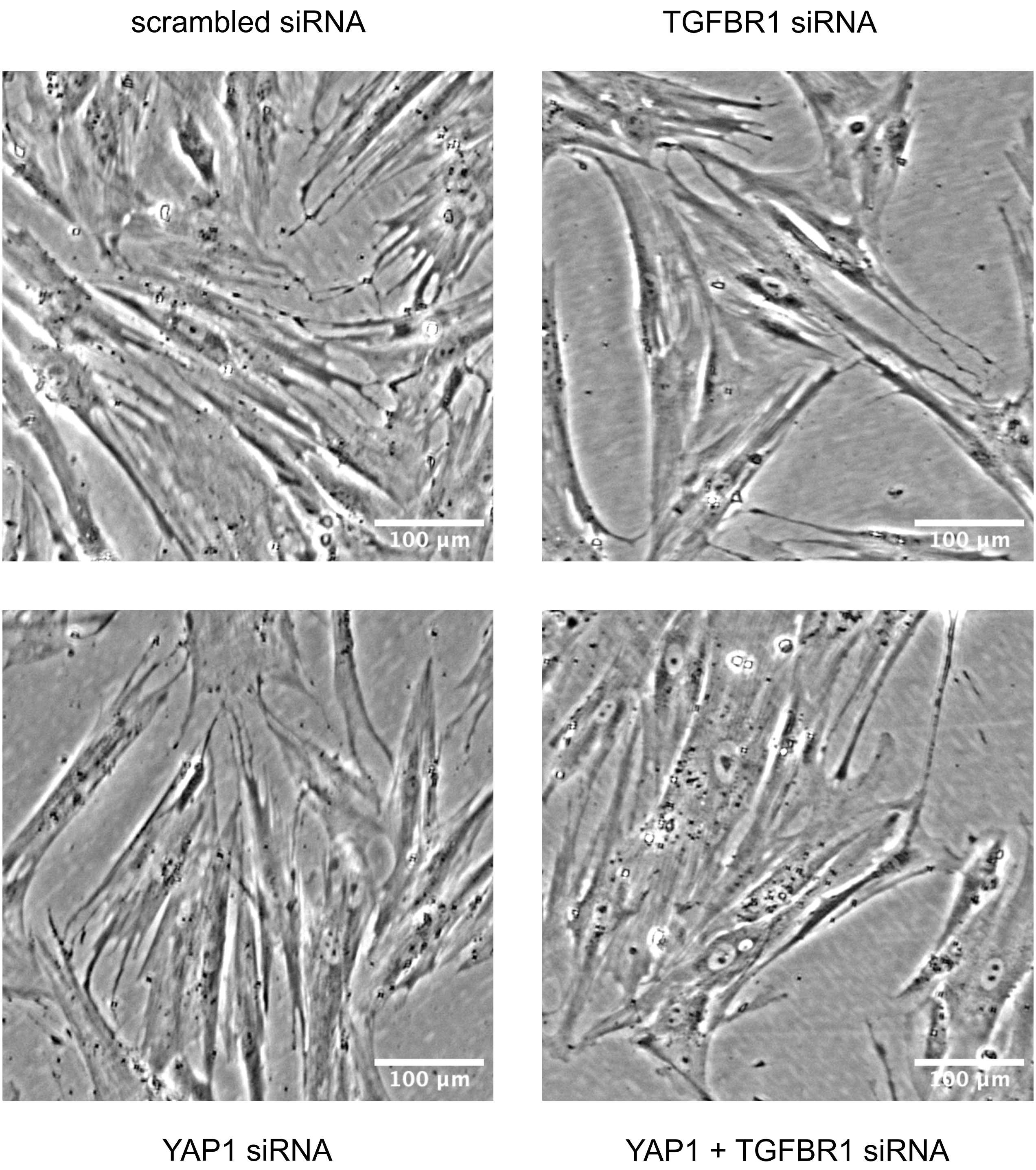
scrambled siRNA
TGFBR1 siRNA
100 µm
100 µm
100 µm
100 µm
YAP1 siRNA
YAP1 + TGFBR1 siRNA

C

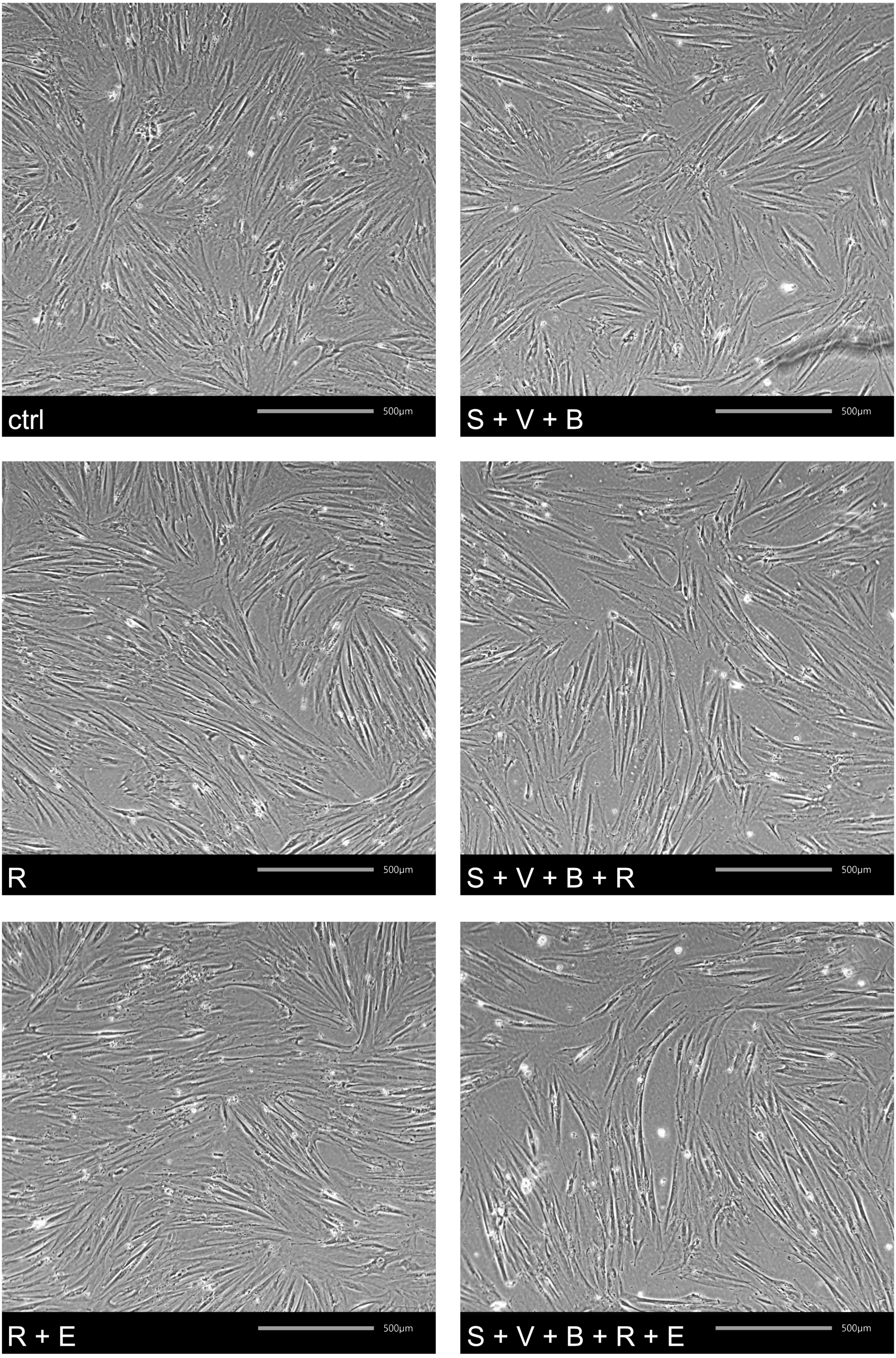

Supp. Fig. S15 cont.

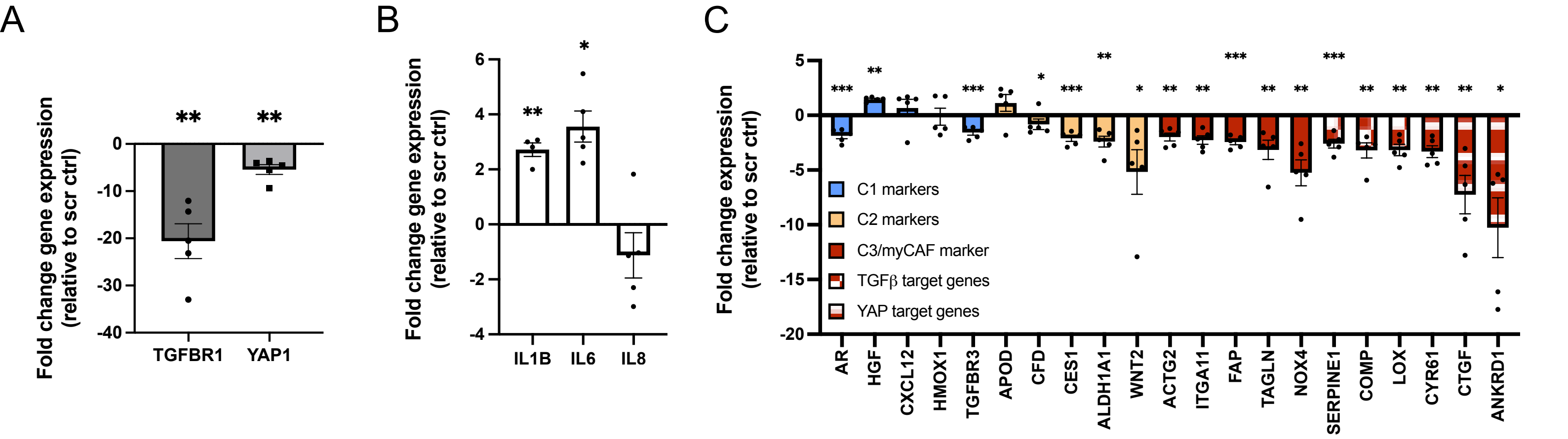
A
Fold change gene expression (relative to scr ctrl)
0
-10
-20
-30
-40
**
**
TGFBR1
YAP1
B
Fold change gene expression (relative to scr ctrl)
6
4
2
0
-2
-4
**
*
IL1B
IL6
IL8
C
Fold change expression (relative to scr ctrl)
5
0
-5
-10
-15
-20
C1 markers
C2 markers
C3/myCAF marker
TGFβ target genes
YAP target genes
AR
HGF
CXCL12
HMOX1
TGFBR3
APOD
CFD
CES1
ALDH1A1
WNT2
ACTG2
ITGA11
FAP
TAGLN
NOX4
SERPINE1
COMP
LOX
CYR61
CTGF
ANKRD1

Supp.Fig. S16
